# Isolation, Structure Determination of Sesquiterpenes from *Neurolaena lobata* and Their Antiproliferative, Cell Cycle Arrest-Inducing and Anti-Invasive Properties against Human Cervical Tumor Cells

**DOI:** 10.3390/pharmaceutics13122088

**Published:** 2021-12-05

**Authors:** Andrea Vasas, Ildikó Lajter, Norbert Kúsz, Sándor Balázs Király, Tibor Kovács, Tibor Kurtán, Noémi Bózsity, Nikolett Nagy, Zsuzsanna Schelz, István Zupkó, Georg Krupitza, Richard Frisch, Attila Mándi, Judit Hohmann

**Affiliations:** 1 Department of Pharmacognosy, Interdisciplinary Excellence Centre, University of Szeged, Eötvös u. 6., H-6720 Szeged, Hungary; vasas.andrea@szte.hu (A.V.); lajter.ildiko@pharmacognosy.hu (I.L.); kusznorbert@gmail.com (N.K.); 2 Department of Organic Chemistry, University of Debrecen, H-4032 Debrecen, Hungary; kiraly.sandor.balazs@science.unideb.hu (S.B.K.); kovacs.tibor@science.unideb.hu (T.K.); kurtan.tibor@science.unideb.hu (T.K.); 3 Department of Pharmacodynamics and Biopharmacy, Faculty of Pharmacy, University of Szeged, Eötvös u. 6, H-6720 Szeged, Hungary; bozsity-farago.noemi@szte.hu (N.B.); nagy.nikolett.abc@gmail.com (N.N.); schelz.zsuzsanna@szte.hu (Z.S.); zupko.istvan@szte.hu (I.Z.); 4 Clinical Institute of Pathology, Medical University of Vienna, Waehringer Guertel 18-20, A-1090 Vienna, Austria; georg.krupitza@meduniwien.ac.at; 5 Institute for Ethnobiology, Playa Diana, San José GT-170, Petén, Guatemala; fokuslog@gmail.com; 6 Interdisciplinary Centre of Natural Products, University of Szeged, H-6720 Szeged, Hungary

**Keywords:** *Neurolaena lobata*, Asteraceae, germacranolides, eudesmanes, isodaucane, sesquiterpenes, antiproliferative, antimigratory effect, SiHa cells

## Abstract

Seven new germacranolides (**1**–**3**, **5**–**8**), among them a heterodimer (**7**), and known germacranolide (**4**), eudesmane (**9**) and isodaucane (**10**) sesquiterpenes were isolated from the aerial parts of *Neurolaena lobata*. Their structures were determined by using a combination of different spectroscopic methods, including HR-ESIMS and 1D and 2D NMR techniques supported by DFT-NMR calculations. The enantiomeric purity of the new compounds was investigated by chiral HPLC analysis, while their absolute configurations were determined by TDDFT-ECD and OR calculations. Due to the conformationally flexible macrocycles and difficulties in assigning the relative configuration, ^13^C and ^1^H NMR chemical shift and ECD and OR calculations were performed on several stereoisomers of two derivatives. The isolated compounds (**1**–**10**) were shown to have noteworthy antiproliferative activities against three human cervical tumor cell line with different HPV status (HeLa, SiHa and C33A). Additionally, lobatolide C (**6**) exhibited substantial antiproliferative properties, antimigratory effect, and it induced cell cycle disturbance in SiHa cells.

## 1. Introduction

Sesquiterpene lactones (SLs) are one of the most prevalent and biologically significant classes of secondary metabolites of plants, comprising over 5000 known compounds. They are common in several families (e.g., Apiaceae, Solanaceae, Cactaceae and Euphorbiaceae), but the majority of them have been obtained from Asteraceae [1,2]. These lactones are classified biogenetically, according to the carbocyclic skeleton into four main groups: germacranolides, eudesmanolides, guaianolides and pseudoguaianolides. Besides these main types, there are a varieties of other lactones, formed by further modification of the carbon skeleton during biosynthesis. Germacranolides are considered as progenitors for other skeletal classes [3]. In traditionally-used medicinal plants, SLs often represents the active ingredients (e.g., achillin in *Achillea millefolium*, helenalin in *Arnica montana* and cynaropicrin in *Cynara scolymus*). Several SLs have been evaluated currently in cancer clinical trials, among these compounds, artemisinin, thapsigargin, parthenolide and its synthetic analogues that show low side effects and high antitumor potency, are promising anticancer leads or prodrugs [4]. Moreover, dimethylamino adduct of arglabin, an *Artemisia glabella* metabolite is registered as antitumor substance in the Republic of Kazakhstan [5]. In case of SLs, *α*-methylene-*γ*-lactone group is the molecular part mostly responsible for biological effects such as antitumor, anti-inflammatory and antimicrobial activities. This is a consequence of its alkylation potency acting on enzymes and transcription factors in different organisms. This ring is responsible, and often considered essential for the cytotoxic effect of SLs [6].

*Neurolaena lobata* (L.) R.Br. ex Cass. (Asteraceae) is a perennial flowering plant distributed in Central and South America and the West Indies. The leaves of the plant have been used by local communities primarily in case of malaria, parasitic ailments, and pain of various origins, but also for the treatment of inflammatory skin disorders, ulcers, different types of cancer and diabetes [7,8]. *N. lobata* is a rich source of sesquiterpene lactones. Previous phytochemical studies of the plant have led to the isolation of sesquiterpenes of germacranolide (neurolenins A-F, lobatin A and 3-*epi*-desacetylisovaleroylheliangine), seco-germacranolide (neurolobatins A and B), furanoheliangolide (lobatins B, and C, 8*β*-isovalerianyloxy-9*α*-hydroxycalyculatolide, 8*β*-isovalerianyloxy-9*α*-acetoxycalyculatolide and 5*β*-hydroxy-8*β*-isovaleroyloxy-9*α*-hydroxycalyculatolide) and eudesmanolide (3*β*-acetoxy-8*β*-isovaleroyloxyreynosin) types [9,10,11,12].

Previous pharmacological investigations of the crude leaf extract proved its anti-inflammatory [13,14], antiulcerogenic [15], antinociceptive [16], and antimicrobial [17,18,19] activities. Dichloromethane extract of the plant was tested for its antineoplastic activity in HL-60 cells and in anaplastic large cell lymphoma cell lines of human or mouse origin and antiproliferative and proapoptotic effects were detected. Furthermore, the extract induced tumor suppressors, down-regulated the expression of oncogenes, inhibited cell proliferation and triggered the apoptosis of malignant cells [20]. Moreover, neurolenin B specifically decreased pro-carcinogenic NPM/ALK expression in ALK+ ALCL cells, and attenuated tumor intra/extravasation into the lymphatics [21]. Pharmacological investigation of lobatin B revealed that this compound also inhibited the expression of NPM/ALK, and in addition, the expression of JunB and PDGF-Rβ, and attenuated proliferation of ALCL cells by arresting them in late M phase [22]. In vitro assays for cytotoxic activity of sesquiterpene lactones of *N. lobata* revealed many compounds to be effective against GLC4 human small cell lung carcinoma and COLO 320 human colorectal cancer cells. Lobatin B possessed the highest cytotoxicity (IC_50_ = 0.6 and 1.1 µM, in case of GLC4 and COLO 320, respectively), followed by neurolenin B (IC_50_ = 1.1 and 1.2 µM) [23].

The aim of the present work was the isolation of further biologically active sesquiterpenes from *N. lobata*. This paper reports the isolation and structure elucidation of seven new SLs (**1**–**3**, **5**–**8**), including a dimeric lactone (**7**), and three known sesquiterpenes (**4**, **9**, and **10**) from the CH_2_Cl_2_ extract of the frozen herb. The isolated compounds were evaluated for their antiproliferative activities against three human cervical cancer cell lines with different human papillomavirus (HPV) status including HPV-negative C33A, as well as HPV-16 and 18-positive SiHa, and HeLa, respectively. The most promising natural product (**6**) was additionally investigated in order to reveal its cancer selectivity, action on cancer cell migration and cell cycle distribution.

## 2. Materials and Methods

### 2.1. General Experimental Procedures

The high-resolution MS spectra were acquired on a Thermo Scientific Q-Exactive Plus orbitrap mass spectrometer equipped with ESI ion source in positive ionization mode. The samples were dissolved in MeOH. The data were acquired and processed with the MassLynx software. Data acquisition and analysis were accomplished with Xcalibur software version 2.0 (Thermo Fisher Scientific, Waltham, MA, USA). A Bruker Avance DRX 500 spectrometer [500 MHz (^1^H) and 125 MHz (^13^C)] was used for recording the NMR spectra. The signals of the deuterated solvent CDCl_3_ were taken as reference. 2D NMR data were acquired and processed with standard Bruker software. Gradient-enhanced versions were used in the ^1^H–^1^H COSY, HSQC and HMBC experiments. Optical rotations were determined in CHCl_3_ by using a Perkin-Elmer 341 polarimeter. ECD spectra were recorded on a JASCO J-810 spectropolarimeter.

Rotational planar chromatography (RPC) was performed by using a Chromatotron apparatus (Harrison Research, Palo Alto, CA, USA) on self-coated silica plates (Kieselgel 60 GF_254_, 15 μm, Merck, Darmstadt, Germany). For column chromatography (CC), polyamide (MP Polyamide, 50–160 μm, MP Biomedicals, Irvine, CA, USA) and silica gel (Kieselgel 60, 63–200 μm, Merck, Darmstadt, Germany) were used. Thin-layer chromatography (TLC) was carried out using silica gel (Kieselgel 60 F_254_, Merck) and RP-C18 (F_254s_, Merck) pre-coated plates. The preparative TLC (prep-TLC) was performed on pre-coated silica gel plates (20 × 20 cm^2^, Kieselgel 60 F_254_, Merck). The compounds were detected by spraying with cc. vanillin sulfuric acid, followed by heating (120 °C).

### 2.2. Plant Material

*Neurolaena lobata* (L.) R.Br. ex Cass. (Asteraceae) was collected by R. Diaz, and R. O. Frisch (Institute for Ethnobiology, Playa Diana, GT-170 San José/Petén, Guatemala), in the flowering period, in the area of the Chakmamantok-rock formation (16 59′16″ N, 89 53′45″ W) in San José, Guatemala. Voucher specimens were archived at the herbarium of the Institute for Ethnobiology, San Jose, Guatemala, and at the Herbarium of the Department of Pharmacognosy, University of Szeged, Szeged, Hungary (No. 813). The fresh plant material (aerial parts) was air-dried and stored deep-frozen until subsequent extraction.

### 2.3. Extraction and Isolation

The dried and ground aerial parts of the plant (3.00 kg) was percolated with MeOH (50 L) at room temperature. The extract was concentrated under reduced pressure and solvent–solvent partition was performed with 5 × 1 L petroleum ether (A), 5 × 1 L of CH_2_Cl_2_ (B), and finally with 5 × 1 L of EtOAc (C). The CH_2_Cl_2_ phase (95.4 g) was separated on a polyamide column (287 g) with mixtures of MeOH and H_2_O (1:4, 2:3, 3:2 and 4:1, 3 L of each) as eluents to afford seven fractions (BI–BVII). Fraction BII (33.6 g), obtained with MeOH–H_2_O (1:4), was subjected to silica gel VLC, using a gradient system of cyclohexane–EtOAc–EtOH (from 3:1:0 to 5:5:1). The fractions were combined after TLC monitoring into nine fractions (BII/1-BII/9). Fraction BII/4 (98.7 mg) was separated by RPC with a CH_2_Cl_2_–acetone gradient system (from 99:1 to 9:1, and finally MeOH). The subfraction (35.6 mg) eluted with CH_2_Cl_2_–acetone 97:3 was subjected to preparative TLC, using a system of CH_2_Cl_2_–acetone (95:5), to give compound **9** (4.6 mg). Fracion BII/7 was separated by VLC with the gradient system of CH_2_Cl_2_–acetone (from 99:1 to 4:1, and finally MeOH) to yield 12 subfraction. Subfraction 10 (767 mg) was further chromatographed by VLC with cyclohexane–EtOAc–EtOH gradient elution (from 60:20:0.5 to 6:6:1) and fraction eluted with cyclohexane–EtOAc–EtOH 60:30:0.5 (338 mg) was separated by VLC with CH_2_Cl_2_–MeOH (from 100:1 to 100:3.5). Fractions eluted with CH_2_Cl_2_–MeOH 100:1.5 was combined and finally purified by RP TLC with MeOH–H_2_O 7:3 to yield compound **10** (2.1 mg) Fraction BIII (7.6 g) obtained from polyamide column with MeOH–H_2_O (3:2), was subjected to silica gel VLC, using a gradient system of cyclohexane–EtOAc–EtOH (from 30:5:0 to 30:30:2) to yield 10 main fraction (BIII/1–10). Fraction BIII/5 (230 mg) was further purified by RP VLC with MeOH–H_2_O gradient system (from 2:3 to 9:1). Fractions eluted with MeOH–H_2_O (7:3) were combined (40 mg) and finally preparative TLC (CHCl_3_–acetone 97:3) was applied to isolate compounds **4** (1.8 mg), **3** (3.4 mg) and **1** (1.98 mg). Fraction BIII/6 (2.7 g) was firstly separated by VLC with gradient system of CHCl_3_–acetone (from 99:1 to 9:1). Fractions were combined into 8 subfractions according to their components. Subfraction 1 (32 mg) was further purified by preparative TLC with the mobile phase toluene–EtOAc 3:2, affording the isolation of compounds **5** (1.6 mg), **6** (17.4 mg) and **2** (1.5 mg). Subfraction 4 was purified first by VLC with a gradient system of cyclohexane–EtOAc–EtOH (from 3:1:0 to 30:30:3). Fractions obtained with cyclohexane–EtOAc 3:2 was combined and further purified by preparative TLC with cyclohexane–EtOAc–EtOH (30:30:1) and compound **8** (1.5 mg) was isolated. Fraction BIII/8 (0.6 g) was chromatographed on VLC with a gradient system of cyclohexane–EtOAc–EtOH (from 3:1:0 to 30:30:3) and 10 main fractions were yielded. Fraction BIII/9 (0.7 g) was separated by VLC with a gradient system of CHCl_3_–MeOH (from 99:1 to 9:1), and 9 main fractions were yielded. Fraction 5 was purified by preparative TLC with cyclohexane–EtOAc–EtOH (30:30:1) as eluent and compound **7** (2.1 mg) was isolated.

### 2.4. Physical Characteristics of New Compounds

*8β-Hydroxy-9α-isovaleroyloxycalyculatolide* (**1**): an amorphous solid; [α]^28^_D_ +12 (*c* 0.1, CHCl_3_). ECD {CH_3_CN, *λ*_max_ [nm] (Δε)}, *c* 2.84 × 10 − 4 M: 314 (+0.36), 256 (+1.72), 215 (−2.76); ^1^H NMR and ^13^C NMR data, see Table 1 and Table 2; HR-ESIMS *m*/*z* 379.1755 [M + H]^+^ (calcd for C_20_H_27_O_7_, 379.1757).

*Lobatolide A* (**2**): an amorphous solid; [α]^27^_D_ +47 (*c* 0.05, CHCl_3_); ^1^H NMR and ^13^C NMR data, see Table 1 and Table 2; HR-ESIMS *m*/*z* 411.2019 [M − H_2_O + H]^+^, (calcd for C_21_H_31_O_8_, 411.2024).

*Lobatin E* (**3**): an amorphous solid; [α]^27^_D_ −25 (*c* 0.2, CHCl_3_); ECD {CH_3_CN, *λ*_max_ [nm] (Δε)}, *c* 7.37 × 10 − 4 M: 330sh (+0.84), 319 (+1.11), 288 (−0.08), 279 (+0.13), 256 (−1.06), 233 (+1.61), 207 (−6.10); ^1^H NMR and ^13^C NMR data, see Table 1 and Table 2; HR-ESIMS *m*/*z* 377.1597 [M + H]^+^ (calcd for C_20_H_25_O_7_, 377.1600).

*Lobatin C* (**4**): an amorphous solid; [α]^27^_D_ −4 (*c* 0.1, CHCl_3_). ECD {CH_3_CN, *λ*max [nm] (Δε)}, *c* 2.75 × 10 − 4 M: 330sh (+0.80), 321 (+1.05), 288 (−0.14), 277 (+0.03), 256 (−0.49), 227 (+1.49), 204 (−4.48); ^1^H NMR and ^13^C NMR data, see Table 1 and Table 2; APCI-MS positive *m*/*z* 419 [M + H]^+^, 436 [M+NH_4_]^+^.

*Lobatolide B* (**5**): an amorphous solid; [α]^28^_D_ −69 (*c* 0.2, CHCl_3_); ECD {CH_3_CN, *λ*max [nm] (Δε)}, *c* 4.51 × 10 − 4 M: 302 (−0.75), 231 (−9.49), 199 (+2.19); ^1^H NMR and ^13^C NMR data, see Table 1 and Table 2; HR-ESIMS *m*/*z* 455.2274 [M + H]^+^ (calcd for C_23_H_35_O_9_ 455.2281).

*Lobatolide C* (**6**): a colorless gum; [α]^28^_D_ +34 (*c* 0.2, CHCl_3_); ECD {CH_3_CN, *λ*max [nm] (Δε)}, *c* 4.80 × 10 − 4 M: 259 (−1.15), 221sh (+2.91), 196 (+21.51); ^1^H NMR and ^13^C NMR data, see Table 1 and Table 2; HR-ESIMS *m*/*z* 407.2063 [M + H]^+^ (calcd for C_22_H_31_O_7_, 407.2070).

*Lobatolide D* (**7**): white amorphous powder; [α]^27^_D_ −30 (*c* 0.1, CHCl_3_); ECD {CH_3_CN, *λ*max [nm] (Δε)}, *c* 1.38 × 10 − 4 M: 328sh (+1.17), 315 (+1.99), 255sh (+5.76), 246 (+7.25), 217 (−27.65); ^1^H NMR and ^13^C NMR data, see Table 3; HR-ESIMS positive *m*/*z* 799.3551 [M + H]^+^ (calcd for C_42_H_55_O_15_, 799.3541); 821.3352 [M+Na]^+^ (calcd for C_42_H_54_O_15_Na, 821.3360).

*8β-Isovaleroyloxyreynosin* (**8**): a colorless oil; [α]^28^_D_ +8 (*c* 0.1, CHCl_3_); ECD {CH_3_OH, *λ*max [nm] (Δε)}, *c* 4.59 × 10 − 4 M: 248 (−0.32), 222 (+0.17); ^1^H NMR and ^13^C NMR data, see Table 3; HR-ESIMS *m*/*z* 349.2017 [M + H]^+^, (calcd for C_20_H_29_O_5_, 349.2015).

*Volenol* (**9**): ECD {CH_3_CN, *λ*max [nm] (Δε)}, *c* 3.52 × 10 − 4 M: 194 (−3.50).

### 2.5. Computational Section

Mixed torsional/low-mode conformational searches were carried out by means of the Macromodel 10.8.011 software using the MMFF with an implicit solvent model for CHCl_3_ applying a 21 kJ/mol energy window [24]. Geometry re-optimizations of the resultant conformers [CAM-B3LYP/TZVP PCM/MeCN, CAM-B3LYP/TZVP PCM/CHCl_3_, B3LYP/6-31+G(d,p) level in vacuo], TDDFT-ECD [B3LYP/TZVP PCM/MeCN, BH&HLYP/TZVP PCM/MeCN, CAM-B3LYP/TZVP PCM/MeCN and PBE0/TZVP PCM/MeCN], or [B3LYP/TZVP PCM/CHCl_3_, BH&HLYP/TZVP PCM/CHCl_3_, CAM-B3LYP/TZVP PCM/CHCl_3_ and PBE0/TZVP PCM/CHCl_3_] and DFT-NMR calculations [mPW1PW91/6-311+G(2d,p) and mPW1PW91/6-311+G(2d,p) SMD/CHCl_3_] were performed with the Gaussian 09 package [25]. ECD spectra were generated as sums of Gaussians with 2400–3600 cm^−1^ width at half-height, using dipole-velocity-computed rotational strength values [26]. Computed NMR shift data were corrected with I = 185.4855 and S = −1.0306 for the carbons and I = 31.8996 and S = −1.0734 for the hydrogens in the gas-phase and I = 186.5242 and S = −1.0533 for the carbons and I = 31.8018 and S = −1.0936 for the hydrogens in the SMD calculations [27,28]. Boltzmann distributions were estimated from the CAM-B3LYP and the B3LYP energies. Visualization of the results was performed by the MOLEKEL software package [29].

### 2.6. Antiproliferative MTT Assay

Antiproliferative effect of the isolated compounds were determined in vitro using SiHa (HPV 16+), HeLa (HPV 18+), C33A (HPV negative) human cervical cell lines, NIH-3T3 mouse embryonic and MRC-5 human fibroblast cells by means of MTT ([3-(4,5-dimethylthiazol-2-yl)-2,5-diphenyltetrazolium bromide]) assay. Briefly, a limited number of human cancer cells (5000/well for SiHa and HeLa cells, 10,000/well in case of C33A cells) were seeded onto a 96-well microplate and became attached to the bottom of the well overnight. On the second day of the procedure, the test substances were added in two concentrations (10.0 and 30.0 µM) in order to obtain preliminary data and then the compounds were applied in serial dilution (applied final concentrations were: 0.1, 0.3, 1.0, 3.0, 10.0, 30.0 µM). Further details of the experiment were described in ref. [30]. All in vitro experiments were carried out on two 96-well dishes with at least five parallel wells [31].

### 2.7. Cell Cycle Analysis by Flow Cytometry

Cell cycle analysis was performed as described in [32,33].

### 2.8. Wound Healing Assay

In order to assess antimetastatic activity of the tested compound, wound healing assay was performed. The assay was done with specific wound healing assay chambers (Ibidi GmbH, Gräfelfing, Germany). SiHa cells were collected and 35,000 cells were seeded into both chambers of the insert. Cells were let to be attached to the plate surface during an overnight incubation at 37 °C in 5% CO_2_ atmosphere and then the inserts were removed. Cell debris was removed by a washing step with PBS. Test compounds were added to the wells in increasing concentrations in 2% FBS containing medium for 24 and 48 h. Migration of the cells into the wound site was visualized by phase-contrast inverted microscope (Axiovert 40, Zeiss, Thornwood, NY, USA). Images were taken with CCD camera at definite intervals and the migration of the cells was calculated as the ratio of wound closure by ImageJ software [34].

### 2.9. Statistical Analysis

Statistical analysis of the obtained data was performed by analysis of variance (ANOVA) followed by Dunnett’s test. All analyses were performed with GraphPad Prism 5 (GraphPad Software; San Diego, CA, USA).

## 3. Results and Discussion

**Figure 1:**
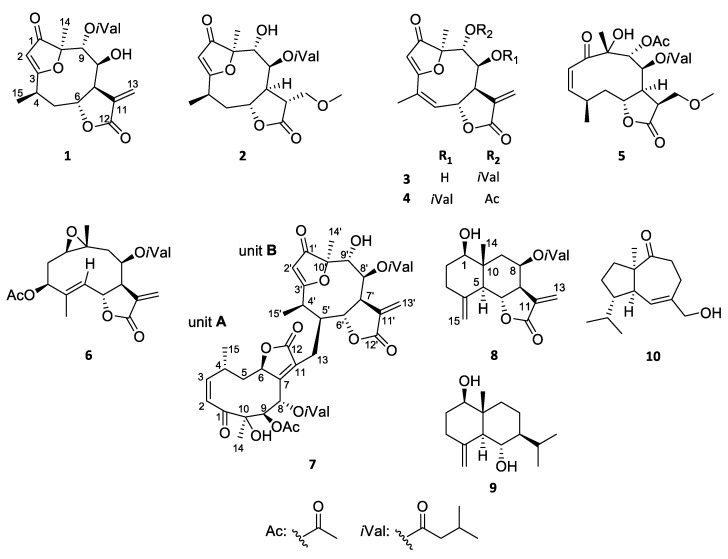
Structures of compounds **1**–**10** isolated from *N. lobata*.

Phytochemical investigation of the CH_2_Cl_2_-soluble phase of the MeOH extract, obtained from the aerial parts of *N. lobata*, resulted in the isolation of ten sesquiterpenes (**1**–**10**) (Figure 1). For the separation of the compounds a combination of different chromatographic methods, including open column chromatography (OCC), vacuum liquid chromatography (VLC), rotation planar chromatography (RPC), and preparative TLC were used. The structure elucidation was carried out by extensive spectroscopic analysis, using HR-ESIMS, and 1D and 2D NMR (^1^H–^1^H COSY, HSQC, HMBC and NOESY) spectroscopy and DFT-NMR calculations.

### 3.1. Structure Elucidation of the Isolated Compounds

#### 3.1.1. Lobatolide A (**1**)

Compound **1** was isolated as an amorphous solid with [α]^28^_D_ +12 (*c* 0.1, CHCl_3_). Its HR-ESIMS displayed a quasimolecular ion peak at *m*/*z* 379.1755 [M + H]^+^ (calcd for 379.1757), indicating the molecular formula, C_20_H_26_O_7_. The 1D and 2D NMR spectra exhibited resonances for a carbonyl group (*δ*_C_ 203.9), an *α*-methylene-*γ*-lactone ring [*δ*_H_ 4.78 dd (H-6), 3.30 dd (H-7), 6.30 d (H-13a) and 5.35 d (H-13b), *δ*_C_ 75.3 (C-6), 47.9 (C-7), 141.3 (C-11), 169.2 (C-12) and 122.6 (C-13)], a quaternary carbon (*δ*_C_ 88.9), a trisubstituted olefin (*δ*_H_ 5.60 d, *δ*_C_ 104.0 and 192.8), three methines (*δ*_H_ 3.01 m, 4.13 t and 5.27 d, *δ*_C_ 31.5, 73.8 and 77.1), and one secondary (*δ*_H_ 1.40 d, *δ*_C_ 16.1) and one tertiary methyl group (*δ*_H_ 1.37 s, *δ*_C_ 18.9) (Table 1 and Table 2). Further ^1^H and ^13^C NMR signals at *δ*_H_ 2.38 dd, 2.33 m, 2.19 m, 2 × 1.02 d, and *δ*_C_ 171.5, 43.1, 25.7, 2 × 22.4, were attributed to an isovaleroyloxy group. The chemical shifts and coupling constants of **1** were closely related to those of the 1-keto-furanoheliangolide derivative 8-isovaleroyloxy-9α-hydroxy-calyculatolide [9], with the only difference changing of the positions of isovaleroyloxy and hydroxy groups. The isovaleroyl group was assigned to C-9 with regard to the HMBC correlations between H-9 and the carbonyl carbon signal of isovaleroyl group. The relative configuration of compound **1** was determined by means of a NOESY experiment. The coupling constant of H-6 and H-7 (*J*_6,7_ = 5.0 Hz) indicated the *β* orientation of H-6 and *α* orientation of H-7, found in all sesquiterpenes isolated previously from this plant. NOESY correlation between H-6 and H-15 confirmed the *β* position of the 15-methyl group, while NOE effects observed between H-7 and H-5a, H-5a and H-4, H-7 and H-13b, and H-13b and H-8 dictated the α orientation of these protons. All of the above evidence supported the structure of this compound as 8*β*-hydroxy-9*α*-isovaleroyloxycalyculatolide, and named as lobatolide A (**1**).

**Figure 2:**
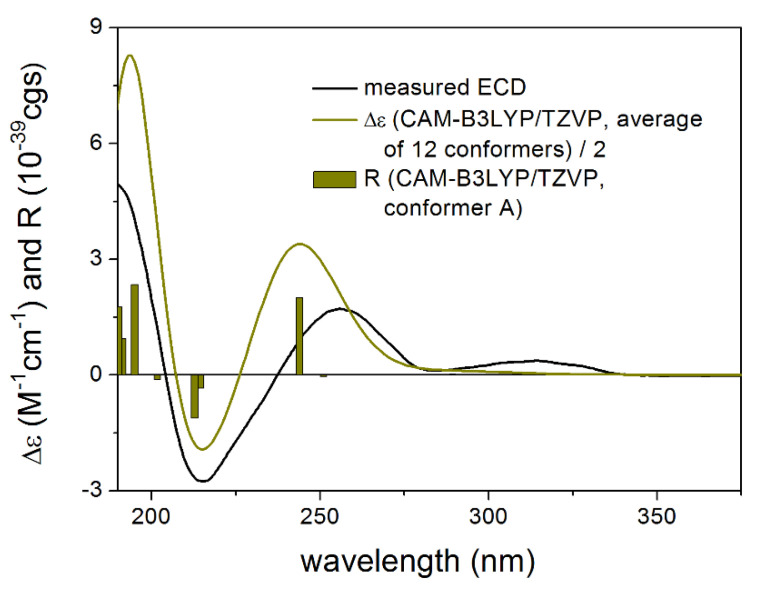
Comparison of the experimental ECD spectrum of **1** measured in MeCN with the CAM-B3LYP/TZVP PCM/MeCN spectrum of (4*R*,6*R*,7*R*,8*S*,9*R*,10*R*)-**1** (level of optimization: CAM-B3LYP/TZVP PCM/MeCN). The bars represent the rotational strength values of the lowest energy conformer.

**Figure 3:**
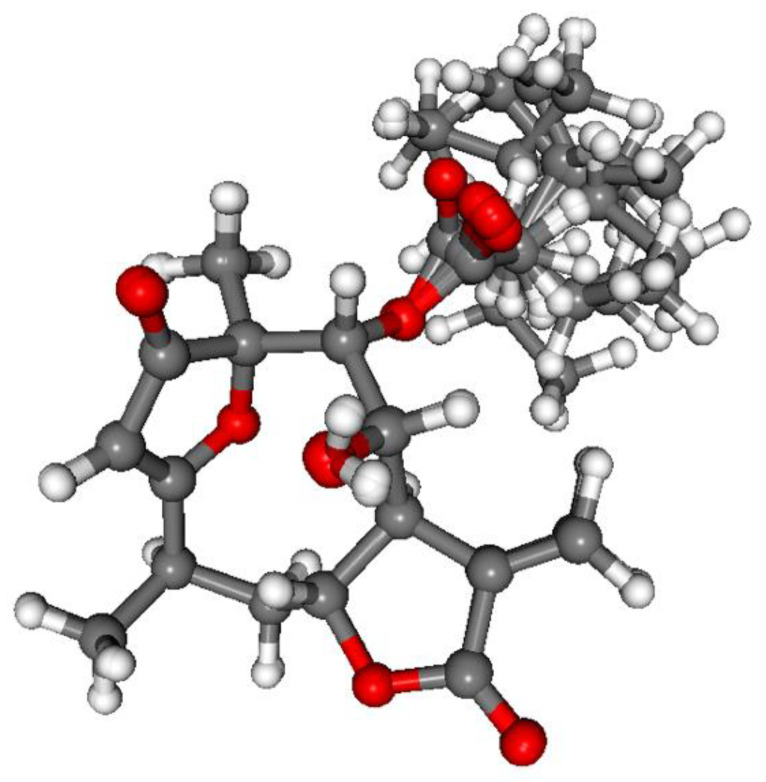
Overlapped geometries of the twelve low-energy CAM-B3LYP/TZVP PCM/MeCN conformers of (4*R*,6*R*,7*R*,8*S*,9*R*,10*R*)-**1**.

The absolute configuration of **1** was determined by the solution TDDFT-ECD method [35,36]. Merck Molecular Force Field (MMFF) conformational search of (4*R*,6*R*,7*R*,8*S*,9*R*,10*R*)-**1** resulted in 133 initial conformers in a 21 kJ/mol energy window. These structures were re-optimized at the CAM-B3LYP/TZVP [37] PCM/MeCN levels yielding 12 low-energy conformers over 1% Boltzmann population. ECD spectra computed at various levels of theory for these conformers reproduced well the experimental ECD spectrum (Figure 2). Furthermore, the low-energy conformers, differing only in the orientation of the *Oi*Val and the OH groups (Figure 3), gave similar computed ECD spectra allowing the solid elucidation of the absolute configuration as (4*R*,6*R*,7*R*,8*S*,9*R*,10*R*)-**1**. To test the applicability of the DFT-NMR method [28,38], the 133 MMFF conformers were also re-optimized at the B3LYP/6-31+G(d,p) level yielding seven low-energy conformers. The NMR shift values were computed for these conformers both at the mPW1PW91/6-311+G(2d,p) and the mPW1PW91/6-311+G(2d,p) SMD/CDCl_3_ levels [39]. Computed and experimental ^13^C NMR chemical shift values gave a good overall agreement with CMAE (corrected mean absolute error) values [40] of 1.92 and 1.77 ppm, respectively (Table S1). Although the ^13^C NMR DFT calculation showed good agreement for compound **1**, the exomethylene group caused larger deviations in the computed NMR shift values [41,42] of the other derivatives of this work even by testing at different levels of theory.

#### 3.1.2. Lobatolide B (**2**)

Compound **2** (lobatolide B) was isolated as amorphous solid with [α]^27^_D_ +47 (*c* 0.05, CHCl_3_). The molecular ion peak at *m*/*z* 411.2019 [M + H]^+^ (calcd for 411.2024) in the positive-ion HR-ESIMS showed its molecular formula to be C_21_H_30_O_8_. The ^1^H NMR spectrum displayed the presence of signals of an isovaleroyl side-chain (*δ*_H_ 2.14 dd, 2.10 brd, 2.02 m, 0.93 d, 0.91 d), and a methoxy signal (*δ*_H_ 3.40 s) (Table 1). From the ^1^H–^1^H COSY spectrum, one structural fragment with correlated protons was assigned: −CH(CH_3_)-CH_2_-CHR-CHR-CHR-CHR– (*δ*_H_ 3.01, 1.34, 2.63, 2.03, 4.29, 3.16, 5.07 and 3.94) (C-4–C-9). Long-range heteronuclear NMR correlations between the quaternary carbon C-3 and H-2, H-4, H-5b, and H_3_-15, between C-10 and H-2, H-8, and H_3_-14 signals, and between C-1 and H-2 and H-14 were observed in the HMBC spectrum. The two- and three-bond correlations revealed that the structural fragment together with C-1, C-3, C-10, the C-2 methine, and the 14-methyl group forms a furanoheliangolide skeleton. Detailed analysis of the spectral data suggested that compound **2** is very similar to 8*β*-isovalerianoyloxy-9*α*-hydroxycalyculatolide. The difference between the two compounds was the modified *α*-methylene-*γ*-lactone moiety. The upfield-shifted chemical shifts of H_2_-13 protons (*δ*_H_ 3.74 and 3.67) indicated an oxymethylene part in the structure instead of the exomethylene unit. Moreover, the presence of *sp*^3^ methine (*δ*_H_-11 2.89, *δ*_C_-11 49.3) resonances were observed. These observations led to the conclusion, that the C-11/C-13 double bond was saturated. ^1^H–^1^H COSY correlations between H-7 and H-11, H-11-and H-13, and long-range correlation between OMe and H_2_-13 proved that a methoxy group is connected at C-13.

**Figure 4:**
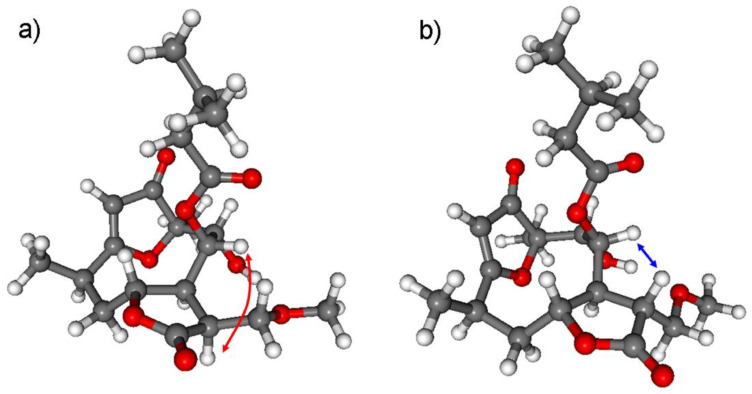
The characteristic H-8–H-11 NOE correlation indicated on the lowest-energy conformers of the (**a**) (4*R*,6*R*,7*S*,8*S*,9*R*,10*R*,11*S*)-**2** (3.50 Å) and (**b**) (4*R*,6*R*,7*S*,8*S*,9*R*,10*R*,11*R*)-**2** (2.19 Å) stereoisomers. Level of optimization: B3LYP/6-31+G(d,p).

The relative configuration of **2** corresponded well with the data reported for furanoheliangolides in the literature, containing an exomethylene moiety at the C-11 position [12]. The NOE correlations between H-8 and H-11 suggested *β* orientation of the methoxymethylene group at C-11. NOE correlations in a conformationally flexible macrolide ring have to be treated with precaution, since the large flexibility of the ring can lead to wrong assignment as reported in the literature for other related derivatives [43,44]. Thus the relative configuration of **2** was investigated by DFT-^13^C NMR calculations of eight selected stereoisomers with different configuration at the C-8, C-9 and C-11 chirality centers. The (4*R*,6*R*,7*S*,8*S*,9*R*,10*R*,11*S*), (4*R*,6*R*,7*S*,8*S*,9*R*,10*R*,11*R*), (4*R*,6*R*,7*S*,8*R*,9*R*,10*R*,11*S*), (4*R*,6*R*,7*S*,8*R*,9*R*,10*R*,11*R*), (4*R*,6*R*,7*S*,8*R*,9*S*,10*R*,11*S*), (4*R*,6*R*,7*S*,8*R*,9*S*,10*R*,11*R*), (4*R*,6*R*,7*S*,8*S*,9*S*,10*R*,11*S*) and (4*R*,6*R*,7*S*,8*S*,9*S*,10*R*,11*R*) stereoisomers of **2** were investigated at the mPW1PW91/6-311+G(2d,p)//B3LYP/6-31+G(d,p) and mPW1PW91/6-311+G(2d,p) SMD/CDCl_3_//B3LYP/6-31+G(d,p) levels, which were already tested for compound **1**. The NMR calculations showed that the H-8/H-11 NOE correlation is also feasible with *trans* relative configuration of H-8 and H-11 due to the flexibility of the macrolide ring. Moreover, this NOE effect is much more probable with the *β* orientation of H-11 hydrogen than with the *α* one. The H-8–H-11 interatomic distance in the lowest-energy conformer of (4*R*,6*R*,7*S*,8*S*,9*R*,10*R*,11*S*)-**2** (with *α* orientation of H-11) is 3.50, while this value is 2.19 Å for the (4*R*,6*R*,7*S*,8*S*,9*R*,10*R*,11*R*)-**2** stereoisomer (with *β* orientation of H-11) (Figure 4). Thus, the ^13^C NMR calculations revealed that the H-8/H-11 NOE correlation unexpectedly derives from the *trans* relative configuration of the H-8 and H-11 protons.

The computed ^13^C NMR data showed clear preference for the (4*R*,6*R*,7*S*,8*S*,9*R*,10*R*,11*R*)-**2** stereoisomer with a CMAE value of 1.61 vs. 2.36, 2.66, 2.51, 2.11, 2.22, 2.44 and 2.25 in the gas-phase calculations and 1.58 vs. 2.36, 2.71, 2.32, 2.27, 2.30, 2.37 and 2.23 in the SMD calculations (Tables S2 and S3). The DP4+ statistical analysis gave a 99.98% confidence in the gas-phase and 100.00% with SMD solvent model for this isomer [45,46]. The data above corroborate the proposed molecular formula of lobatolide B (**2**). The ECD spectrum of **2** was recorded both in MeCN and MeOH with different settings but nearly baseline ECD spectra were recorded most likely due to solubility problems. Since +47 value was measured for the specific rotation, this was used instead of the ECD to determine the absolute configuration [47]. CAM-B3LYP/TZVP PCM/CHCl_3_ re-optimization of the 136 MMFF conformers resulted in 17 low-energy conformers. OR values were computed for all the conformers at four different levels of theory and they had positive value in all the combinations and the Boltzmann weights were in the range of +53–+71 showing good agreement with the +47 experimental value (Table S4). Thus the absolute configuration of **2** was assigned as (4*R*,6*R*,7*S*,8*S*,9*R*,10*R*,11*R*) in line with the biosynthetic considerations.

#### 3.1.3. Lobatolide C (**3**)

Compound **3** was isolated as an amorphous solid with [α]^27^_D_ −25 (*c* 0.2, CHCl_3_). HR-ESIMS and detailed 1D and 2D NMR spectroscopic studies led to a furanoheliangolide structure for compound **3**, found previously in case of lobatin B [10], on the basis of the following arguments. HR-ESIMS suggested that the molecular composition of the compound is C_20_H_24_O_7_, according to the quasimolecular ion peak at 377.1597 [M + H]^+^ (calcd for 377.1600). Analysis of the ^1^H and ^13^C NMR spectra confirmed that lobatin B and compound **3** are structurally related. The ^1^H and ^13^C NMR spectra revealed the presence of an isovaleroyl group (*δ*_H_ 2.37 dd, 2.31 dd, 2.16 m, 2 × 1.01 d; *δ*_C_ 171.7, 43.1, 25.6 and 2 × 22.4) and two quaternary carbon-bonded methyl groups (*δ*_H_ 1.41 s, 2.06 s, δ_C_.18.4, 19.6) in the molecule (Table 1 and Table 2). Informative signals at *δ*_H_ 5.71 m (H-6), 3.57 dd (H-7), 6.29 d (H-13a), and 5.35 d (H-13b) and *δ*_C_ 74.9 (C-6), 45.2 (C-7), 141.2 (C-11), 170.0 (C-12), and 123.2 (C-13) additionally indicated the presence of a *trans*-fused *α*-methylene-*γ*-lactone ring with H-6 in the *β* position. The isovaleroyl group was assigned to C-9 with regard to the HMBC correlations between H-9 and the carbonyl carbon signal (isovaleroyl CO). The only difference between compound **2** and lobatin B is the connection of isovaleroyl (at C-9 in **3**, and at C-8 in lobatin B), and hydroxy (at C-8 in **3**, and at C-9 in lobatin B) groups.

The relative configuration of compound **3** was studied by means of a NOESY experiment. Cross-peak between H-5 and H_3_-15, and the small coupling constant (*J*_5_ = 1.8 Hz) detected at H-5 proved the *Z*-configuration of the double bond (C-4–C-5). NOE effects observed between H-7 and H-13b, and H-13b and H-8 dictated the *α* orientation of these protons. All of the above evidence supported the structure of **3** as 8*β*-hydroxy-9*α*-isovaleroyloxyatripliciolide, which was named as lobatolide C.

**Figure 5:**
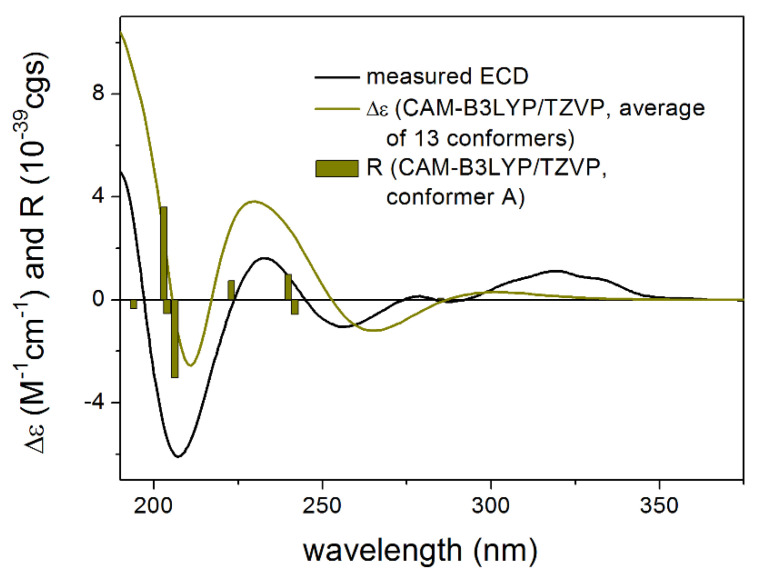
Comparison of the experimental ECD spectrum of **3** measured in MeCN with the CAM-B3LYP/TZVP PCM/MeCN spectrum of (6*R*,7*R*,8*S*,9*R*,10*R*)-**3** (level of optimization: CAM-B3LYP/TZVP PCM/MeCN). The bars represent the rotational strength values of the lowest-energy conformer.

**Figure 6:**
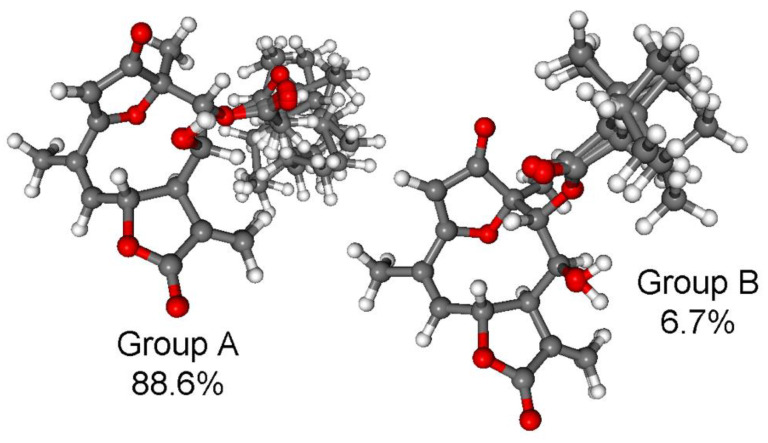
Overlapped geometries of the conformers of the two conformer groups of (6*R*,7*R*,8*S*,9*R*,10*R*)-**3**. Group A: Confs. A-F, I, J; group B: Confs. G, H, K-M. Level of optimization: CAM-B3LYP/TZVP PCM/MeCN.

**Figure 7:**
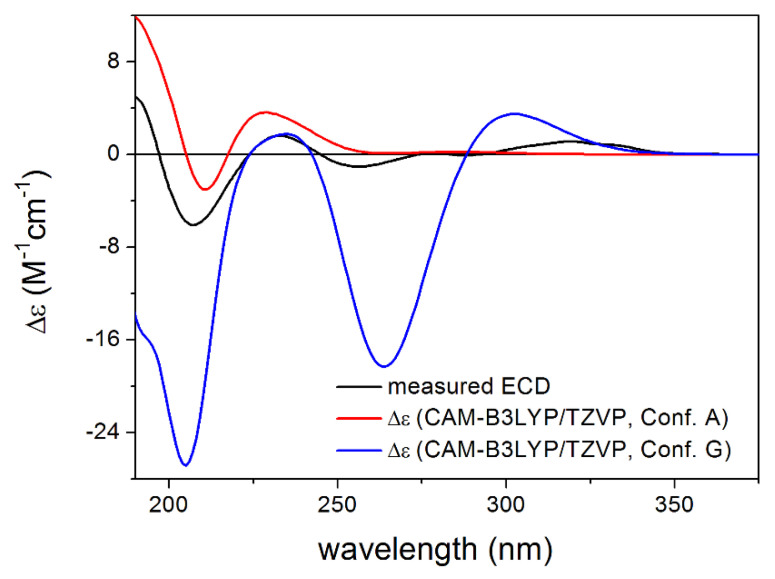
Comparison of the experimental ECD spectrum of **3** measured in MeCN with the CAM-B3LYP/TZVP PCM/MeCN spectra of conformers A and G of (6*R*,7*R*,8*S*,9*R*,10*R*)-**3**, as the lowest-energy representatives of groups A and B. Level of optimization: CAM-B3LYP/TZVP PCM/MeCN.

CAM-B3LYP PCM/MeCN re-optimization of the initial 92 MMFF conformers yielded 13 low-energy conformers, which can be sorted into two groups based on the axial or equatorial arrangement of the C-9 *Oi*Val substituent (Figure 5). The conformers of a group showed difference in the rotation of the C-9 *Oi*Val group (Figure 6). The conformers of groups A and B had markedly different computed ECD spectra, while conformers in the same group had similar curves. Although group B conformers had low populations, they had more intense computed ECD spectra than those of group A, and they governed the feature of the sum ECD curve above 240 nm (Figure 7). The Boltzmann-weighted ECD spectra reproduced well the major transitions of the experimental ECD spectrum allowing elucidation of the absolute configuration as (6*R*,7*R*,8*S*,9*R*,10*R*), which is in accordance with the biosynthetic considerations.

#### 3.1.4. Lobatin C (**4**)

**Figure 8:**
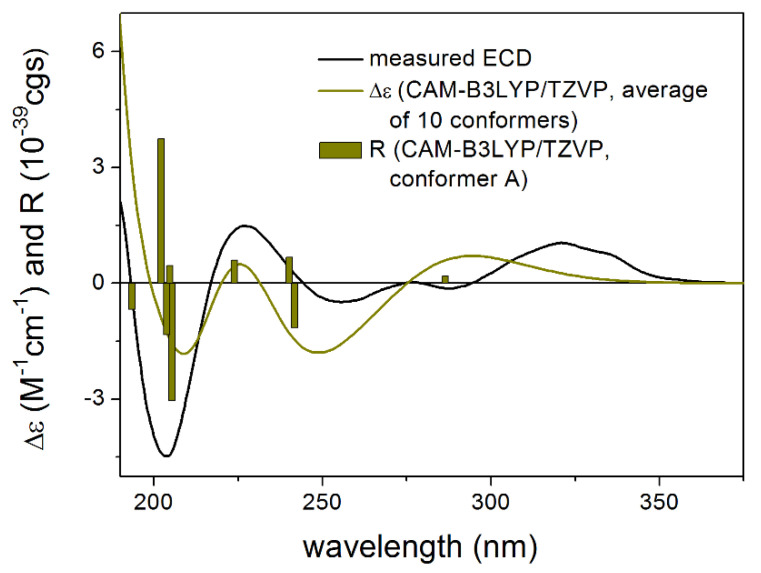
Comparison of the experimental ECD spectrum of **4** measured in MeCN with the CAM-B3LYP/TZVP PCM/MeCN spectrum of (6*R*,7*S*,8*S*,9*R*,10*R*)-**4** (level of optimization: CAM-B3LYP/TZVP PCM/MeCN). The bars represent the rotational strength values of the lowest energy conformer.

Compound **4** was isolated as an amorphous solid with [α]^27^_D_ −4 (*c* 0.1, CHCl_3_). Based on 1D and 2D NMR data it was determined as lobatin C, isolated previously from *N. lobata* and *N. macrocephala* by Passreiter et al. [9,48]. As the compound was identified only by GC-MS analysis [9], and no NMR data were published previously in the literature, detailed NMR studies were performed affording the ^1^H and ^13^C NMR assignments (Table 1 and Table 2). CAM-B3LYP/TZVP PCM/MeCN re-optimization of the initial 109 MMFF conformers of (6*R*,7*S*,8*S*,9*R*,10*R*)-**4** yielded 10 low-energy conformers and similarly to **3**, two conformer groups could be identified with different conformations of the macrolide ring. In group A representing 90.2% sum population, the C-8 *Oi*Val substituent had axial orientation, while it was equatorial in group B conformers (5.6% sum population). The Boltzmann-weighted CAM-B3LYP/TZVP PCM/MeCN ECD spectra reproduced well all the major transitions of the experimental ECD spectrum and hence the absolute configuration was determined as (6*R*,7*S*,8*S*,9*R*,10*R*) (Figure 8, Figures S21 and S22).

#### 3.1.5. Lobatolide D (**5**)

Compound **5** was isolated as an amorphous solid with [α]^28^_D_ −69 (*c* 0.2, CHCl_3_). The molecular formula C_23_H_34_O_9_ was determined by HR-ESIMS with a molecular ion peak at *m*/*z* 455.2274 [M + H]^+^ (calcd for C_23_H_35_O_9_ 455.2281). Detailed analysis of spectral data afforded the structure, which was in good agreement with neurolenin B. The missing exomethylene signals (*δ*_H_ 6.31 and 5.81, br s each), as well as additional *sp*^3^ methine at *δ*_H_ 3.04 m, *δ*_C_ 40.2 (C-11) and oxymethylene resonances detected at *δ*_H_ 3.34 t and 3.68 dd and *δ*_C_ 66.3 (C-13), indicated the saturation of the C-11/C-13 double bond (Table 1). The H-7/H-11/H-13 spin system was established by means of ^1^H–^1^H COSY correlations and HMBC cross peaks of H-7/C-11, H_2_-13/C-7 and H_2_-13/C-11. The ^1^H-spectrum contained an additional methoxy signal (*δ*_H_ 3.39 s), which exhibited HMBC correlation with *δ*_C_ 66.3 and, therefore, must be situated at C-13. The relative configuration of compound **5**, named as lobatolide D, was found to be identical with that of neurolenin B. The presence of *trans*-fused lactone-ring was demonstrated by NOE-effects of H-5a/H-6, H-5b/H-7, H-5b/H-4. The α position of H-11 was suggested by the NOE correlations H-11/H-7 and H-11/H-4, which implies a different relative configuration of the C-8, C-9, C-11 centers from that of compound **2**. In order to assist the assignment of the relative configuration, DFT-^13^C NMR calculations were performed on eight stereoisomers differing in the C-8, C-9, C-11 chirality centers. The (4*R*,6*R*,7*S*,8*S*,9*R*,10*R*,11*S*), (4*R*,6*R*,7*S*,8*S*,9*R*,10*R*,11*R*), (4*R*,6*R*,7*S*,8*R*,9*R*,10*R*,11*S*), (4*R*,6*R*,7*S*,8*R*,9*R*,10*R*,11*R*), (4*R*,6*R*,7*S*,8*R*,9*S*,10*R*,11*S*), (4*R*,6*R*,7*S*,8*R*,9*S*,10*R*,11*R*), (4*R*,6*R*,7*S*,8*S*,9*S*,10*R*,11*S*) and (4*R*,6*R*,7*S*,8*S*,9*S*,10*R*,11*R*) stereoisomers of **5** were computed at the mPW1PW91/6-311+G(2d,p)//B3LYP/6-31+G(d,p) and mPW1PW91/6-311+G(2d,p) SMD/CDCl_3_//B3LYP/6-31+G(d,p) levels. The lowest-energy conformers of (4*R*,6*R*,7*S*,8*S*,9*R*,10*R*,11*S*)-**5** and (4*R*,6*R*,7*S*,8*S*,9*R*,10*R*,11*R*)-**5** differing in the configuration of the C-11 center showed that the H-11/H-7 NOE correlation is feasible for both of them with 2.33 Å interatomic distance for the (11*S*) and 2.86 Å for the (11*R*) epimer. The H-11/H-4 distance is found shorter (3.47 Å) for the (11*S*) epimer [4.96 Å in the (11*R*) epimer]. The computed ^13^C NMR chemical shift data did not show a clear preference for any stereoisomers, and thus the ^1^H NMR data were taken also into account (Tables S5–S8). The DP4+ statistical analysis of the combined ^13^C and ^1^H NMR data gave 99.37% confidence with the SMD solvent model for the (4*R*,6*R*,7*S*,8*S*,9*R*,10*R*,11*S*)-**5** stereoisomer. Interestingly, the (4*R*,6*R*,7*S*,8*S*,9*R*,10*R*,11*R*) stereoisomer of **5**, which is homochiral with **2**, gave 0.00% probability at both NMR levels. This result suggested that C-11 chirality center of **2** and **5** was produced by the reduction of the exomethylene moiety, which afforded different C-11 configurations in compounds **2** and **5**.

**Figure 9:**
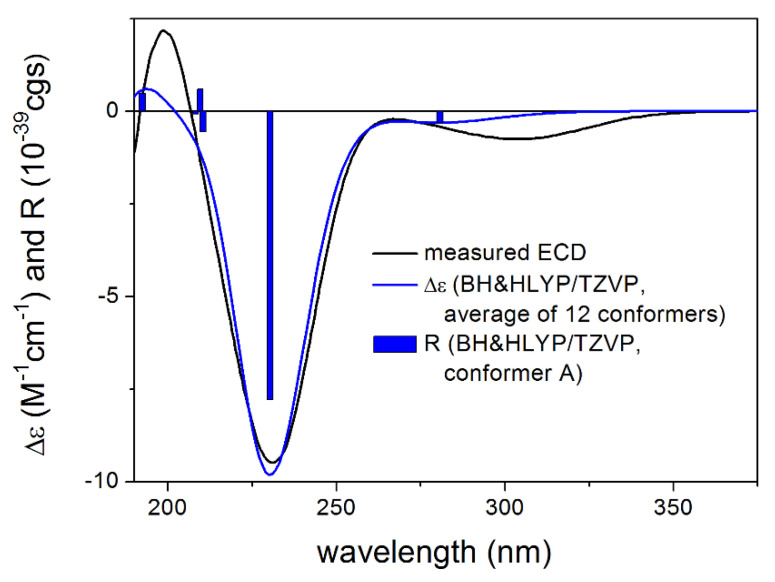
Comparison of the experimental ECD spectrum of **5** measured in MeCN with the BH&HLYP/TZVP PCM/MeCN spectrum of (4*R*,6*R*,7*S*,8*S*,9*R*,10*R*,11*S*)-**5** (level of optimization: CAM-B3LYP/TZVP PCM/MeCN). The bars represent the rotational strength values of the lowest energy conformer.

**Figure 10:**
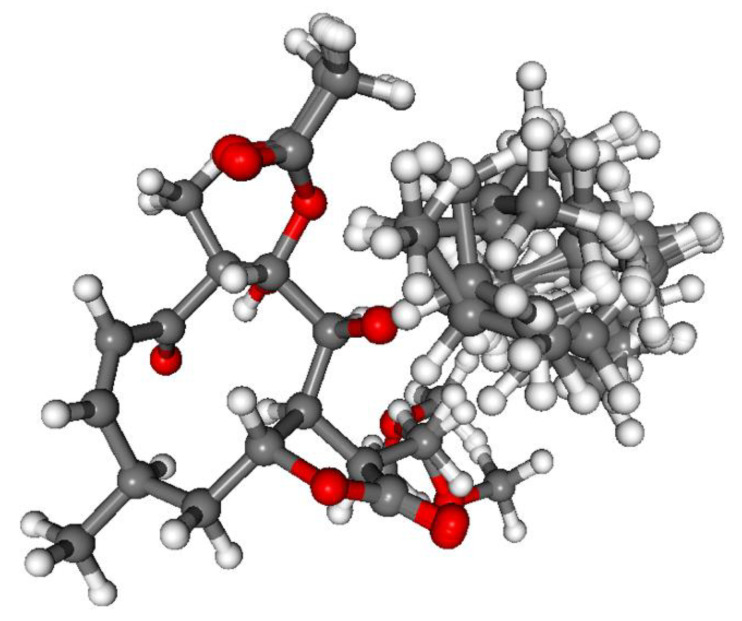
Overlapped geometries of the twelve low-energy CAM-B3LYP/TZVP PCM/MeCN conformers of (4*R*,6*R*,7*S*,8*S*,9*R*,10*R*,11*S*)-**5**.

In the knowledge of the relative configuration, the absolute configuration was determined by the TDDFT-ECD protocol performed on the (4*R*,6*R*,7*S*,8*S*,9*R*,10*R*,11*S*)-**5** stereoisomer (Figure 9). CAM-B3LYP re-optimization of the initial 89 MMFF conformers resulted in 12 low-energy conformers, which gave quite similar ECD spectra (Figure 10). The Boltzmann-weighted ECD spectra gave a good agreement with the major transitions of the experimental ECD spectrum at all the applied levels of theory allowing elucidation of the absolute configuration as (4*R*,6*R*,7*S*,8*S*,9*R*,10*R*,11*S*)-**5**.

#### 3.1.6. Lobatolide E (**6**)

Compound **6** was obtained as a colorless gum with [α]^28^_D_ +34 (*c* 0.2, CHCl_3_). From the molecular ion peak at *m*/*z* 407.2063 [M + H]^+^ (calcd for 407.2070) in the positive-mode HR-ESIMS, its molecular formula was determined to be C_22_H_30_O_7_. The ^1^H NMR spectrum showed the presence of signals due to an isovaleroyl and an acetyl group (Table 1). Further, the ^13^C-JMOD spectrum suggested that the skeleton consists of fifteen carbons (Table 2). From the ^1^H–^1^H COSY spectrum two structural fragments were assigned on the basis of correlated protons: −CHR-CH_2_-CH– (A) (*δ*_H_ 2.93, 2.35, 1.64 and 5.50) (C-1–C-3) and =CH-CHR-CHR-CHR-CH_2_– (B) (*δ*_H_ 5.66, 5.22, 3.21, 5.74, 2.63 and 1.40) (C-5–C-9). These structural elements, tertiary methyls and quaternary carbons were connected by examination of the long-range C–H correlations detected in the HMBC spectrum. The two- and three-bond correlations between H-3, H-6, H-15, H_2_-2 and the quaternary carbon C-4, and between H_2_-2, H-8, H_2_-9, H-14 and the quaternary C-10 and signals revealed that structural fragment A together with C-10, C-4, and the 14- and 15-methyl groups forms a germacrane skeleton. The lactone ring connected to the macrocycle in position C-6, C-7 was evident from the HMBC cross-peaks between H_2_-13, and C-12, and H_2_-13, H-7 and C-11. The position of the ester groups was proved by the long-range correlation between the ester carbonyl of isovaleroyl group (*δ*_C_ 173.2) and H-8 (*δ*_H_ 5.74 d) and the acetyl carbonyl (*δ*_C_ 171.8) and H-3 (*δ*_H_ 5.50 d). The remaining epoxy group, which were elucidated from the molecular composition, was placed at C-10–C-1, with regard to the ^13^C NMR chemical shifts (*δ*_C-1_ 65.6 and *δ*_C-10_ 61.4) and literature data for similar epoxy germacranolides [49]. The relative configuration of the chiral centers was studied by NOESY measurements. Diagnostic NOE correlations were detected between H-6 and H_3_-14, demonstrating the *β* orientation of these protons. Furthermore, NOESY cross-peaks were observed between H-1/H-2a, H-1/H-3, H-2a/H-3, H-1/H-9b, H-9b/H-7, H-7/H-8, H-14/H-9a, H-14/H-2b, H-6/H-14, indicating the *α*-oriented H-1, H-2a, H-7, H-8 and H-9b and the *β*-orientation of H-6, H-9a, H-2b and H-14. All of the above evidence was used to propose the structure of this compound as depicted in structural formula **6**. Compound **6** is the 8-epimer of 8*α*-isovaleryloxy-8-desacylviguestenin, reported earlier from *Viguiera procumbens* (Asteraceae) [49] Moreover, in contrast to 8*α*-isovaleryloxy-8-desacylviguestenin, the *trans* position of double bond between C-4–C-5 was proved by the NOE correlations between H-6 and H_3_-15 (above the plane of the macrocycle) and between H-3 and H-5 (below the plane of the ring). Therefore, lobatolide E was elucidated as depicted structural formula **6**.

**Figure 11:**
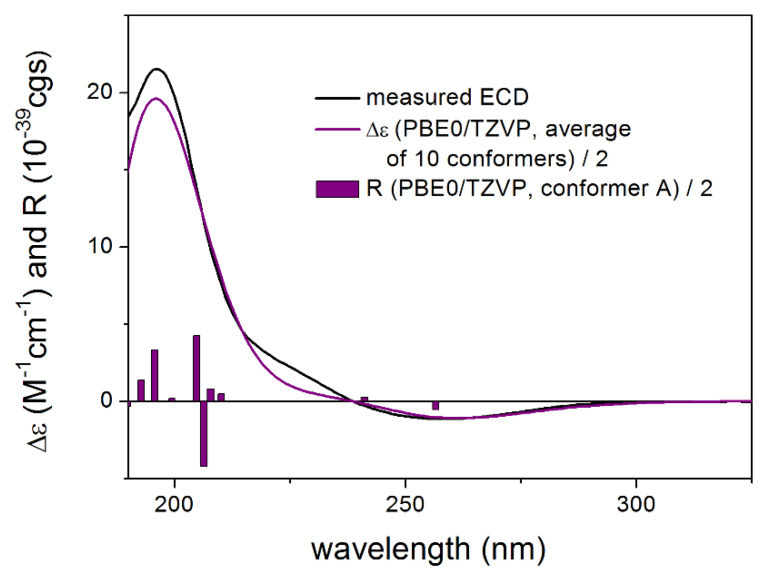
Comparison of the experimental ECD spectrum of **6** measured in MeCN with the PBE0/TZVP PCM/MeCN spectrum of (1*R*,3*S*,6*R*,7*R*,8*R*,10*R*)-**6** (level of optimization: CAM-B3LYP/TZVP PCM/MeCN). The bars represent the rotational strength values of the lowest energy conformer.

**Figure 12:**
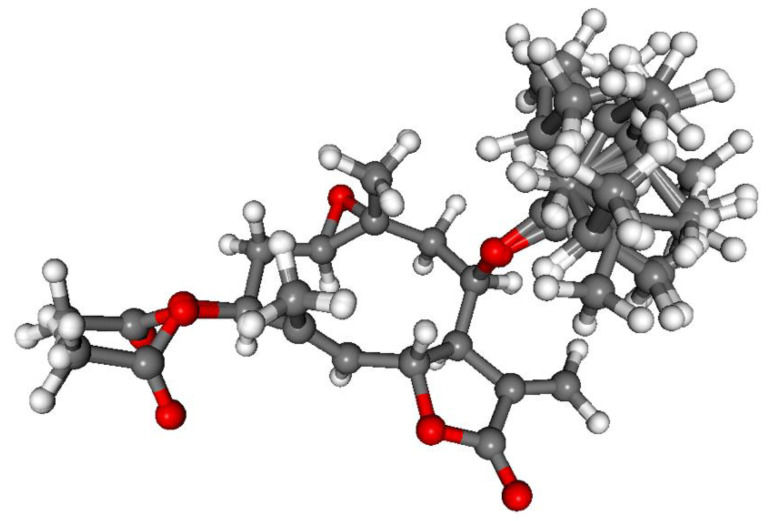
Overlapped geometries of the ten low-energy CAM-B3LYP/TZVP PCM/MeCN conformers of (1*R*,3*S*,6*R*,7*R*,8*R*,10*R*)-**6**.

ECD calculations performed for the 10 low-energy CAM-B3LYP conformers of (1*R*,3*S*,6*R*,7*R*,8*R*,10*R*)-**6** obtained from the re-optimization of the initial 74 MMFF conformers gave nice overall agreement with the experimental ECD spectrum (Figure 11). In all the 10 computed conformers, the C-8 *Oi*Val substituent adopted axial orientation and the conformers gave very similar ECD spectra allowing elucidation of the absolute configuration of **6** as (1*R*,3*S*,6*R*,7*R*,8*R*,10*R*) (Figure 12).

#### 3.1.7. Lobatolide F (**7**)

Compound **7** was obtained as a white amorphous powder with [α]^27^_D_ −30 (*c* 0.1, CHCl_3_). The molecular formula was established as C_42_H_54_O_15_ from its HR-ESIMS, which showed the quasimolecular ion peak at [M + H]^+^ 799.3551 (calcd for C_42_H_55_O_15_, 799.3541), requiring 16 degrees of unsaturation. The JMOD experiment displayed 42 resonances, including seven carbonyl, eight olefinic, nine methyl, four *sp*^3^ methylene, twelve *sp*^3^ methine (six of which oxymethine) and two oxygen-bearing *sp*^3^ quaternary carbons (Table 3). The combination of ^1^H and ^1^H–^1^H COSY spectra, revealed the presence of one *cis*-double-bond [H-2 (*δ*_H_ 6.22 d, *J* = 11.6 Hz), H-3 (*δ*_H_ 6.04 t, *J* = 11.6 Hz)], an isolated [H-2′ (*δ*_H_ 5.64, s)] and two exocyclic olefinic protons [H-13a’ (*δ*_H_ 6.29 d, *J* = 3.0 Hz); H-13b’ (*δ*_H_ 5.74 d, *J* = 3.0 Hz)], which are characteristic parts of the *α*-methylene-*γ*-lactone moiety (Table 3). Three ester residues were also identified as an acetyl [(*δ*_H_ 2.15 s (3H)] and two isovaleroyl-groups [H-2a′ (*δ*_H_ 2.27 dd, *J* = 15.8, 6.8 Hz); H-2b′ (*δ*_H_ 2.15, m); H-3′ (*δ*_H_ 2.02 m); H-4′ (*δ*_H_ 0.94 d, (3H) *J* = 6.7 Hz); H-5′ (*δ*_H_ 0.93 d, (3H), *J* = 6.7 Hz)], and [H-2″ (*δ*_H_ 2.08 m, (2H)]; H-3″ (*δ*_H_ 1.96 m); H-4″ (*δ*_H_ 0.90 d, (3H), *J* = 6.7 Hz); H-5″ (*δ*_H_ 0.87 d, (3H), *J* = 6.7 Hz)]. Comprehensive analysis of the 1D and 2D spectra led to the assignments of two different germacranolide units, designated as A and B.

Comparison of the NMR data of part A with those of sesquiterpenes previously isolated from the plant showed great similarities of unit A with neurolenin B. However, the absence of exomethylene and H-7 signals, as well as the appearance of proton resonances at *δ*_H_ 3.05 (dd, *J* = 14.5, 8.8 Hz) and *δ*_H_ 2.48 (dd, *J* = 14.5, 6.8) attributed to a saturated methylene suggested the relocation of the double bond from C-11–C-13 to C-7–C-11. This conclusion was supported by the significantly downfield-shifted signal of H-8 (*δ*_H_ 6.38) (neurolenin B: *δ*_H_ 5.56), in addition with HMBC correlations observed between the quaternary olefinic C-7 (*δ*_C_ 154.2) and H-6, H-8 and H-13a. Careful interpretation of the spectral data allowed determining part B as a 8*β*-isovaleroyloxy-9*α*-hydroxycalyculatolide unit. Chemical shifts of H-5′ (*δ*_H_ 3.54 m, 1H) and C-5 (*δ*_C_ 45.6) clearly indicated that part B is connected to A in position C-5. In addition, with the ^1^H–^1^H COSY correlations between H-13a/H-5′ and H-13b/H-5′ unambiguously established the linkage of units A and B through C-13/C-5′. HMBC cross-peaks of H-13a/C-4′, H-13a/C-5′ and H-13b/C-5′ provided further evidence of the connectivity.

The relative configuration of subunits corresponded well with the data reported for the monomers in the literature. The NOE correlations of H-4′/H-5′ and H-5′/H-7′ clearly indicated the *α*-orientation of H-5′. Overhauser effects between H-13a/H-8, H-13a/H-5′, H-6′/H-15′ and H-13b/H-15′ are also in agreement with the proposed molecular formula of **7**, named as lobatolide D.

#### 3.1.8. Lobatolide G (**8**)

Compound **8** was isolated as a colorless oil with [α]^28^_D_ +8 (*c* 0.1, CHCl_3_). Its HR-ESIMS spectrum showed quasimolecular ion at *m*/*z* 349.2017 [M + H]^+^, (calcd for 349.2015), indicating the molecular formula of C_20_H_28_O_5_. In the ^1^H and ^13^C NMR spectra of **8**, signals of an isovaleroyl group were identified (*δ*_H_ 2.17 br s, 2.16 br s, 2.06 m, 0.93 d and 0.92 d; *δ*_C_ 172.3, 43.8, 25.6 and 2 × 22.6). In addition, the ^13^C and JMOD spectra suggested a sesquiterpene core consisting of one methyl, five methylenes, five methines and four quaternary carbons (Table 3). Two structural elements could be assigned based on the ^1^H–^1^H COSY spectrum: −CHR-CH_2_-CH_2_− (A *δ*_H_ 3.50, 1.81, 1.58, 2.35 and 2.13) (C-1−C-3) and −CHR-CHR-CHR-CHR-CH_2_− (B *δ*_H_ 2.23, 4.50, 2.79, 5.75, 2.31 and 1.56) (C-5−C-9). Structural parts A and B were connected based on the HMBC correlations observed between C-4 and H-3, and H-5; C-10 and H-2a, H-5, H-9b and H-14; C-12 and H-13a, and H-13b). The location of the ester group was also determined via ^3^*J*_C,H_ coupling between the isovaleroyl CO (*δ*_C_ 172.3) and skeletal proton H-8 (*δ*_H_ 5.75). Comparison of **8** with sesquiterpenes previously isolated from the plant, good agreement was observed with 3*β*-acetoxy-8*β*-isovaleroyloxyreynosin with the exception of the missing acetyl substitution at C-3.

The relative configuration of compound **8** corresponded well with the data reported in the literature. The NOE correlations of H-1/H-5, H-2a and H-9b, H-5/H-7, H-7/H-8 and H-9b clearly indicated the α-orientation of H-1, H-5, H-7, H-8 and H-9b and the *β*-orientation of isovaleroyl group. The *β*-orientation of H-6 and H_3_-14 corroborated with diagnostic Overhauser-effects of the methyl protons with H-6. The coupling constant pattern of **8** is in good agreement with of that of structurally related compounds [50]. All of the above evidence proved the eudesmanolide structure 8*β*-isovaleroyloxyreynosin for this compound, and named as lobatolide G. ECD calculations were performed for (1*R*,5*S*,6*R*,7*R*,8*R*,10*R*)-**8** similarly to the above compounds, but only partial agreements were found, and the experimental spectrum was weak and noisy even after measuring in various solvents and concentrations/settings. Therefore, the absolute configuration was verified by computing for the closely related derivative **9** (see below).

Besides new compounds, volenol (**9**) and (+)-aphanamol I (**10**) were also isolated from *N. lobata*. Their 1D and 2D NMR data were agreed with those published in the literature. The eudesmane-type volenol (**9**) was previously isolated from the aerial parts of *Artemisia rubripes* [51], while the isodaucane-type sesquiterpenoid (+)-aphanamol I (**10**) was first identified from the fruits of *Aphanamixis grandifolia* [52,53].

**Figure 13:**
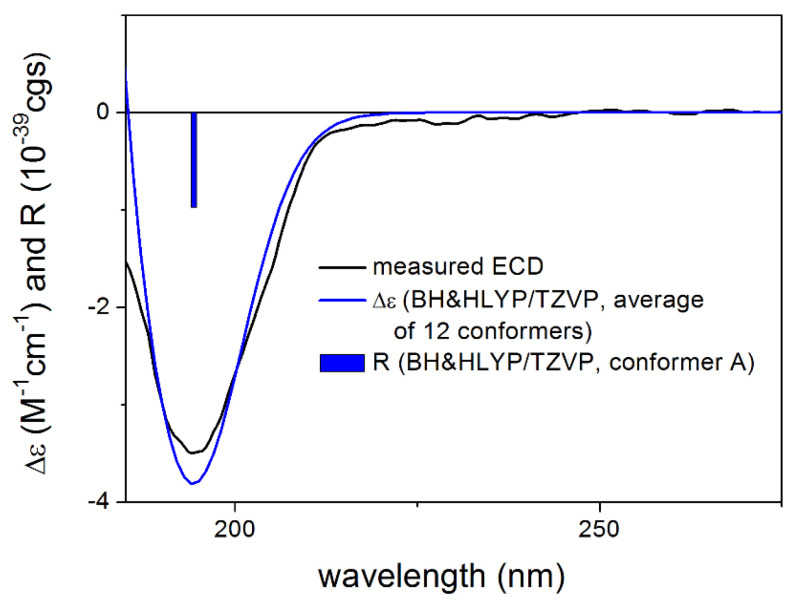
Comparison of the experimental ECD spectrum of **9** measured in MeCN with the BH&HLYP/TZVP PCM/MeCN spectrum of (1*R*,5*S*,6*S*,7*S*,10*R*)-**9** (level of optimization: CAM-B3LYP/TZVP PCM/MeCN). The bars represent the rotational strength values of the lowest energy conformer.

**Figure 14:**
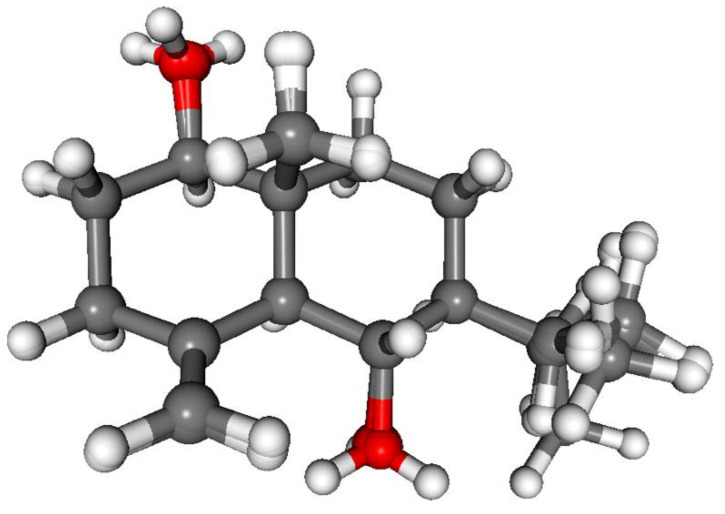
Overlapped geometries of the twelve low-energy CAM-B3LYP/TZVP PCM/MeCN conformers of (1*R*,5*S*,6*S*,7*S*,10*R*)-**9**.

Since the absolute configuration of volenol (**9**) was not described in the literature, the above TDDFT-ECD protocol was applied to (1*R*,5*S*,6*S*,7*S*,10*R*)-**9** (Figure 13). CAM-B3LYP re-optimization of the initial 24 MMFF conformers resulted in 12 low-energy conformers, the ECD spectra of which resembled well the experimental ECD spectrum (Figure 14). Therefore, the absolute configuration of **9** was verified in accordance with the biosynthetic considerations as (1*R*,5*S*,6*S*,7*S*,10*R*).

Naturally occurring germacranolides arise from all-*trans* farnesyl pyrophosphate by a series of biochemical transformations. Compounds belonging to this group (e.g., costunolide) primarily contain two endocyclic double bonds with all-*trans* configuration [e.g., costunolide derivatives]. However, in many cases *cis* configuration also occurs, e.g., in lobatolide C (**3**). The stereochemical skeletal types have distinct conformations, even allowing a certain amount of conformational flexibility [54]. In many cases, one of the endocyclic double bonds has undergone epoxidation as can be found in lobatolide E (**6**) or give a furan-type germacranolide occur in calyculatolide derivatives, like compound **7** (unit B), and **1**–**4**.

### 3.2. Antiproliferative Activity of Isolated Compounds In Vitro

The antiproliferative activities of the isolated compounds **1**–**7**, **9**, **10** and neurolenin B (unit A of compound **7**) were tested on three cervical cancer cell lines of different human papilloma virus (HPV) of different status (SiHa, HeLa, and C33A). Many of the compounds were additionally tested on non-cancerous cell lines NIH/3T3 (mouse embryonic fibroblast) and MRC-5 (human fibroblast) to obtain information concerning the cancer selectivity of the isolated natural products (Table 4). Compound **4** and neurolenin B exhibited higher potency than clinically utilized reference agent cisplatin while compounds **1**, **3**, **6** and **7** exerted similar antiproliferative properties to those of cisplatin. None of the presented natural products displayed substantial cancer selectivity exception for **6** which resulted in IC_50_ values in the range of 3.2–3.6 μM on C33A and SiHa cells but above 11 μM on the two fibroblast cell lines. Neurolenin B was proved to be the most active molecule with IC_50_ values against cancer cells lower than 1.4 μM on all cancer cell lines and NIH/3T3 while its calculated IC_50_ was slightly higher against human fibroblasts, indicating that the cancer selectivity is limited. Based on these antiproliferative activities **6** was selected for additional experiments. HeLa was shown to be the least sensitive cell line from the three cervical cancers. Since HeLa and SiHa cells result from HPV-mediated transformation, HPV-positivity does not explain the different responses of the two cell lines. The most invasive SiHa cell line was chosen further to investigate the possible mechanisms behind the antiproliferative effects. SiHa cells carry HPV-16 genes that contribute to the formation of metastases of this cancer type. HPV-16 oncoproteins promote cervical cancer invasiveness by upregulating specific matrix metalloproteinases (MMPs). HPV-positive cell lines express higher levels of MMP-2, membrane type 1-MMP and tissue inhibitor of metalloproteinase 2 [55].

**Figure 15:**
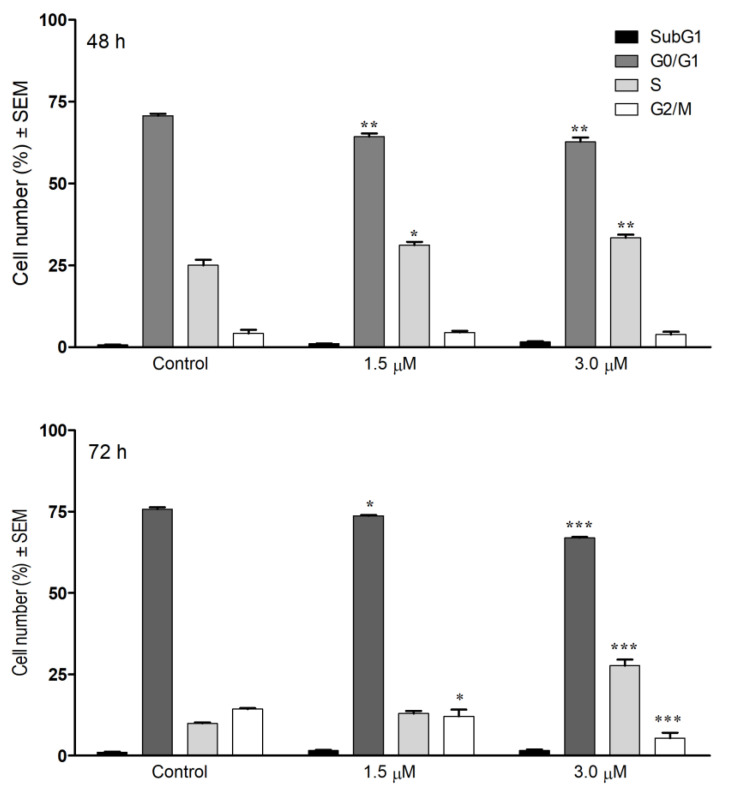
Cell cycle distributions of SiHa cells after treatment with compound **6** for 48 (upper pane) or 72 h (lower panel). Distribution of cell populations in different cell cycle phases. *, ** and *** indicate *p* < 0.05, *p* < 0.01 and *p* < 0.001, respectively, by means of one-way ANOVA followed by Dunnett’s post-hoc test.

Based on the antiproliferative assays, cell cycle analysis was performed on SiHa cells. Compound **6** caused a significant concentration-dependent accumulation of cells in the S phase to the detriment of G0/G1 phase after 48 or 72 h treatment (Figure 15). Cell cycle arrest in the S phase might refer to the cells having a higher tendency to enter S phase due to the dysfunction of cyclin dependent kinases, or it can be a consequence of inhibited DNA synthesis. The molecular mechanism of this cell cycle blockade requires further elucidation. *Iso-seco-*tanapartholide 3-*O*-methyl ether elicited similar S phase accumulation in HL-60 leukemia cells after 24 h of incubation at low concentration (5 μM). This phenomenon was accompanied by G2/M cell cycle phase arrest and subG1 accumulation, which could not be observed in the cell cycle profile of compound **6** [56]. G2/M phase accumulation appears to be a more characteristic effect in cervical cell lines treated by SLs. ROS generation, caspase-3 activation, inhibition of Bcl-2, and enhancement of Bax protein transcription seems a common background of in vitro anticancer activities [57,58]. A sesquiterpene lactone component of *Elephantopus mollis* (EM23) caused cell accumulation in the S phase parallel to the G2/M growth in K562 and HL-60 leukemia cells. ROS generation seems a grounded explanation for the pro-apoptotic effects since ROS quenching with *N*-acetyl-L-cysteine almost completely reversed these observations [59].

**Figure 16:**
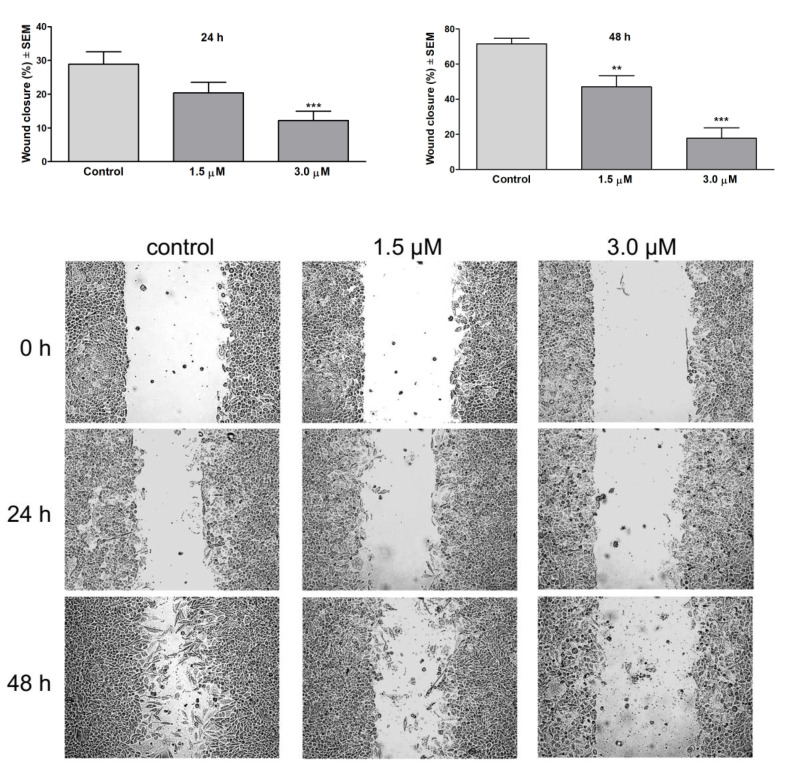
Effect of compound 6 on the migration of SiHa cancer cells after 24 and 48 h of incubation (left and right upper panels, respectively). ** and *** indicate *p* < 0.01 and *p* < 0.001, respectively, by means of one-way ANOVA followed by Dunnett’s post-hoc test. Wound healing assay. Lower panel: representative images of reduced wound healing at 0, 24 and 48 h post-treatment.

Compound **6** with high antiproliferative potency and modest selectivity to cancerous cells were chosen in order to assess its antimetastatic activity. The migration of cancer cells is an important property to form metastases. Therapeutical modalities that target the dissemination of tumor cells from the primary tumor could significantly improve the success of cancer therapy. Wound healing assay represents a simple tool to follow the antimigratory effects of SLs. Wound healing assay was performed on SiHa cell line with compound **6** at 1.5 µM and 3 µM concentrations. The inhibition of wound healing was evaluated by the measurement of the wound surface. 30% wound closure was detected after 24 h in the control wells, while 3 µM treatment with compound **6** resulted in the inhibition of wound healing by causing only 12% closure. After 48 h, the controls showed 70% wound closure; however, the 1.5 and 3 µM treated cells migrated only to 47% and 18% of the wound surface, respectively (Figure 16). These results follow the literature on the effects of sesquiterpenes. Parthenolide and other SLs inhibit cell migration by suppressing the FAK1 signaling pathway in breast cancer cultures [60]. Artesunate, an artemisinin derivative, promotes the downregulation of COX-2 expression associated with lymph node metastasis in cervical cancers. Conversely, COX-2 overexpression and consequent PGE2 formation can be responsible for the enhanced expression of metalloproteinases and vascular endothelial growth factors [61].

## 4. Conclusions

In this study, seven new sesquiterpenoids (**1**–**3** and **5**–**8**), together with three known ones (**4**, **9**, and **10**), were isolated from the dichloromethane phase of methanolic extract of the medicinal plant *N. lobata*. Four of the isolated compounds (**1**–**4**), and unit B of **7** are furanoheliangolide-type compounds, **5**, **6** and unit A of **7** are germacranolides, **8** and **9** are eudesmanes, while **10** is an isodaucane. Compounds **1**, **3**, **4**, and **6**–**8** contain an *α*-methylene-*γ*-lactone ring, while in case of **2** and **5**, the exomethylene unit of the *α*-methylene-*γ*-lactone ring was modified to an oxymethylene part. Compounds are substituted with hydroxy, acetyl and isovaleroyl groups.

In the antiproliferative assay, lobatolides C (**3**), and E (**6**), neurolenin B (unit A of **7**), and lobatolide F (**7**) were proved to be the most active compounds against HeLa, C33A, and SiHa cells. Although, neurolenin B showed the lowest IC_50_ values against cancer cells, it also inhibited normal cells at low concentration. Considering the selectivity of the compounds, lobatolide E (**6**) seems to be the most promising one. Moreover, this compound caused a significant, concentration-dependent accumulation of SiHa cells in the S phase during the cell cycle analysis investigation. The antimetastatic activity of compound **6** was also confirmed by wound healing assay.

## Figures and Tables

**Table 1:** ^1^H NMR data of compounds **1**–**6** in CDCl_3_ at 500 MHz (*δ* in ppm, mult. *J* in Hz).

Position	1	2	3	4	5	6
1						2.93 dd (11.3, 2.3)
2	5.60 s	5.52 s	5.64 s	5.65 s	6.58 d (11.7)	2.35 m, 1.64 m
3					5.97 t (11.7)	5.50 dd (11.4, 5.7)
4	3.01 m	3.01 m			3.09 m	
5	2.59 ddd (14.2, 9.3, 4.8) (α) 2.05 d (14.2) (β)	2.63 ddd (14.2, 9.6, 7.0) 2.03 m	6.00 dd (4.2, 1.8)	5.99 d (3.7, 1.8)	1.79 dt (5.1, 12.3) 1.63 dt (5.1, 12.1)	5.66 d (9.6)
6	4.78 dd (9.3, 4.7)	4.29 dd (9.6, 6.9)	5.71 m	5.24 m	4.56 dd (12.1, 5.3)	5.22 t (9.4)
7	3.30 m	3.16 dd (8.9, 6.9)	3.57 dd (7.6, 4.0)	3.86 dd (4.6, 1.6)	3.04 ddd (10.3, 8.1, 5.3)	3.21 br d (9.4)
8	4.13 t (4.7)	5.07 d (5.4)	4.04 m	5.01 dd (5.2, 1.6)	5.57 d (9.6)	5.74 d (5.7)
9	5.27 d (4.7)	3.94 d (5.4)	5.27 d (3.6)	5.31 d (5.2)	5.57 d (9.6)	2.63 dd (15.2, 5.7) 1.40 dd (15.2, 1.7)
11		2.89 m			2.23 br d (8.1)	
13a	6.30 d (3.0)	3.74 dd (9.4, 4.1)	6.29 d (3.0)	6.35 d (3.0)	3.68 dd (10.2, 5.3)	6.23 d (3.5)
13b	5.35 d (3.0)	3.67 m	5.35 d (3.0)	5.48 d (3.0)	3.34 t (10.2)	5.65 s
14	1.37 s	1.45 s	1.41 s	1.43 s	1.31 s	1.21 s
15	1.40 d (7.0)	1.34 d (7.3)	2.06 s	2.08 s	1.15 d (6.3)	1.87 d (1.3)
OMe		3.40 s			3.39 s	
iVal CO 1′						
2a′	2.38 dd (15.1, 7.2)	2.14 dd (15.3, 7.2)	2.37 dd (15.2, 7.3)	2.15 m	2.00 m	2.27 m
2b′	2.33 dd (15.1, 7.2)	2.10 m	2.31 dd (15.2, 7.2)	2.12 m	2.00 m	2.22 m
3′	2.19 m	2.02 m	2.16 m	2.01 m	2.07 m	2.07 m
4′	1.02 d (6.7)	0.93 d (7.0)	1.01 d (6.6)	0.92 d (7.1)	0.91 d (6.7)	0.95 d (6.4)
5′	1.02 d (6.7)	0.91 d (6.9)	1.01 d (6.6)	0.90 d (7.1)	0.89 d (6.7)	0.94 d (6.4)
8-OH	2.82 d (4.5)		4.05 s			
3-OAc						2.10 s
9-OAc				2.23 s	2.11 s	

**Table 2:** ^13^C NMR data of compounds **1**–**6** in CDCl_3_ at 125 MHz (*δ*_C_ in ppm).

Position	1	2	3	4	5	6
1	203.9	204.2	203.9	202.3	204.7	65.6
2	104.0	103.8	104.6	104.3	125.6	31.4
3	192.8	192.4	185.8	185.4	147.6	77.1
4	31.5	31.4	130.9	134.6	28.7	145.3
5	40.7	41.7	134.3	134.4	38.1	123.9
6	75.3	75.4	74.9	75.5	77.4	75.4
7	47.9	46.1	45.2	44.9	37.5	53.3
8	73.8	75.1	75.9	75.7	68.9	68.5
9	77.1	74.3	77.5	73.6	74.8	43.3
10	88.9	90.7	88.3	88.5	79.3	61.4
11	141.3	49.3	141.2	139.0	40.2	138.2
12	169.2	174.2	170.0	168.7	174.2	171.2
13	122.6	72.1	123.2	123.8	66.3	121.7
14	18.9	18.4	18.4	18.6	23.6	20.2
15	16.1	16.0	19.6	21.2	19.8	13.0
OMe		59.6			59.2	
iVal CO 1′	171.5	171.7	171.7	169.3	171.2	173.2
2′	43.1	43.0	43.1	42.6	42.9	44.1
3′	25.7	25.6	25.6	25.3	24.7	26.7
4′	22.4	22.5	22.4	22.4	22.4	22.7
5′	22.4	22.6	22.4	22.4	22.5	22.7
Ac CO				168.8	170.4	171.8
Ac Me				21.3	20.6	20.8

**Table 3:** ^1^H (500 MHz) and ^13^C (125 MHz) NMR data of compounds **7** and **8** in CDCl_3_ (δ in ppm).

Position	7 (Unit A)		7 (Unit B)		8	
	δ_H_, Mult. (*J* in Hz)	δ_C_	δ_H_, Mult. (*J* in Hz)	δ_C_	δ_H_, Mult. (*J* in Hz)	δ_C_
1	-	205.0	-	203.8	3.50 dd (11.4, 4.7)	78.8
2	6.22 d (11.6)	123.8	5.64 s	105.4	1.81 m, 1.58 m	31.1
3	6.04 t (11.6)	147.3	-	193.0	2.35 m, 2.13 m	33.5
4	2.14 m	25.0	2.81 m	36.8	-	142.0
5	2.13 m, 1.96 m	38.9	3.54 m	44.8	2.23 d (11.0)	53.7
6	5.07 d (5.0)	79.6	4.44 t (4.9)	75.0	4.50 t (11.1)	75.2
7	-	154.2	3.68 m	45.6	2.79 dd (11.1, 2.7)	52.2
8	6.38 d (8.9)	66.9	5.20 br d (5.1)	76.1	5.75 d (2.7)	65.9
9	5.69 d (8.9)	73.1	4.05 br s	74.11	2.31 dd (15.2, 2.2), 1.56 m	40.6
10	-	80.7	-	90.7	-	42.8
11	-	135.7	-	138.0	-	134.8
12	-	172.2	-	168.9	-	170.0
13a	3.05 dd (14.5, 8.8)	23.0	6.29 d (3.0),	124.1	6.15 d (3.2)	119.6
13b	2.48 dd (14.5, 6.8)		5.74 d (3.0)		5.44 d (3.0)	
14	1.32 s	23.9	1.48 s	19.2	5.01 s, 4.94 s	111.1
15	1.14 d (6.4)	22.5	1.40 d (6.8)	10.0	0.96 s	13.8
iVal-CO 1′	s	172.0	-	171.5	-	172.3
2′	2.27 dd (15.8, 6.8), 2.15 m (2H)	43.1	2.08 m (2H)	42.8	2.17 br s, 2.16 br s	43.8
3′	2.02 m	25.5	1.96 m	25.3	2.06 m	25.6
4′	0.94 d (6.7)	22.5	0.90 d (6.7)	22.4	0.93 d (4.2)	22.6
5′	0.93 d (6.7)	22.5	0.87 d (6.7)	22.4	0.92 d (4.2)	22.6
9-OAc		170.3				
	2.15 s	20.7				

**Table 4:** Antiproliferative activity of the isolated compounds determined by MTT assay. Growth inhibition and calculated IC_50_ values of the tested compounds and their 95% confidence intervals (CI).

Comp.	Conc. (μM)	Growth Inhibition Values (%) ± SEM at 10 and 30 μM Calculated IC_50_ Values (μM) [95% CI]				
		HeLa	C33A	SiHa	NIH/3T3	MRC-5
**1**	10	20.39 ± 0.62	90.00 ± 2.54	77.93 ± 0.56	n.t. ^a^	n.t.
	30	81.78 ± 1.70	91.79 ± 0.22	92.10 ± 0.73		
	IC50	16.78 [15.57–18.09]	5.89 [2.32–5.52]	4.86 [4.45–5.31]		
**2**	10	<20 ^b^	<20	<20	n.t.	n.t.
	30	21.55 ± 2.42	36.60 ± 2.95	<20		
**3**	10	91.42 ± 0.34	79.15 ± 3.33	60.65 ± 1.58	92.96 ± 0.56	88.13 ± 1.22
	30	96.58 ± 1.17	94.46 ± 0.21	78.77 ± 1.23	98.15 ± 0.13	97.58 ± 0.10
	IC50	4.76 [4.27–5.32]	6.82 [5.97–7.79]	7.81 [6.81–8.96]	4.12 [3.68–4.61]	6.67 [5.35–8.30]
**4**	10	83.94 ± 1.80	97.27 ± 0.18	79.77 ± 1.47	n.t.	n.t.
	30	90.10 ± 1.03	97.19 ± 0.41	94.62 ± 0.27		
	IC50	5.10 [5.10–6.51]	2.05 [2.91–2.18]	2.22 [1.92–2.56]		
**5**	10	<20	50.50 ± 3.49	<20	n.t.	n.t.
	30	63.86 ± 3.23	89.95 ± 1.4	65.52 ± 2.52		
**6**	10	<20	96.55 ± 0.22	84.19 ± 2.32	40.67 ± 2.92	<20
	30	91.88 ± 1.23	96.72 ± 0.33	84.76 ± 1.89	96.80 ± 0.25	73.05 ± 1.64
	IC50	15.69 [12.51–19.69]	3.53 [2.83–4.39]	3.24 [2.95–3.55]	11.17 [10.64–11.74]	11.94 [10.62–13.43]
**7**	10	91.87 ± 0.72	94.66 ± 0.19	77.17 ± 1.04	90.65 ± 1.20	<20
	30	96.44 ± 0.10	95.18 ± 0.31	93.50 ± 0.42	98.06 ± 0.19	97.63 ± 0.27
	IC50	6.00 [5.44–6.62]	6.62 [4.38–10.00]	6.02 [5.53–6.54]	5.68 [5.36–6.02]	15.42 [12.71–18.71]
**9**	10	<20	<20	<20	n.t.	n.t.
	30	37.27 ± 2.65	26.69 ± 2.73	35.69 ± 1.29		
**10**	10	<20	<20	<20	n.t.	n.t.
	30	36.49 ± 1.55	<20	<20		
Neu B	10	94.00 ± 0.59	98.29 ± 0.03	88.73 ± 0.36	98.11 ± 0.09	94.26 ± 0.23
	30	94.81 ± 0.37	97.91 ± 0.15	93.42 ± 0.33	98.02 ± 0.05	97.46 ± 0.21
	IC50	1.24 [1.07–1.45]	0.47 [4.59–4.93]	1.33 [1.14–1.54]	1.31 [1.22–1.41]	5.18 [4.69–5.73]
Cispl.	10	32.23 ± 1.16	77.17 ± 1.21	60.98 ± 0.92	73.88 ± 0.46	71.24.± 2.89
	30	93.70 ± 0.82	97.50 ± 0.13	88.95 ± 0.53	97.10 ± 0.15	70.65 ± 1.34
	IC50	12.14 [10.18–14.46]	5.85 [5.37–6.38]	4.29 [3.72–4.95]	5.50 [4.46–6.35]	5.77 [4.30–7.74]

^a^ Not tested. ^b^ Cancer cell growth inhibition values less than 20% were considered negligible and are not given numerically.

## Data Availability

Data sharing not applicable.

## Supplementary Materials

The following are available online at https://www.mdpi.com/article/10.3390/pharmaceutics13122088/s1, Figures S1–S55: 1D and 2D NMR, MS and ECD spectra of compounds 1–9. Tables S1–S3, S5, S6 and S8: Comparison of the experimental 13C NMR data. Table S4: Boltzmann populations and optical rotations of the low-energy conformers of (4R,6R,7S,8S,9R,10R,11R)-2 computed at various levels for the CAM-B3LYP/TZVP PCM/CHCl3 re-optimized MMFF conformers. Table S7: Comparison of the experimental 1H NMR data.
