# Supplementary material for: Isolation, Structure Determination of Sesquiterpenes from Neurolaena lobata and Their Antiproliferative, Cell Cycle Arrest-Inducing and Anti-Invasive Properties against Human Cervical Tumor Cells
Source: Pharmaceutics. 2021 Dec 5;13(12):2088. doi: 10.3390/pharmaceutics13122088 (PMC8704432; doi:10.3390/pharmaceutics13122088)
Supplement: Supplementary file 1 [file pharmaceutics-13-02088-s001.zip › pharmaceutics-1443748-supplementary.pdf]

pharmaceutics

MDPI

Supplementary Materials: Isolation, Structure Determination of Sesquiterpenes from *Neurolaena lobata* and Their Antiproliferative, Cell Cycle Arrest-Inducing and Anti-Invasive properties Against Human Cervical Tumor Cells

Andrea Vasas, Ildikó Lajter, Norbert Kúsz, Sándor B. Király, Tibor Kovács, Tibor Kurtán, Noémi Bózsity, Nikolett Nagy, Zsuzsanna Schelz, István Zupkó, Georg Krupitza, Richard Frisch, Attila Mándi, and Judit Hohmann

TABLE OF CONTENTS

Figure S1. ^{1}H NMR spectrum of 1 4

Figure S2. ^{1}H-^{1}H COSY spectrum of 1 4

Figure S3. NOESY spectrum of 1 5

Figure S4. HRMS spectrum of 1 5

Table S1. Comparison of the experimental ^{13}C NMR data of (4*R*,6*R*,7*R*,8*S*,9*R*,10*R*)-1 with the computed values obtained at the 1: mPW1PW91/6-311+G(2d,p) // B3LYP/6-31+G(d,p) and the 2: mPW1PW91/6-311+G(2d,p) SMD/CDCl3 // B3LYP/6-31+G(d,p) levels of theory.................... 6

Figure S5. ^{1}H NMR spectrum of 2.................... 7

Figure S6. ^{1}H-^{1}H COSY spectrum of 2 8

Figure S7. HSQC spectrum of 2.................... 8

Figure S8. HMBC spectrum of 2.................... 9

Figure S9. HRMS spectrum of 2 9

Table S2. Comparison of the experimental ^{13}C NMR data of all carbons of 2 with the mPW1PW91/6-311+G(2d,p) // B3LYP/6-31+G(d,p) ones of the 1: (4*R*,6*R*,7*S*,8*S*,9*R*,10*R*,11*S*)-2, 2: (4*R*,6*R*,7*S*,8*S*,9*R*,10*R*,11*R*)-3, 3: (4*R*,6*R*,7*S*,8*R*,9*R*,10*R*,11*S*)-2, 4: (4*R*,6*R*,7*S*,8*R*,9*R*,10*R*,11*R*)-2, 5: (4*R*,6*R*,7*S*,8*R*,9*S*,10*R*,11*S*)-2, 6: (4*R*,6*R*,7*S*,8*R*,9*S*,10*R*,11*R*)-2, 7: (4*R*,6*R*,7*S*,8*S*,9*S*,10*R*,11*S*)-2 and the 8: (4*R*,6*R*,7*S*,8*S*,9*S*,10*R*,11*R*)-2 stereoisomers. For better comparison, Δδ values over 2.5 were highlighted with yellow and those over 5.0 with red.................... 10

Table S3. Comparison of the experimental ^{13}C NMR data of all carbons of 2 with the mPW1PW91/6-311+G(2d,p) SMD/$CDCl_3$ // B3LYP/6-31+G(d,p) ones of the 1: (4*R*,6*R*,7*S*,8*S*,9*R*,10*R*,11*S*)-2, 2: (4*R*,6*R*,7*S*,8*S*,9*R*,10*R*,11*R*)-2, 3: (4*R*,6*R*,7*S*,8*R*,9*R*,10*R*,11*S*)-2, 4: (4*R*,6*R*,7*S*,8*R*,9*R*,10*R*,11*R*)-2, 5: (4*R*,6*R*,7*S*,8*R*,9*S*,10*R*,11*S*)-2, 6: (4*R*,6*R*,7*S*,8*R*,9*S*,10*R*,11*R*)-2, 7: (4*R*,6*R*,7*S*,8*S*,9*S*,10*R*,11*S*)-2 and the 8: (4*R*,6*R*,7*S*,8*S*,9*S*,10*R*,11*R*)-2 stereoisomers. For better comparison, Δδ values over 2.5 were highlighted with yellow and those over 5.0 with red.................... 11

Table S4. Boltzmann populations and optical rotations of the low-energy conformers of (4*R*,6*R*,7*S*,8*S*,9*R*,10*R*,11*R*)-2 computed at various levels for the CAM-B3LYP/TZVP PCM/$CHCl_3$ re-optimized MMFF conformers.................... 12

Figure S10. ^{1}H NMR spectrum of 3.................... 13

Figure S11. ^{13}C NMR spectrum of 3 13

Figure S12. ^{1}H-^{1}H COSY spectrum of 3 14

Figure S13. HSQC spectrum of 3........ 14

Figure S14. HMBC spectrum of 3........ 15

Figure S15. NOESY spectrum of 3 15

Figure S16. HRMS spectrum of 3 16

Figure S17. ^{1}H NMR spectrum of 4........ 16

Figure S18. ^{1}H-^{1}H COSY spectrum of 4 17

Figure S19. NOESY spectrum of 4 17

Figure S20. HRMS spectrum of 4 18

Figure S21. Overlapped geometries of the conformers of the two conformer groups of (6*R*,7*S*,8*S*,9*R*,10*R*)-4. Group A: confs. A-F, I, J; group B: confs. G, H. Level of optimization: CAM-B3LYP/TZVP PCM/MeCN. 18

Figure S22. Comparison of the experimental ECD spectrum of 4 measured in MeCN with the CAM-B3LYP/TZVP PCM/MeCN spectra of conformers A and G of (6*R*,7*S*,8*S*,9*R*,10*R*)-4, as the lowest-energy representatives of groups A and B. Level of optimization: CAM-B3LYP/TZVP PCM/MeCN. 19

Figure S23. ^{1}H NMR spectrum of 5........ 19

Figure S24. JMOD spectrum of 5........ 20

Figure S25. ^{1}H-^{1}H COSY spectrum of 5 20

Figure S26. HSQC spectrum of 5........ 21

Figure S27. HMBC spectrum of 5........ 22

Figure S28. NOESY spectrum of 5 22

Figure S29. HRMS spectrum of 5 22

Table S5. Comparison of the experimental ^{13}C NMR data of all carbons of 5 with the mPW1PW91/6-311+G(2d,p) // B3LYP/6-31+G(d,p) ones of the 1: (4*R*,6*R*,7*S*,8*S*,9*R*,10*R*,11*S*)-5, 2: (4*R*,6*R*,7*S*,8*S*,9*R*,10*R*,11*R*)-5, 3: (4*R*,6*R*,7*S*,8*R*,9*R*,10*R*,11*S*)-5, 4: (4*R*,6*R*,7*S*,8*R*,9*R*,10*R*,11*R*)-5, 5: (4*R*,6*R*,7*S*,8*R*,9*S*,10*R*,11*S*)-5, 6: (4*R*,6*R*,7*S*,8*R*,9*S*,10*R*,11*R*)-5, 7: (4*R*,6*R*,7*S*,8*S*,9*S*,10*R*,11*S*)-5 and the 8: (4*R*,6*R*,7*S*,8*S*,9*S*,10*R*,11*R*)-5 stereoisomers. For better comparison, Δδ values over 2.5 were highlighted with yellow and those over 5.0 with red........ 24

Table S6. Comparison of the experimental ^{13}C NMR data of all carbons of 5 with the mPW1PW91/6-311+G(2d,p) SMD/$CDCl_3$ // B3LYP/6-31+G(d,p) ones of the 1: (4*R*,6*R*,7*S*,8*S*,9*R*,10*R*,11*S*)-5, 2: (4*R*,6*R*,7*S*,8*S*,9*R*,10*R*,11*R*)-5, 3: (4*R*,6*R*,7*S*,8*R*,9*R*,10*R*,11*S*)-5, 4: (4*R*,6*R*,7*S*,8*R*,9*R*,10*R*,11*R*)-5, 5: (4*R*,6*R*,7*S*,8*R*,9*S*,10*R*,11*S*)-5, 6: (4*R*,6*R*,7*S*,8*R*,9*S*,10*R*,11*R*)-5, 7: (4*R*,6*R*,7*S*,8*S*,9*S*,10*R*,11*S*)-5 and the 8: (4*R*,6*R*,7*S*,8*S*,9*S*,10*R*,11*R*)-5 stereoisomers. For better comparison, Δδ values over 2.5 were highlighted with yellow and those over 5.0 with red........ 25

Table S7. Comparison of the experimental ^{1}H NMR data of all hydrogens except for the OH hydrogen of 5 with the mPW1PW91/6-311+G(2d,p) // B3LYP/6-31+G(d,p) ones of the 1: (4*R*,6*R*,7*S*,8*S*,9*R*,10*R*,11*S*)-5, 2: (4*R*,6*R*,7*S*,8*S*,9*R*,10*R*,11*R*)-5, 3: (4*R*,6*R*,7*S*,8*R*,9*R*,10*R*,11*S*)-5, 4: (4*R*,6*R*,7*S*,8*R*,9*R*,10*R*,11*R*)-5, 5: (4*R*,6*R*,7*S*,8*R*,9*S*,10*R*,11*S*)-5, 6: (4*R*,6*R*,7*S*,8*R*,9*S*,10*R*,11*R*)-5, 7: (4*R*,6*R*,7*S*,8*S*,9*S*,10*R*,11*S*)-5 and the 8: (4*R*,6*R*,7*S*,8*S*,9*S*,10*R*,11*R*)-5 stereoisomers. Shielding constants related to the hydrogen atoms in the

methyl groups were averaged. For better comparison, Δδ values over 0.3 were highlighted with yellow and those over 0.6 with red........ 26

Table S8. Comparison of the experimental ^{13}C NMR data of all carbons of 5 with the mPW1PW91/6-311+G(2d,p) SMD/$CDCl_3$ // B3LYP/6-31+G(d,p) ones of the 1: (4*R*,6*R*,7*S*,8*S*,9*R*,10*R*,11*S*)-5, 2: (4*R*,6*R*,7*S*,8*S*,9*R*,10*R*,11*R*)-5, 3: (4*R*,6*R*,7*S*,8*R*,9*R*,10*R*,11*S*)-5, 4: (4*R*,6*R*,7*S*,8*R*,9*R*,10*R*,11*R*)-5, 5: (4*R*,6*R*,7*S*,8*R*,9*S*,10*R*,11*S*)-5, 6: (4*R*,6*R*,7*S*,8*R*,9*S*,10*R*,11*R*)-5, 7: (4*R*,6*R*,7*S*,8*S*,9*S*,10*R*,11*S*)-5 and the 8: (4*R*,6*R*,7*S*,8*S*,9*S*,10*R*,11*R*)-5 stereoisomers. For better comparison, Δδ values over 2.5 were highlighted with yellow and those over 5.0 with red........ 28

Figure S30. ^{1}H NMR spectrum of 6........ 29

Figure S31. ^{13}C NMR spectrum of 6........ 30

Figure S32. ^{1}H-^{1}H COSY spectrum of 6........ 30

Figure S33. HSQC spectrum of 6........ 31

Figure S34. HMBC spectrum of 6........ 31

Figure S35. NOESY spectrum of 6........ 32

Figure S36. HRMS spectrum of 6........ 32

Figure S37. ^{1}H NMR spectrum of 7........ 33

Figure S38. JMOD spectrum of 7........ 33

Figure S39. HSQC spectrum of 7........ 34

Figure S40. HMBC spectrum of 7........ 34

Figure S41. ^{1}H NMR spectrum of 8........ 35

Figure S42. JMOD spectrum of 8........ 36

Figure S43. ^{1}H-^{1}H COSY spectrum of 8........ 36

Figure S44. HSQC spectrum of 8........ 37

Figure S45. HMBC spectrum of 8........ 38

Figure S46. HRMS spectrum of 8........ 38

Figure S47. ^{1}H NMR spectrum of 9........ 38

Figure S48. ^{1}H-^{1}H COSY spectrum of 9........ 39

Figure S49. HSQC spectrum of 9........ 40

Figure S50. HMBC spectrum of 9........ 41

Figure S51. NOESY spectrum of 9........ 41

Figure S52. ^{1}H NMR spectrum of 10........ 42

Figure S53. JMOD spectrum of 10........ 42

Figure S54. HSQC spectrum of 10........ 43

Figure S55. HMBC spectrum of 10........ 43

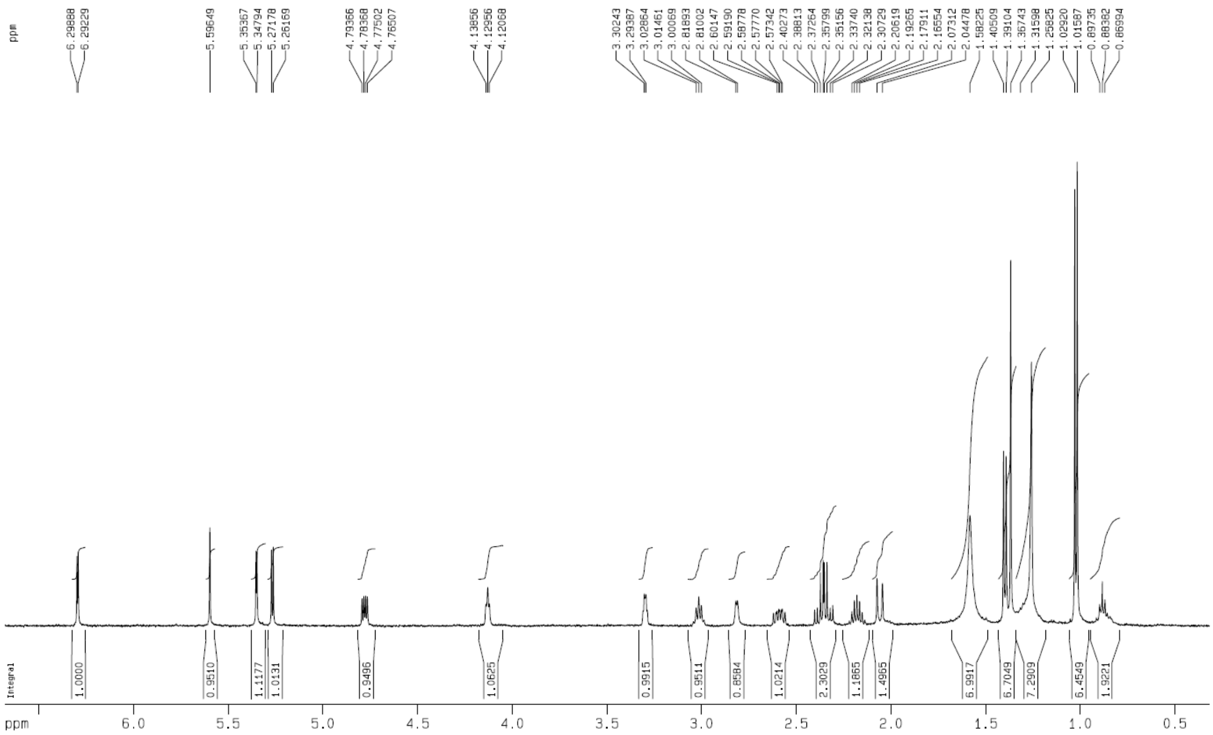

Figure S1. ^{1}H NMR spectrum of **1**

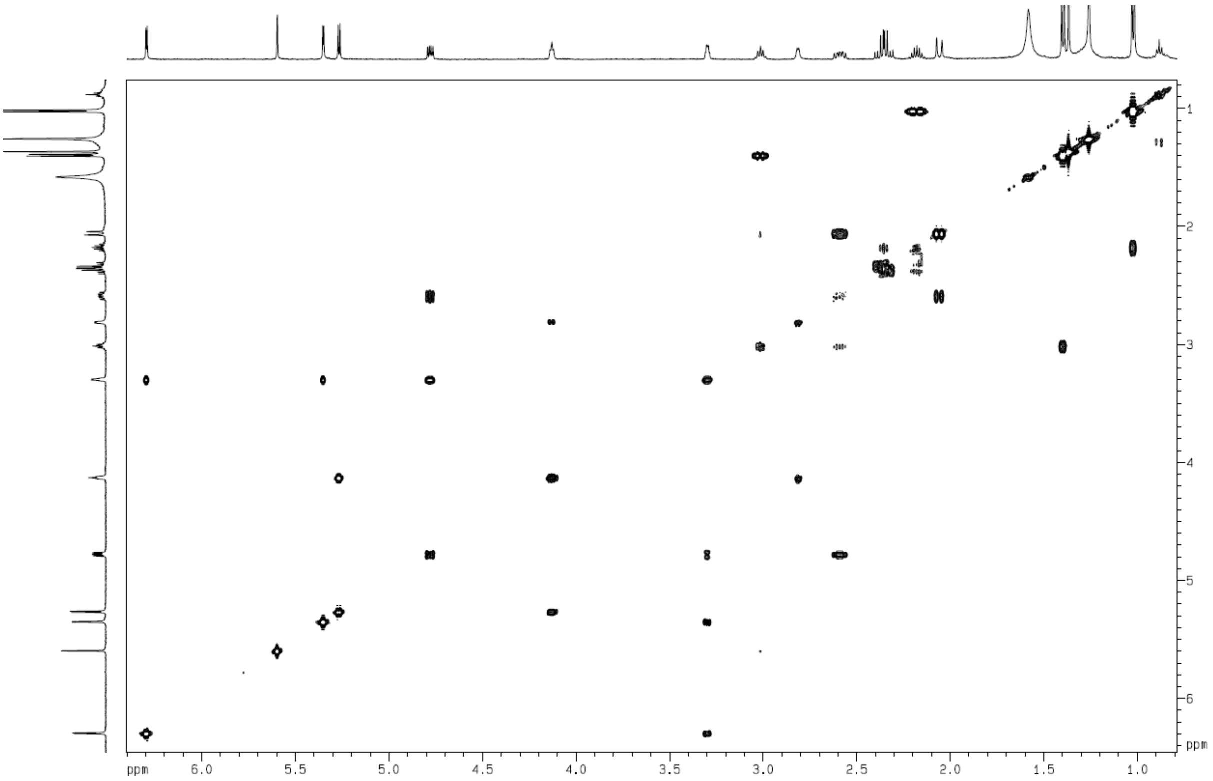

Figure S2. ^{1}H-^{1}H COSY spectrum of **1**

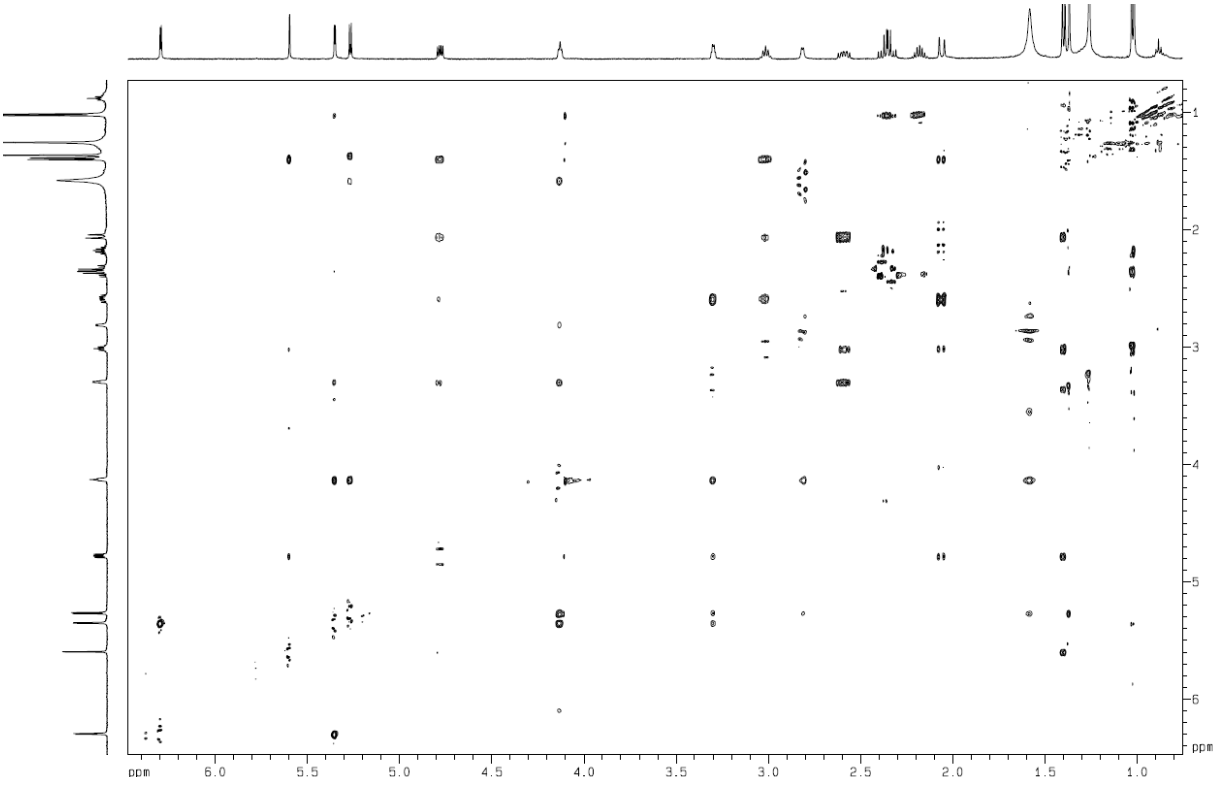

Figure S3. NOESY spectrum of **1**

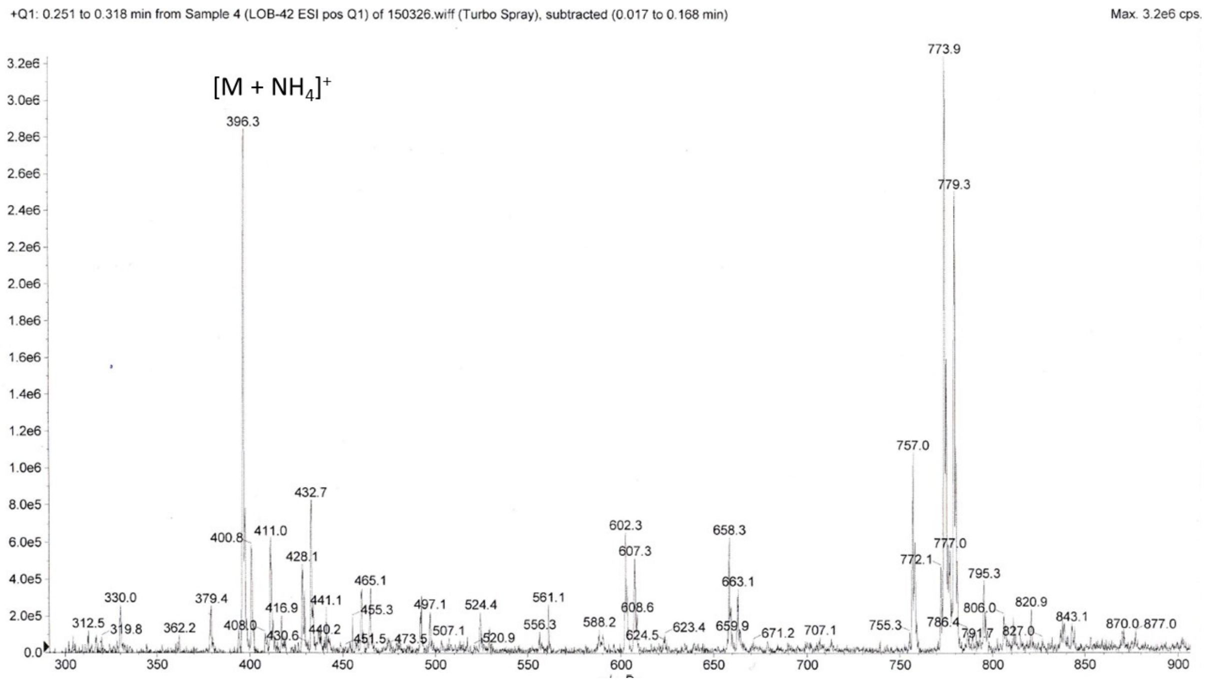

Figure S4. HRMS spectrum of **1**

Table S1. Comparison of the experimental ^{13}C NMR data of (4*R*,6*R*,7*R*,8*S*,9*R*,10*R*)-**1** with the computed values obtained at the 1: mPW1PW91/6-311+G(2d,p) // B3LYP/6-31+G(d,p) and the 2: mPW1PW91/6-311+G(2d,p) SMD/CDCl3 // B3LYP/6-31+G(d,p) levels of theory.

Numbering	Experimental	δ1	δ2	Δδ1	Δδ2
C-1	203.9	200.86	200.33	3.04	3.57
C-2	104.0	104.35	102.83	0.35	1.17
C-3	192.8	194.32	193.79	1.52	0.99
C-4	31.5	34.63	35.02	3.13	3.52
C-5	40.7	41.76	41.46	1.06	0.76
C-6	75.3	71.75	72.50	3.55	2.80
C-7	47.9	49.38	49.30	1.48	1.40
C-8	73.8	77.49	76.62	3.69	2.82
C-9	77.1	78.37	77.64	1.27	0.54
C-10	88.9	89.93	89.91	1.03	1.01
C-11	141.3	146.18	143.88	4.88	2.58
C-12	169.2	167.14	167.51	2.06	1.69
C-13	122.6	123.08	124.21	0.48	1.61
C-14	18.9	17.83	17.97	1.07	0.93
C-15	16.1	14.43	14.54	1.67	1.56
C-1'	171.5	173.21	172.46	1.71	0.96
C-2'	43.1	42.36	42.32	0.74	0.78
C-3'	25.7	27.67	28.39	1.97	2.69
C-4'	22.4	20.55	20.38	1.85	2.02
C-5'	22.4	20.53	20.41	1.87	1.99
CMAE				1.92	1.77

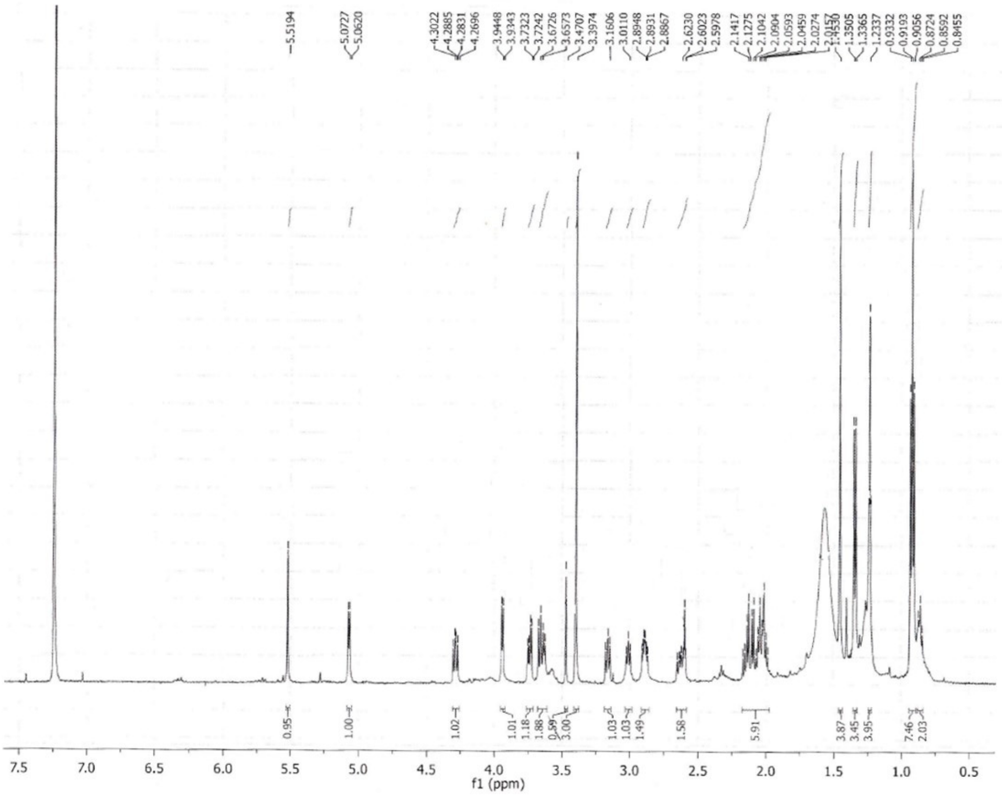

Figure S5. ^{1}H NMR spectrum of **2**

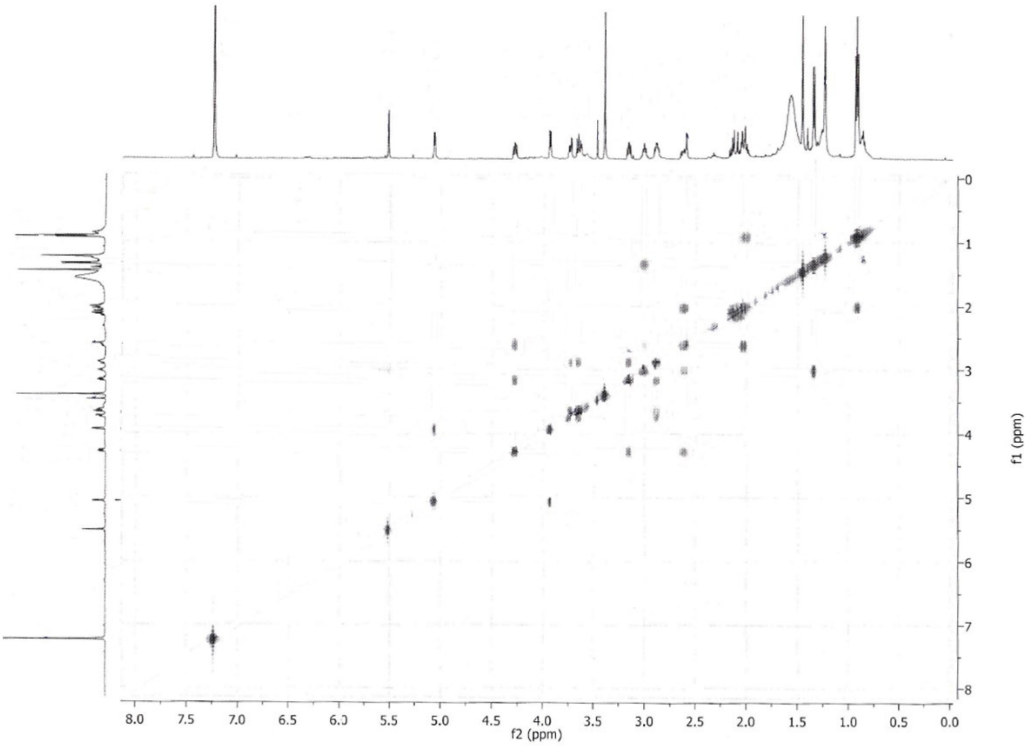

Figure S6. ^{1}H-^{1}H COSY spectrum of **2**

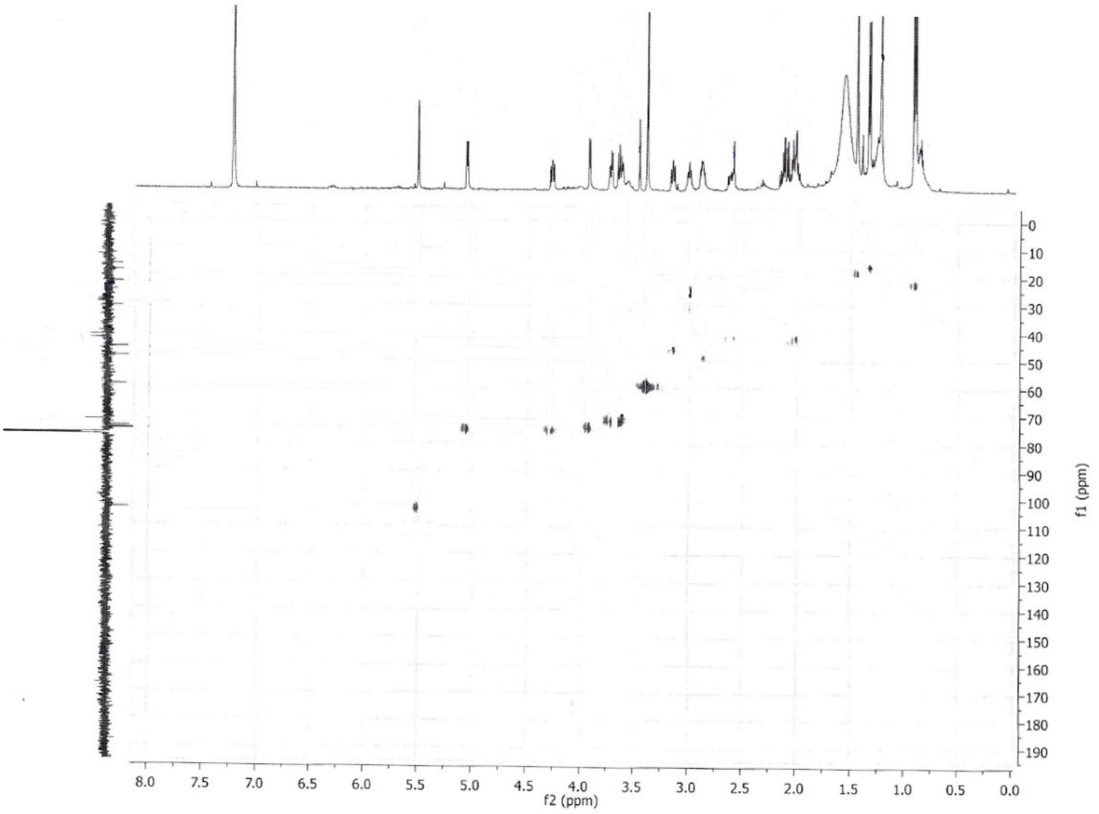

Figure S7. HSQC spectrum of **2**

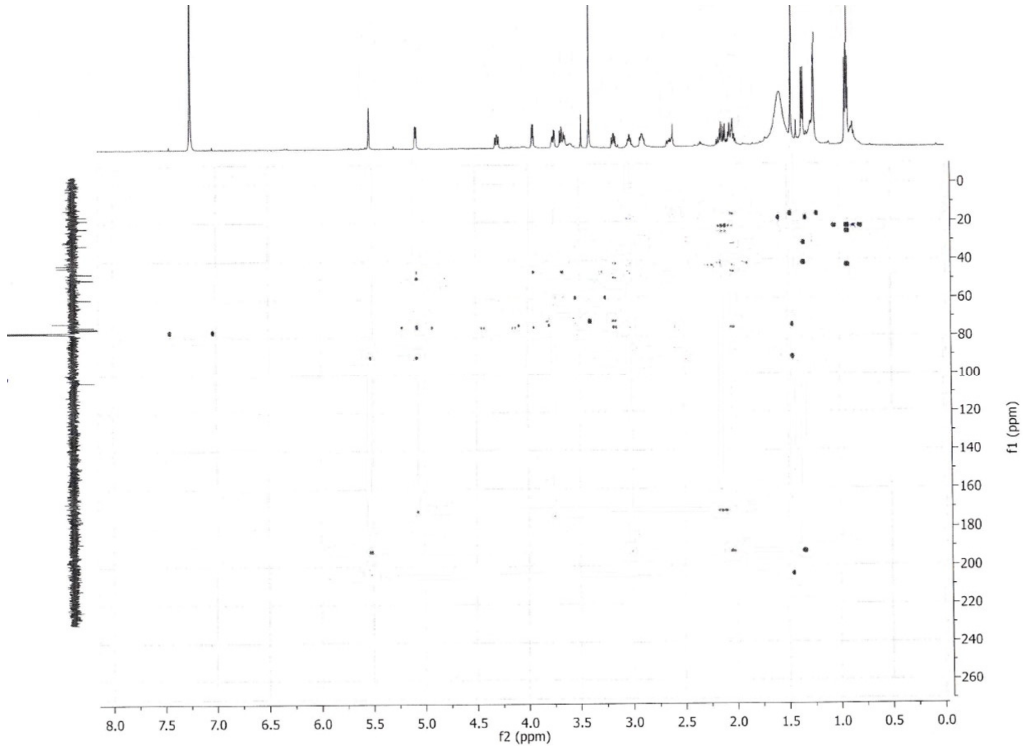

Figure S8. HMBC spectrum of **2**

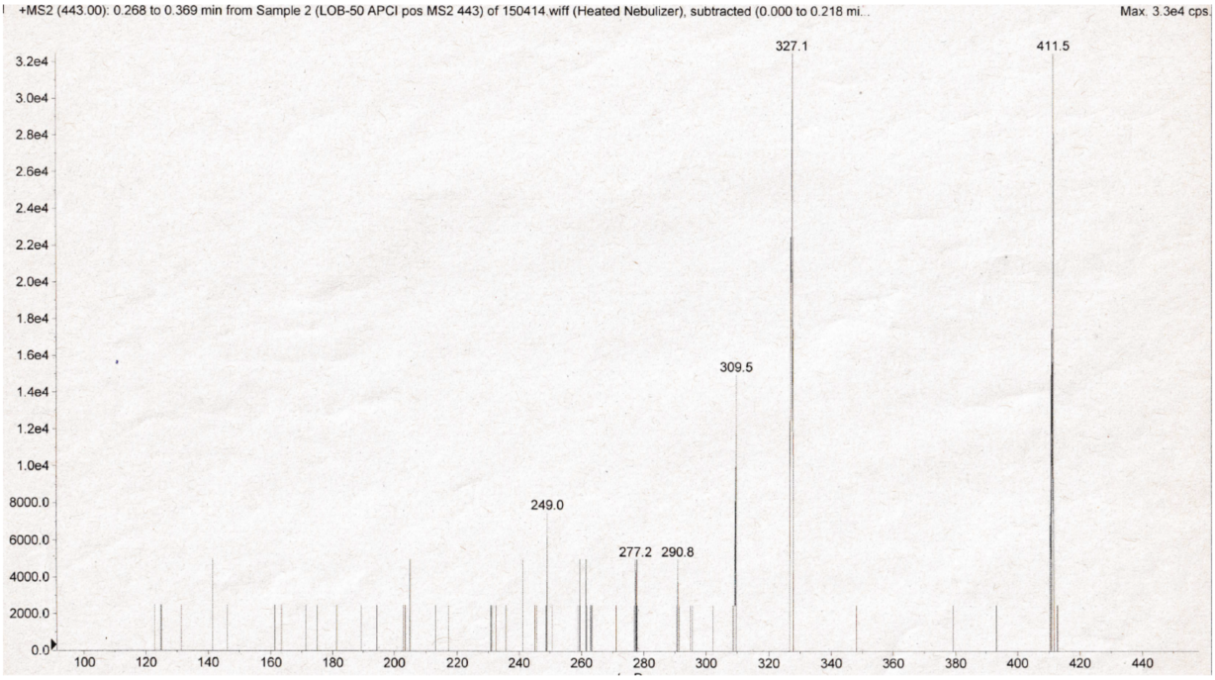

Figure S9. HRMS spectrum of **2**

Table S2. Comparison of the experimental ^{13}C NMR data of all carbons of **2** with the mPW1PW91/6-311+G(2d,p) // B3LYP/6-31+G(d,p) ones of the 1: (4*R*,6*R*,7*S*,8*S*,9*R*,10*R*,11*S*)-**2**, 2: (4*R*,6*R*,7*S*,8*S*,9*R*,10*R*,11*R*)-3, 3: (4*R*,6*R*,7*S*,8*R*,9*R*,10*R*,11*S*)-**2**, 4: (4*R*,6*R*,7*S*,8*R*,9*R*,10*R*,11*R*)-**2**, 5: (4*R*,6*R*,7*S*,8*R*,9*S*,10*R*,11*S*)-**2**, 6: (4*R*,6*R*,7*S*,8*R*,9*S*,10*R*,11*R*)-**2**, 7: (4*R*,6*R*,7*S*,8*S*,9*S*,10*R*,11*S*)-**2** and the 8: (4*R*,6*R*,7*S*,8*S*,9*S*,10*R*,11*R*)-**2** stereoisomers. For better comparison, Δδ values over 2.5 were highlighted with yellow and those over 5.0 with red.

Numbering	Exp	$Calc_1$	$Calc_2$	$Calc_3$	$Calc_4$	$Calc_5$	$Calc_6$	$Calc_7$	$Calc_8$	$\Delta\delta_1$	$\Delta\delta_2$	$\Delta\delta_3$	$\Delta\delta_4$	$\Delta\delta_5$	$\Delta\delta_6$	$\Delta\delta_7$	$\Delta\delta_8$
C-1	204.2	201.54	201.62	201.79	201.35	203.73	204.08	205.94	206.04	2.66	2.58	2.41	2.85	0.47	0.12	1.74	1.84
C-2	103.8	104.99	104.42	105.79	105.12	104.20	105.40	104.28	103.78	1.19	0.62	1.99	1.32	0.40	1.60	0.48	0.02
C-3	192.4	192.77	192.52	195.04	195.47	198.80	197.37	195.15	195.46	0.37	0.12	2.64	3.07	6.40	4.97	2.75	3.06
C-4	31.4	33.75	34.07	34.20	34.48	34.53	34.42	34.66	34.73	2.35	2.67	2.80	3.08	3.13	3.02	3.26	3.33
C-5	41.7	39.70	42.36	41.39	42.10	40.94	41.56	40.92	42.63	2.00	0.66	0.31	0.40	0.76	0.14	0.78	0.93
C-6	75.4	74.19	74.62	80.90	78.97	79.84	78.82	72.99	72.80	1.21	0.78	5.50	3.57	4.44	3.42	2.41	2.60
C-7	46.1	42.87	48.87	42.81	49.80	48.22	47.74	47.58	49.11	3.23	2.77	3.29	3.70	2.12	1.64	1.48	3.01
C-8	75.1	65.93	73.85	78.73	73.81	71.43	70.43	70.73	77.50	9.17	1.25	3.63	1.29	3.67	4.67	4.37	2.40
C-9	74.3	75.33	75.47	77.51	77.62	79.07	79.03	81.64	81.01	1.03	1.17	3.21	3.32	4.77	4.73	7.34	6.71
C-10	90.7	94.21	93.09	95.68	95.31	89.18	89.56	89.18	87.52	3.51	2.39	4.98	4.61	1.52	1.14	1.52	3.18
C-11	49.3	43.31	49.27	43.56	44.27	47.56	45.36	43.85	49.20	5.99	0.03	5.74	5.03	1.74	3.94	5.45	0.10
C-12	174.2	173.62	172.26	173.53	173.03	175.06	174.27	173.78	173.60	0.58	1.94	0.67	1.17	0.86	0.07	0.42	0.60
C-13	72.1	68.45	74.76	68.98	74.76	70.63	67.97	67.58	69.12	3.65	2.66	3.12	2.66	1.47	4.13	4.52	2.98
C-14	18.4	17.24	16.74	15.52	15.25	19.61	19.28	21.22	20.36	1.16	1.66	2.88	3.15	1.21	0.88	2.82	1.96
C-15	16.0	15.65	14.65	15.45	14.83	15.22	14.53	15.28	14.25	0.35	1.35	0.55	1.17	0.78	1.47	0.72	1.75
OMe	59.6	57.07	57.21	56.58	56.88	56.75	56.70	56.77	56.77	2.53	2.39	3.02	2.72	2.85	2.90	2.83	2.83
ival CO 1'	171.7	173.57	173.69	174.08	174.39	173.60	173.12	173.35	174.60	1.87	1.99	2.38	2.69	1.90	1.42	1.65	2.90
C-2'	43.0	41.88	41.94	42.77	42.52	42.35	42.27	42.20	42.00	1.12	1.06	0.23	0.48	0.65	0.73	0.80	1.00
C-3'	25.6	27.05	27.03	27.85	27.94	26.97	27.43	27.58	27.51	1.45	1.43	2.25	2.34	1.37	1.83	1.98	1.91
C-4'	22.5	20.32	20.23	20.33	20.35	20.38	20.36	20.48	20.31	2.18	2.27	2.17	2.15	2.12	2.14	2.02	2.19
C-5'	22.6	20.54	20.63	20.56	20.63	20.87	20.92	20.61	20.54	2.06	1.97	2.04	1.97	1.73	1.68	1.99	2.06
CMAE	N/A	N/A	N/A	N/A	N/A	N/A	N/A	N/A	N/A	2.36	1.61	2.66	2.51	2.11	2.22	2.44	2.25

Table S3. Comparison of the experimental ^{13}C NMR data of all carbons of **2** with the mPW1PW91/6-311+G(2d,p) SMD/$CDCl_3$ // B3LYP/6-31+G(d,p) ones of the 1: (4*R*,6*R*,7*S*,8*S*,9*R*,10*R*,11*S*)-**2**, 2: (4*R*,6*R*,7*S*,8*S*,9*R*,10*R*,11*R*)-**2**, 3: (4*R*,6*R*,7*S*,8*R*,9*R*,10*R*,11*S*)-**2**, 4: (4*R*,6*R*,7*S*,8*R*,9*R*,10*R*,11*R*)-**2**, 5: (4*R*,6*R*,7*S*,8*R*,9*S*,10*R*,11*S*)-**2**, 6: (4*R*,6*R*,7*S*,8*R*,9*S*,10*R*,11*R*)-**2**, 7: (4*R*,6*R*,7*S*,8*S*,9*S*,10*R*,11*S*)-**2** and the 8: (4*R*,6*R*,7*S*,8*S*,9*S*,10*R*,11*R*)-**2** stereoisomers. For better comparison, Δδ values over 2.5 were highlighted with yellow and those over 5.0 with red.

Numbering	Exp	Calc$_1$	Calc$_2$	Calc$_3$	Calc$_4$	Calc$_5$	Calc$_6$	Calc$_7$	Calc$_8$	$\Delta\delta_1$	$\Delta\delta_2$	$\Delta\delta_3$	$\Delta\delta_4$	$\Delta\delta_5$	$\Delta\delta_6$	$\Delta\delta_7$	$\Delta\delta_8$
C-1	204.2	200.35	200.74	201.00	200.59	203.05	203.20	204.69	204.76	3.85	3.46	3.20	3.61	1.15	1.00	0.49	0.56
C-2	103.8	103.37	102.85	104.24	103.66	102.91	103.99	103.02	102.39	0.43	0.95	0.44	0.14	0.89	0.19	0.78	1.41
C-3	192.4	192.36	192.53	195.36	195.14	198.71	197.18	194.70	194.88	0.04	0.13	2.96	2.74	6.31	4.78	2.30	2.48
C-4	31.4	34.38	34.71	34.76	35.08	35.19	35.04	35.26	35.23	2.98	3.31	3.36	3.68	3.79	3.64	3.86	3.83
C-5	41.7	39.41	41.90	40.89	41.74	40.80	41.55	40.71	42.55	2.29	0.20	0.81	0.04	0.90	0.15	0.99	0.85
C-6	75.4	74.69	74.75	81.54	79.22	80.34	79.01	73.65	73.34	0.71	0.65	6.14	3.82	4.94	3.61	1.75	2.06
C-7	46.1	42.84	48.73	42.52	49.47	47.72	47.72	47.22	48.88	3.26	2.63	3.58	3.37	1.62	1.62	1.12	2.78
C-8	75.1	65.59	73.23	78.07	73.70	71.08	70.12	70.63	76.98	9.51	1.87	2.97	1.40	4.02	4.98	4.47	1.88
C-9	74.3	74.75	74.95	77.48	77.32	77.98	77.85	80.54	79.90	0.45	0.65	3.18	3.02	3.68	3.55	6.24	5.60
C-10	90.7	93.89	92.47	94.81	94.26	88.79	89.06	88.53	87.25	3.19	1.77	4.11	3.56	1.91	1.64	2.17	3.45
C-11	49.3	43.62	49.58	43.57	44.90	48.06	45.82	44.09	49.71	5.68	0.28	5.73	4.40	1.24	3.48	5.21	0.41
C-12	174.2	174.40	173.03	174.39	173.82	176.34	175.36	174.64	174.69	0.20	1.17	0.19	0.38	2.14	1.16	0.44	0.49
C-13	72.1	67.58	73.51	67.81	73.57	70.19	67.33	67.09	68.31	4.52	1.41	4.29	1.47	1.91	4.77	5.01	3.79
C-14	18.4	17.27	16.76	15.42	15.08	19.49	19.08	21.02	20.40	1.13	1.64	2.98	3.32	1.09	0.68	2.62	2.00
C-15	16.0	15.71	14.66	15.55	14.90	15.21	14.55	15.36	14.32	0.29	1.34	0.45	1.10	0.79	1.45	0.64	1.68
OMe	59.6	56.78	56.88	56.20	56.53	56.25	56.22	56.39	56.31	2.82	2.72	3.40	3.07	3.35	3.38	3.21	3.29
ival CO 1'	171.7	172.33	172.72	173.17	173.28	172.74	172.53	172.57	173.68	0.63	1.02	1.47	1.58	1.04	0.83	0.87	1.98
C-2'	43.0	42.02	42.08	42.66	42.59	42.39	42.24	42.23	42.04	0.98	0.92	0.34	0.41	0.61	0.76	0.77	0.96
C-3'	25.6	27.54	27.79	28.40	28.72	27.53	27.85	27.98	28.33	1.94	2.19	2.80	3.12	1.93	2.25	2.38	2.73
C-4'	22.50	20.04	19.94	20.13	20.20	20.11	20.10	20.21	20.13	2.46	2.56	2.37	2.30	2.39	2.40	2.29	2.37
C-5'	22.60	20.33	20.34	20.38	20.48	20.63	20.60	20.35	20.38	2.27	2.26	2.22	2.12	1.97	2.00	2.25	2.22
CMAE	N/A	N/A	N/A	N/A	N/A	N/A	N/A	N/A	N/A	2.36	1.58	2.71	2.32	2.27	2.30	2.37	2.23

Table S4. Boltzmann populations and optical rotations of the low-energy conformers of (4*R*,6*R*,7*S*,8*S*,9*R*,10*R*,11*R*)-**2** computed at various levels for the CAM-B3LYP/TZVP PCM/$CHCl_3$ re-optimized MMFF conformers.

Conformer	Boltzmann population	B3LYP/TZVP	BH&HLYP/TZVP	CAM-B3LYP/TZVP	PBE0/TZVP
Conf. A	18.86	66.71	47.37	58.29	66.55
Conf. B	14.96	51.10	39.86	44.73	53.86
Conf. C	12.88	80.64	67.20	73.79	83.32
Conf. D	10.89	89.08	69.33	80.58	89.80
Conf. E	9.60	28.53	22.21	25.67	31.50
Conf. F	7.12	89.35	67.22	80.06	89.12
Conf. G	4.76	74.94	60.92	69.08	76.70
Conf. H	1.75	84.33	66.09	72.93	86.39
Conf. I	1.66	101.92	75.56	88.23	100.99
Conf. J	1.54	112.86	92.27	101.06	114.56
Conf. K	1.24	89.46	67.74	79.36	89.30
Conf. L	1.13	58.77	45.01	50.65	60.59
Conf. M	1.08	69.29	56.44	62.80	71.66
Conf. N	1.08	70.05	56.54	62.34	73.22
Conf. O	1.07	50.92	44.93	49.75	53.26
Conf. P	1.00	126.30	99.34	112.50	126.35
Conf. Q	0.94	30.83	18.37	27.57	30.56
Average	N/A	69.09	53.48	61.75	70.52

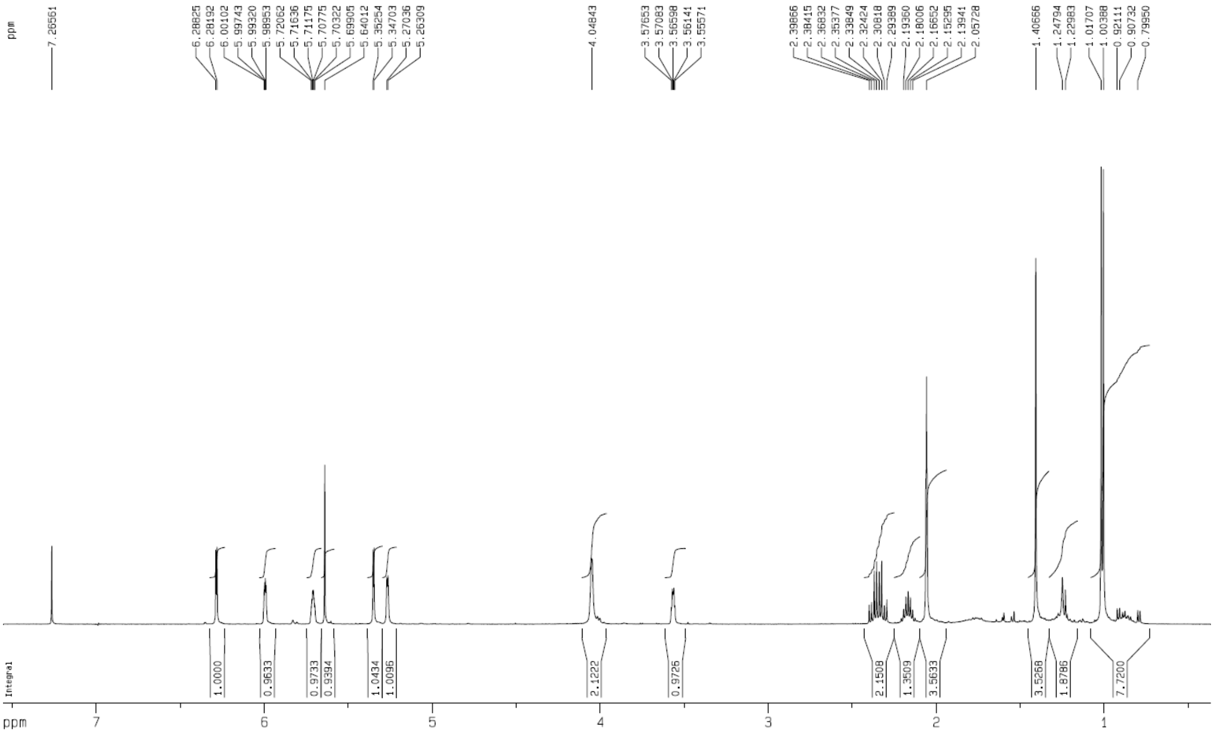

Figure S10. ^{1}H NMR spectrum of **3**

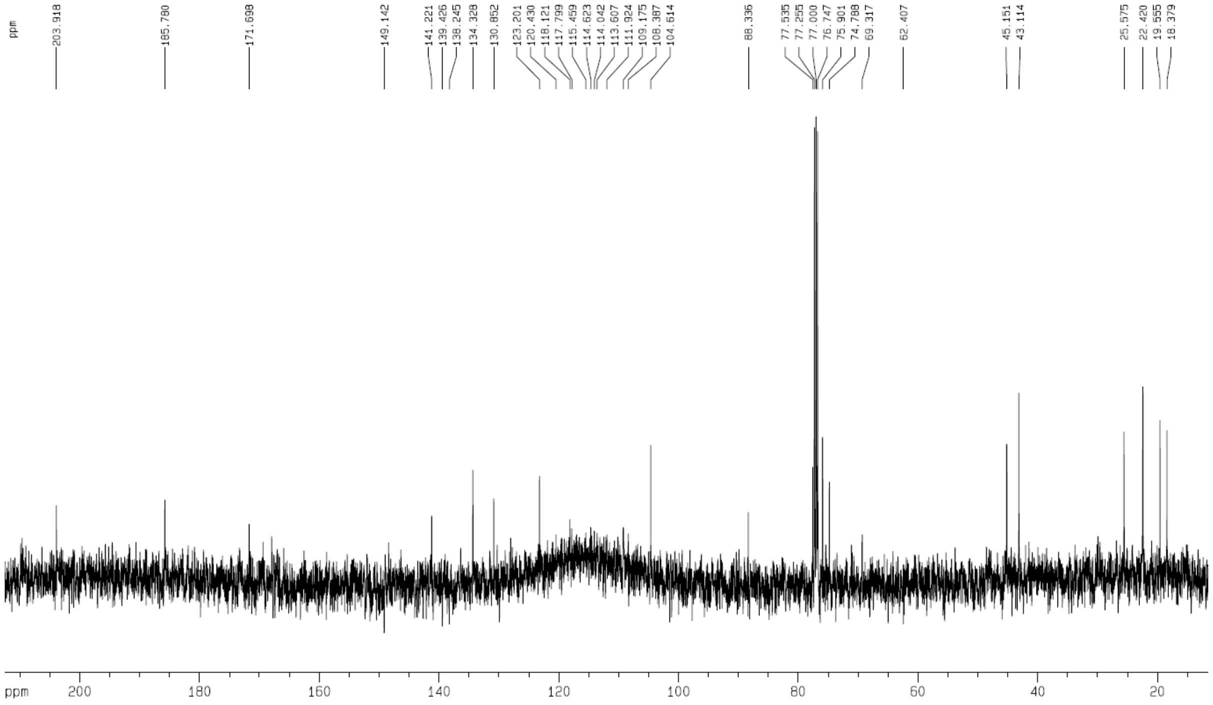

Figure S11. ^{13}C NMR spectrum of **3**

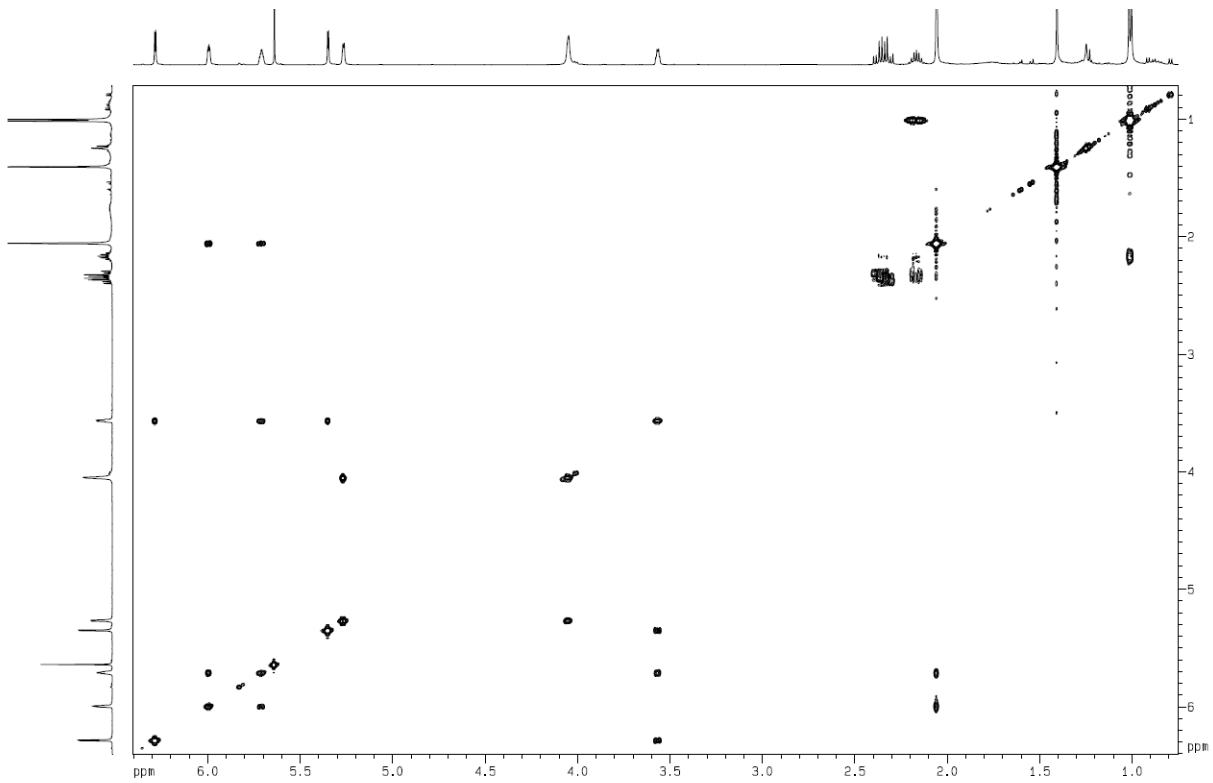

Figure S12. ^{1}H-^{1}H COSY spectrum of **3**

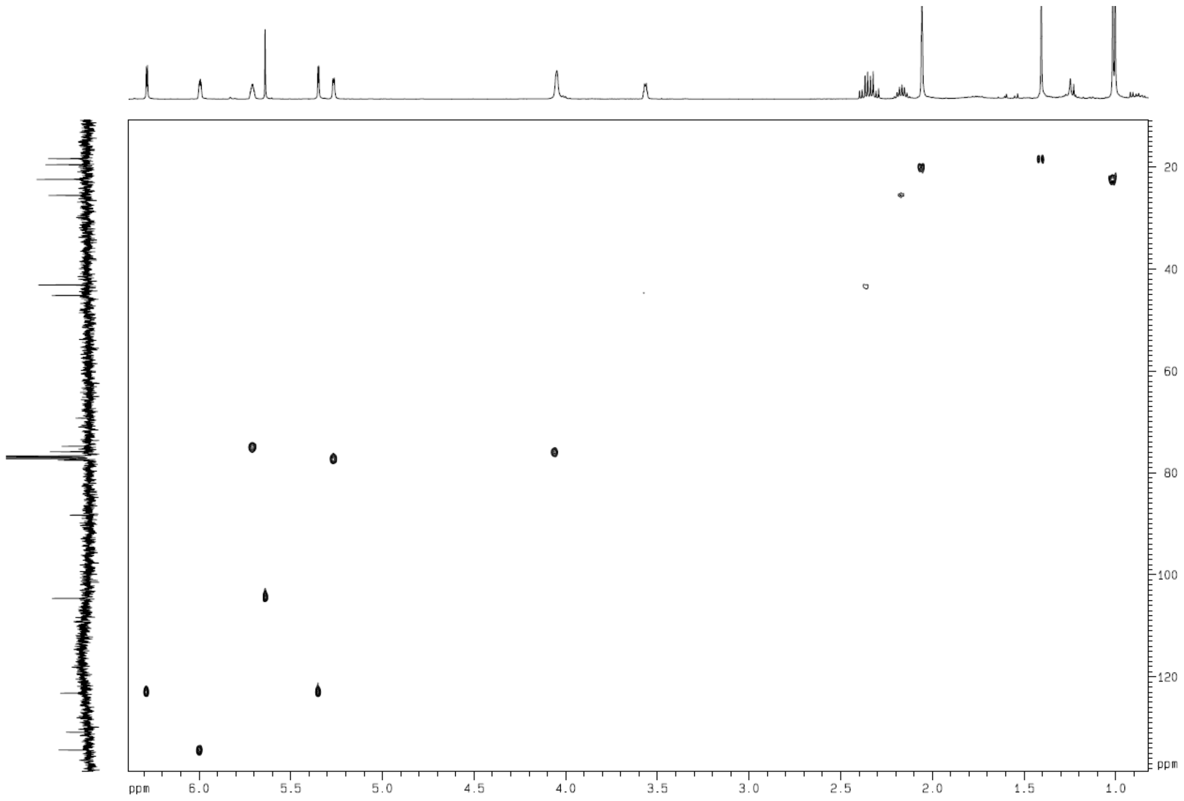

Figure S13. HSQC spectrum of **3**

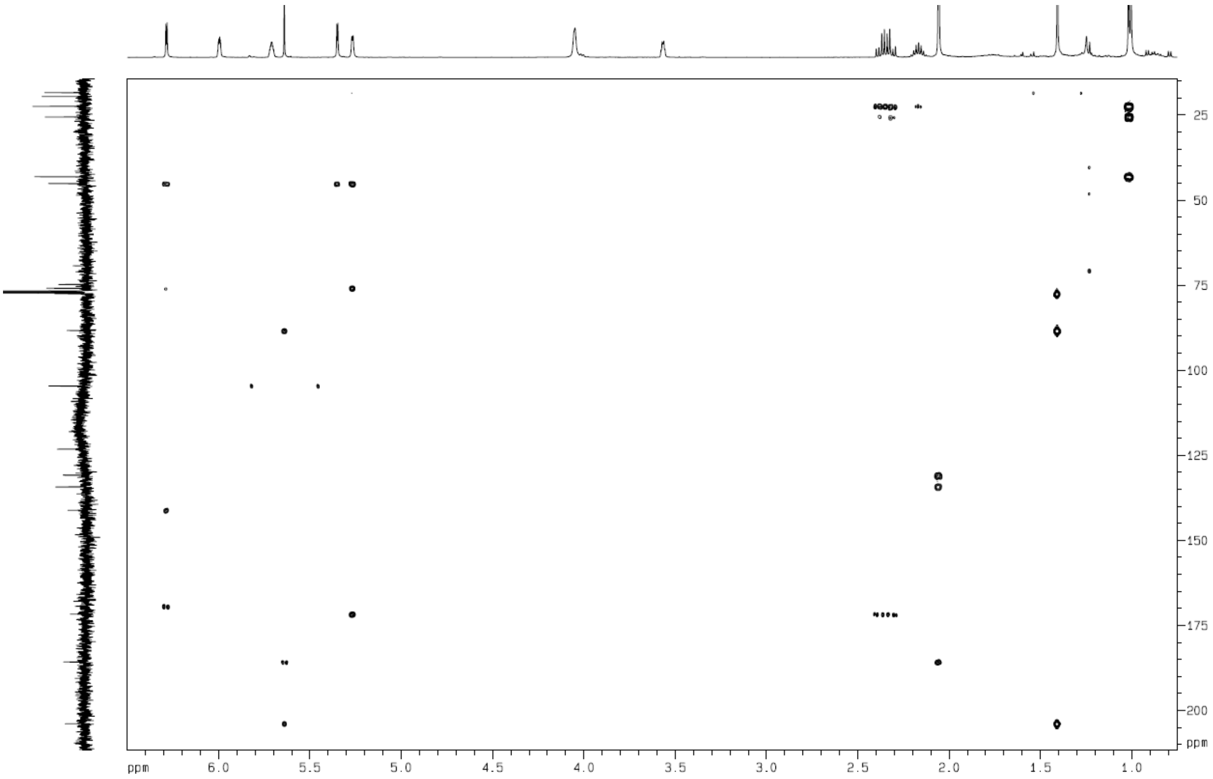

Figure S14. HMBC spectrum of **3**

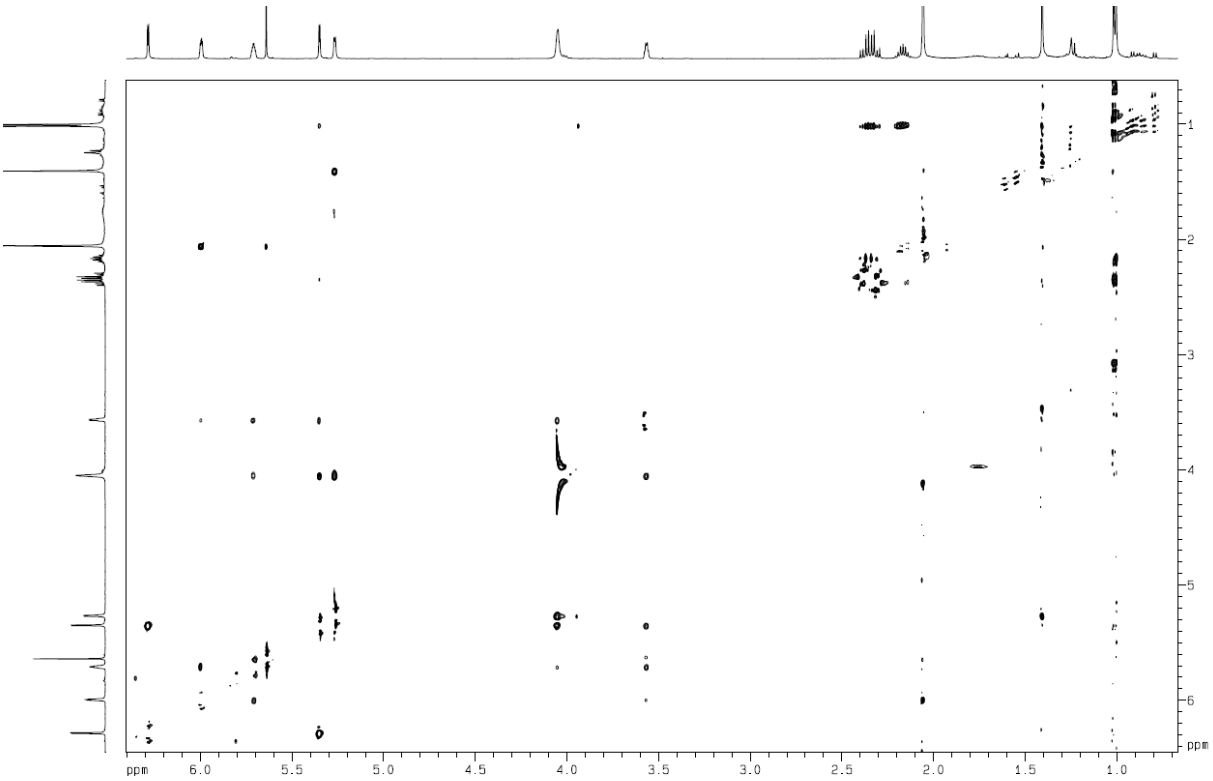

Figure S15. NOESY spectrum of **3**

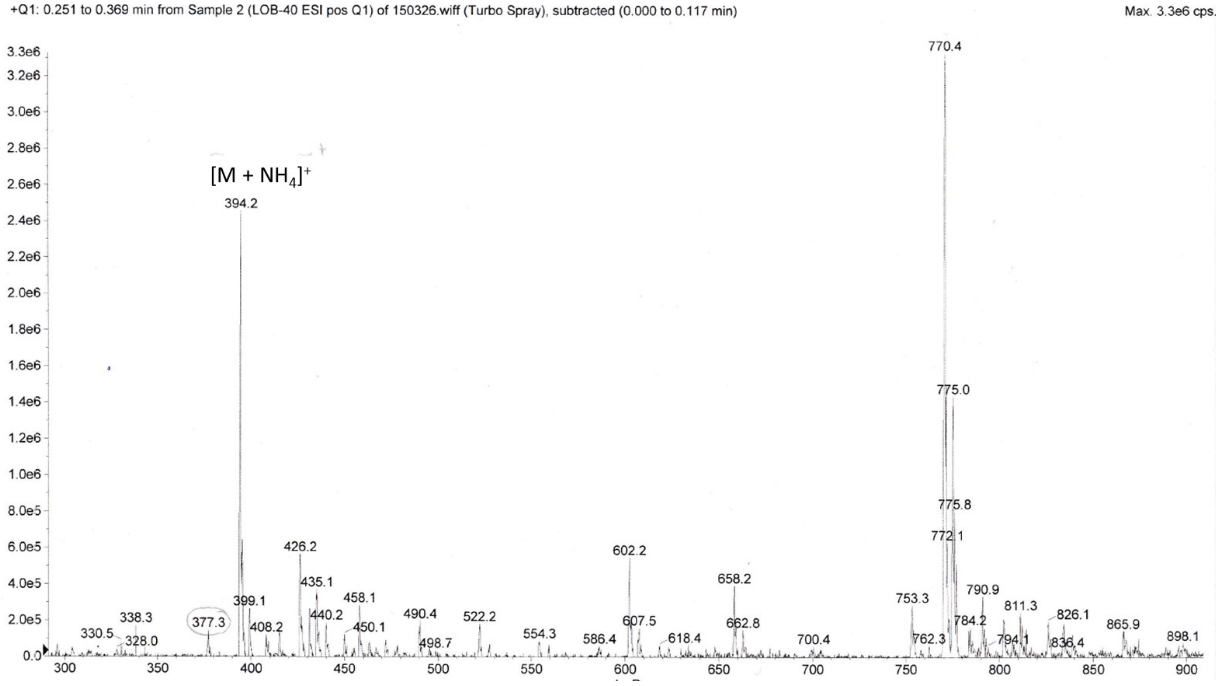

Figure S16. HRMS spectrum of **3**

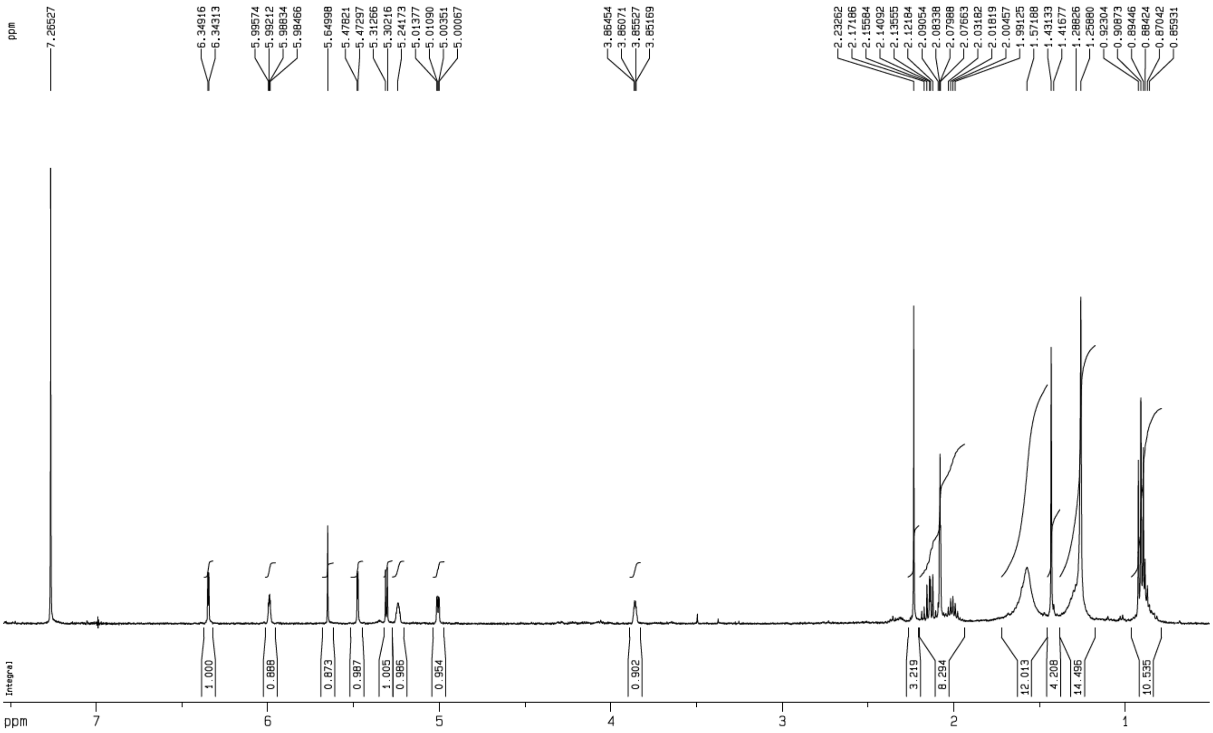

Figure S17. ^{1}H NMR spectrum of **4**

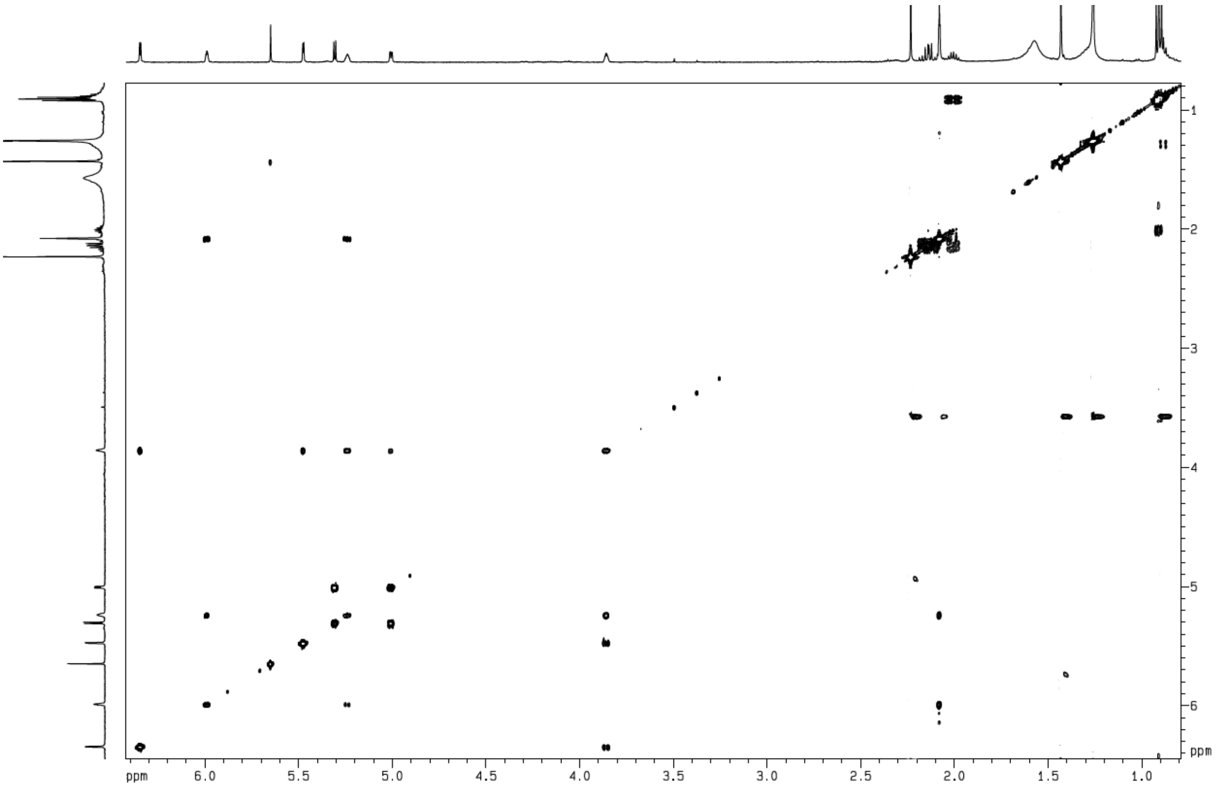

Figure S18. ^1H-^1H COSY spectrum of **4**

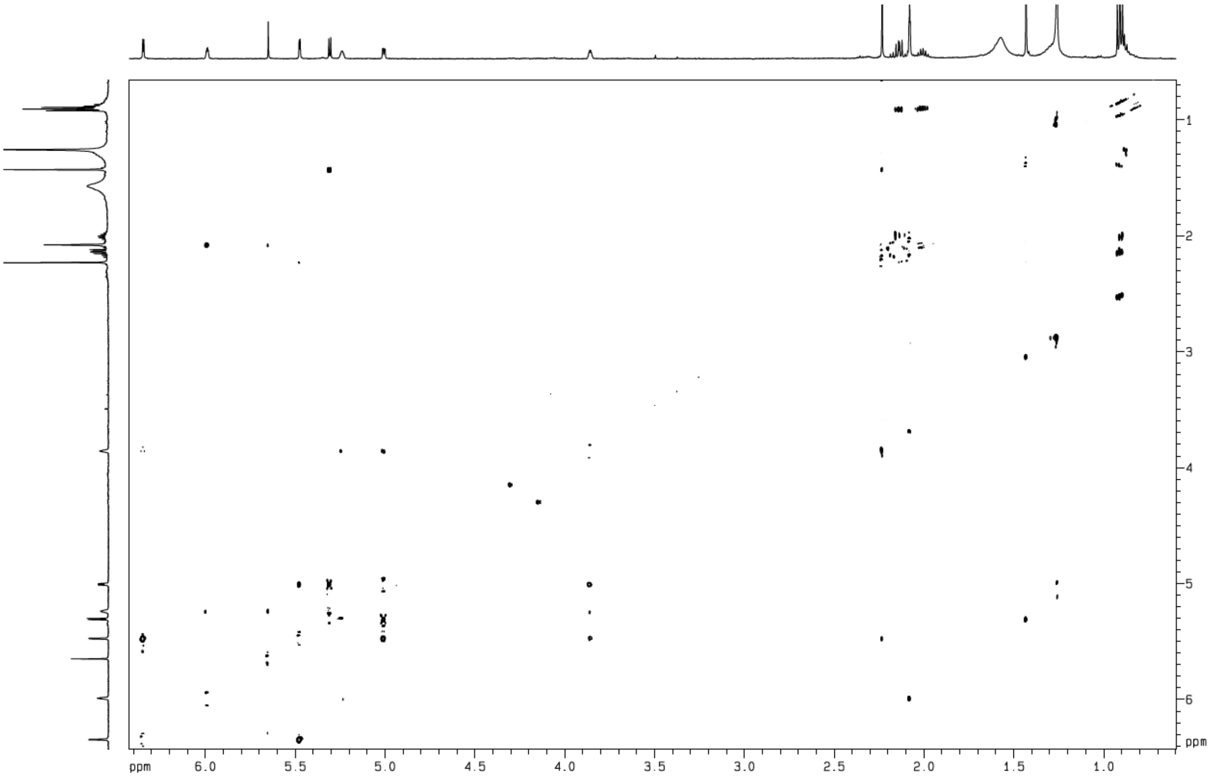

Figure S19. NOESY spectrum of **4**

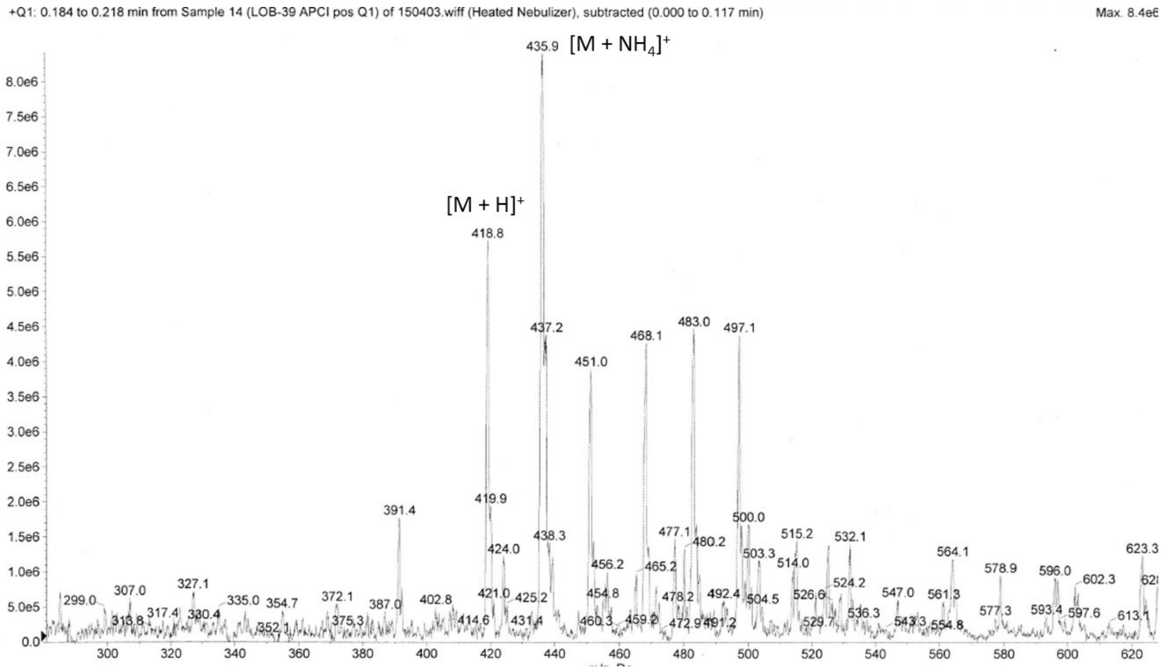

Figure S20. HRMS spectrum of 4

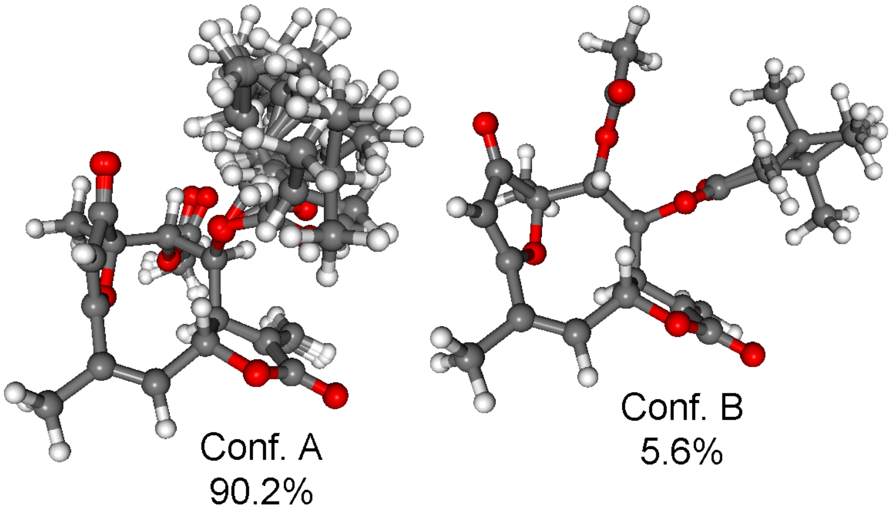

Figure S21. Overlapped geometries of the conformers of the two conformer groups of (6*R*,7*S*,8*S*,9*R*,10*R*)-**4**. Group A: confs. A-F, I, J; group B: confs. G, H. Level of optimization: CAM-B3LYP/TZVP PCM/MeCN.

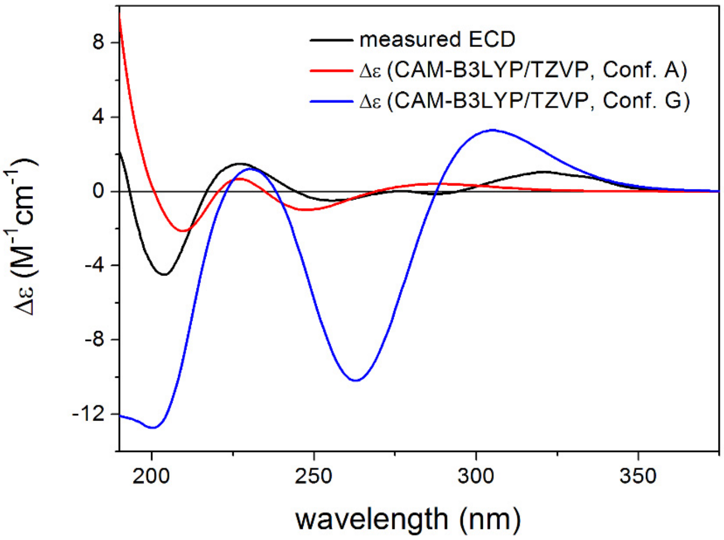

Figure S22. Comparison of the experimental ECD spectrum of **4** measured in MeCN with the CAM-B3LYP/TZVP PCM/MeCN spectra of conformers A and G of (6*R*,7*S*,8*S*,9*R*,10*R*)-**4**, as the lowest-energy representatives of groups A and B. Level of optimization: CAM-B3LYP/TZVP PCM/MeCN.

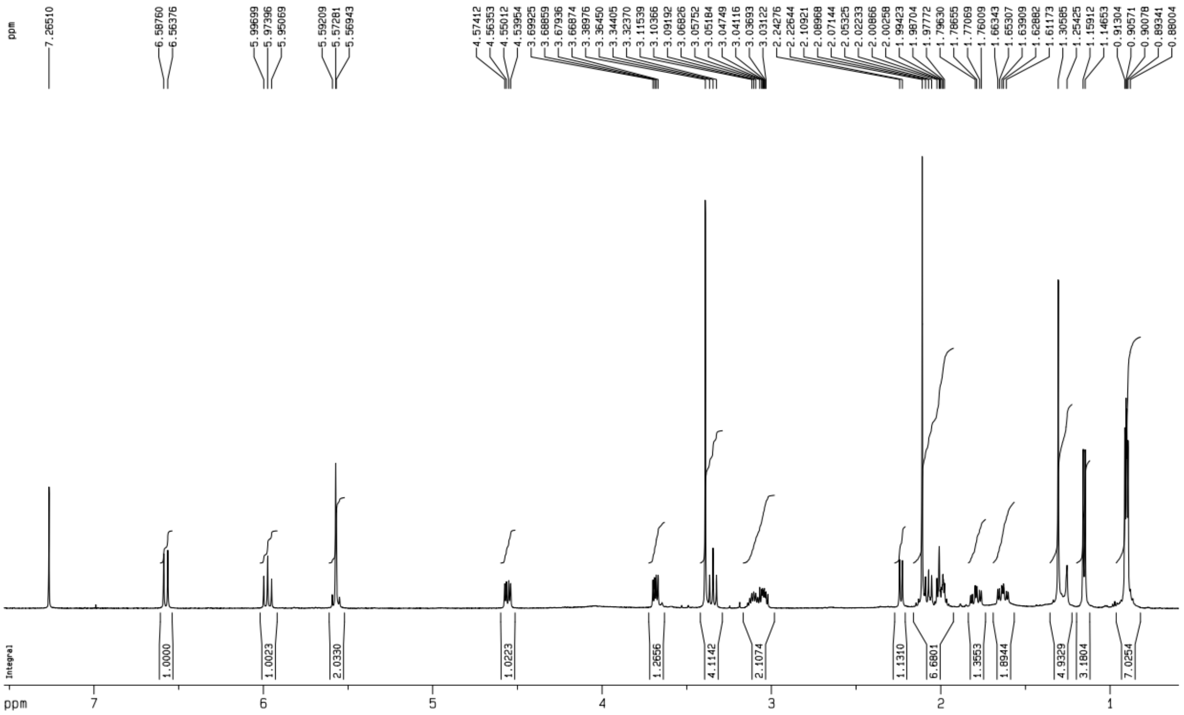

Figure S23. ^{1}H NMR spectrum of **5**

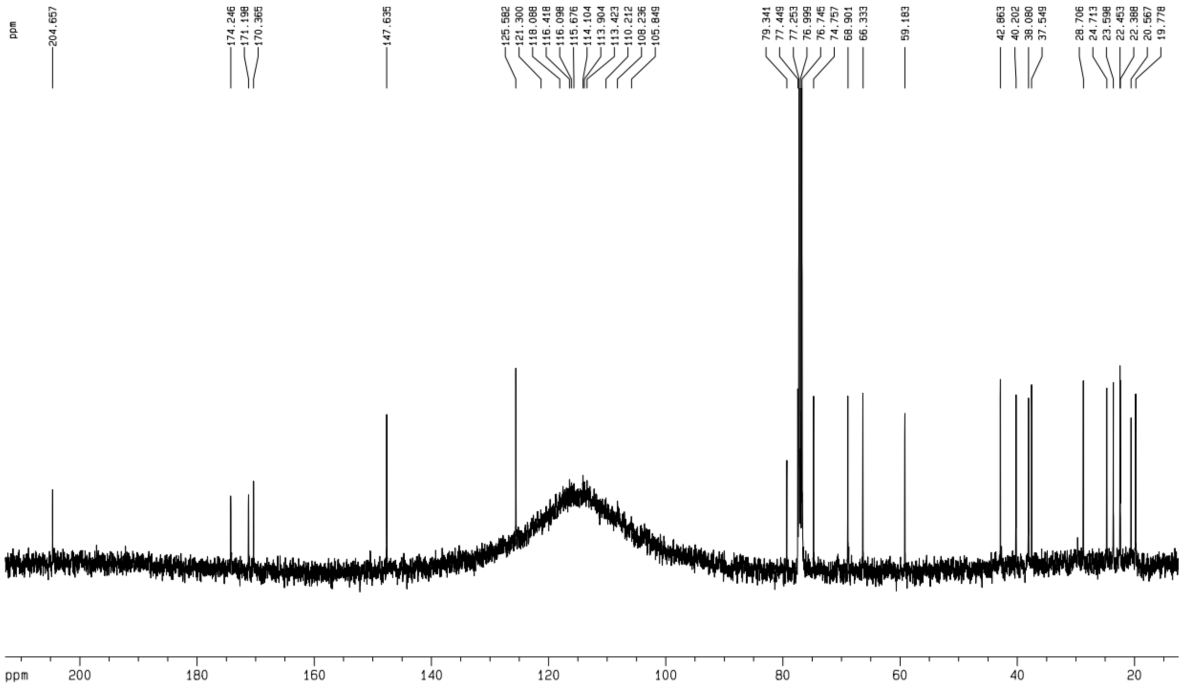

Figure S24. JMOD spectrum of **5**

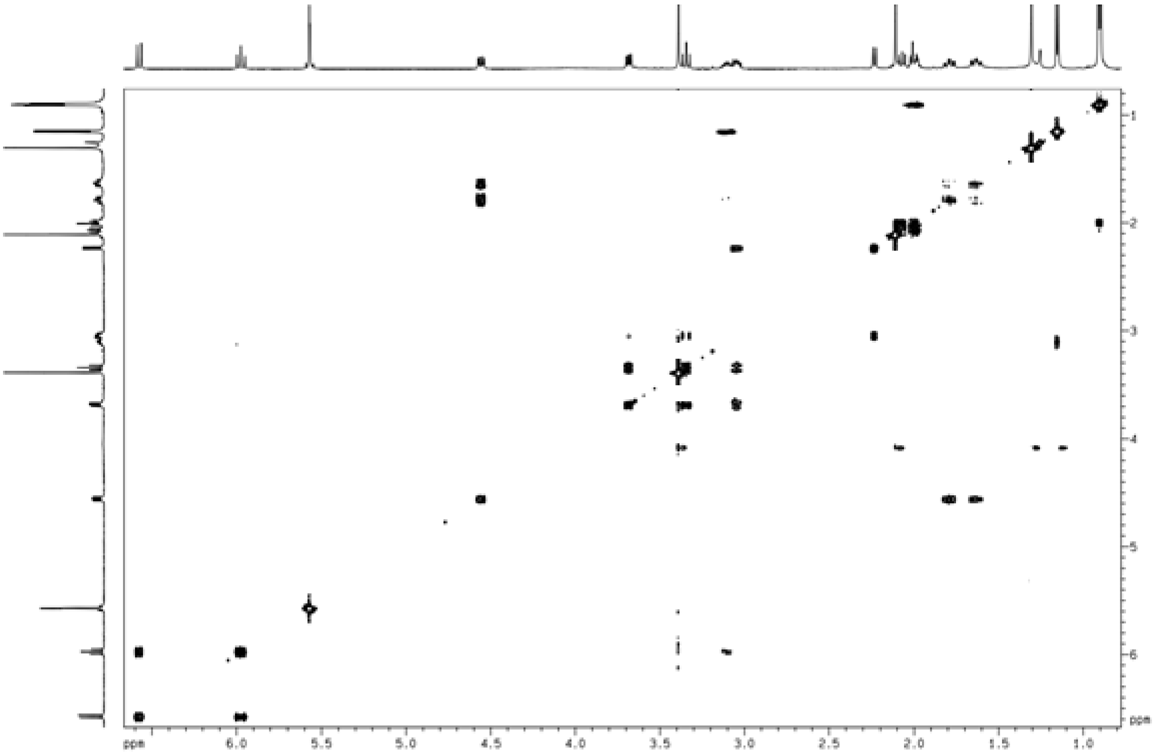

Figure S25. ^{1}H-^{1}H COSY spectrum of **5**

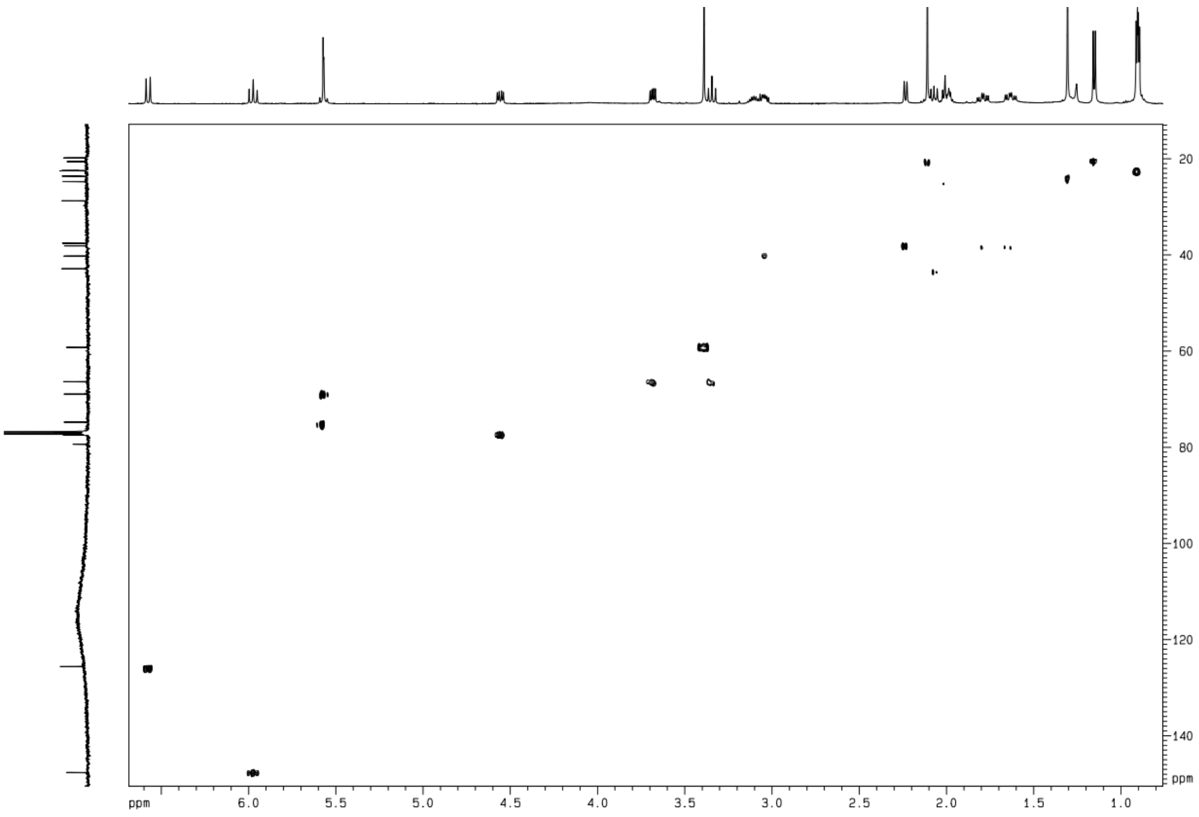

Figure S26. HSQC spectrum of **5**

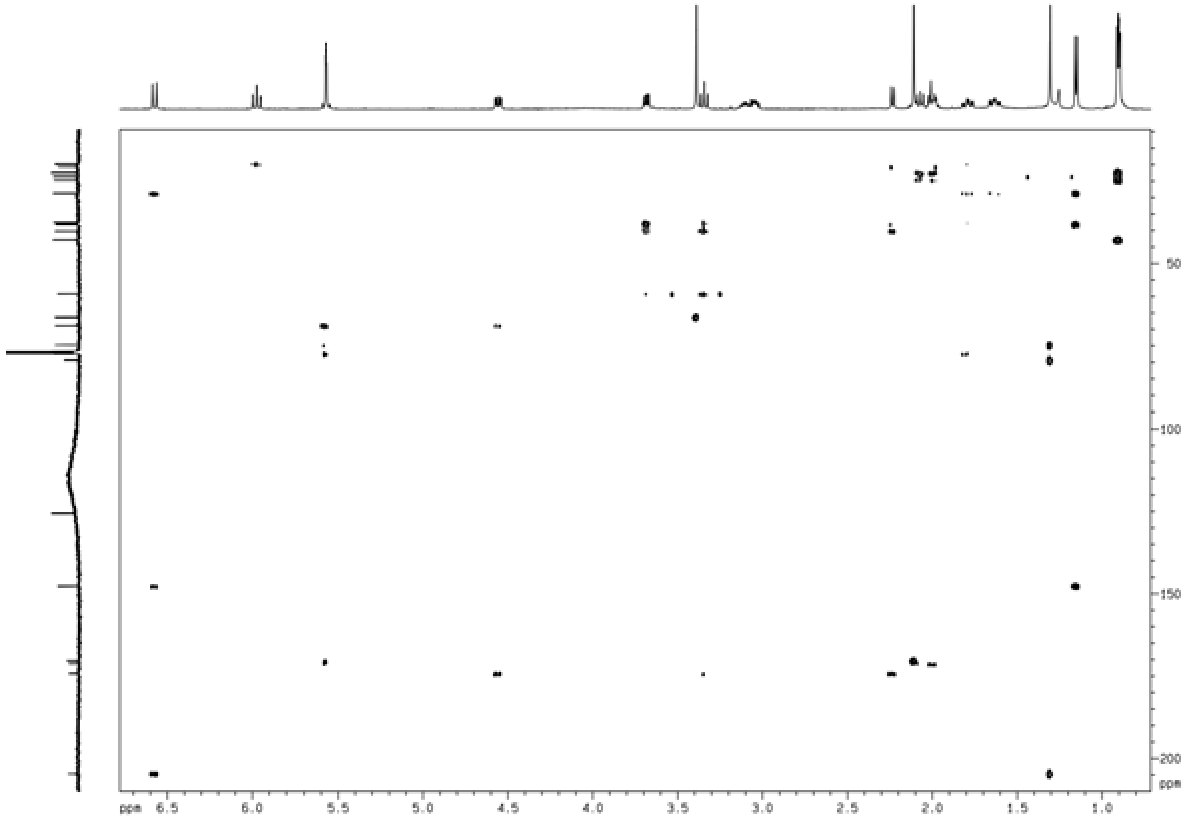

Figure S27. HMBC spectrum of **5**

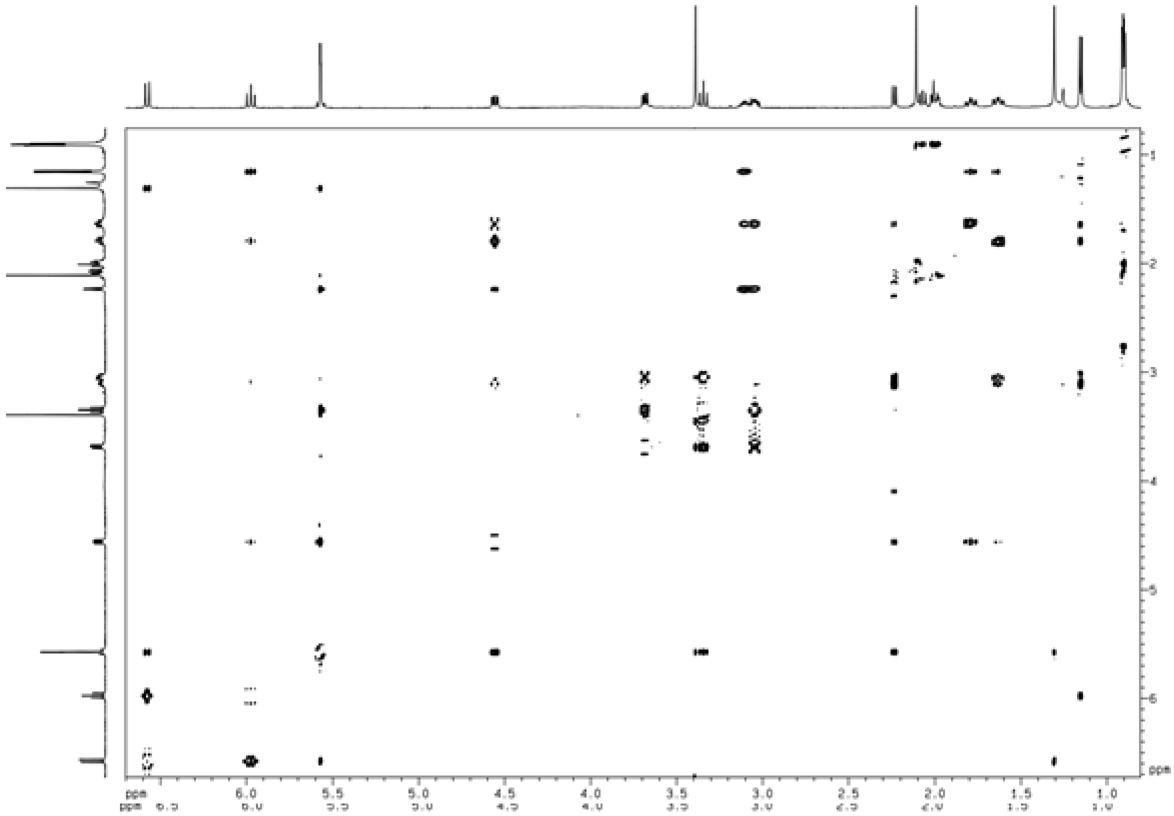

Figure S28. NOESY spectrum of **5**

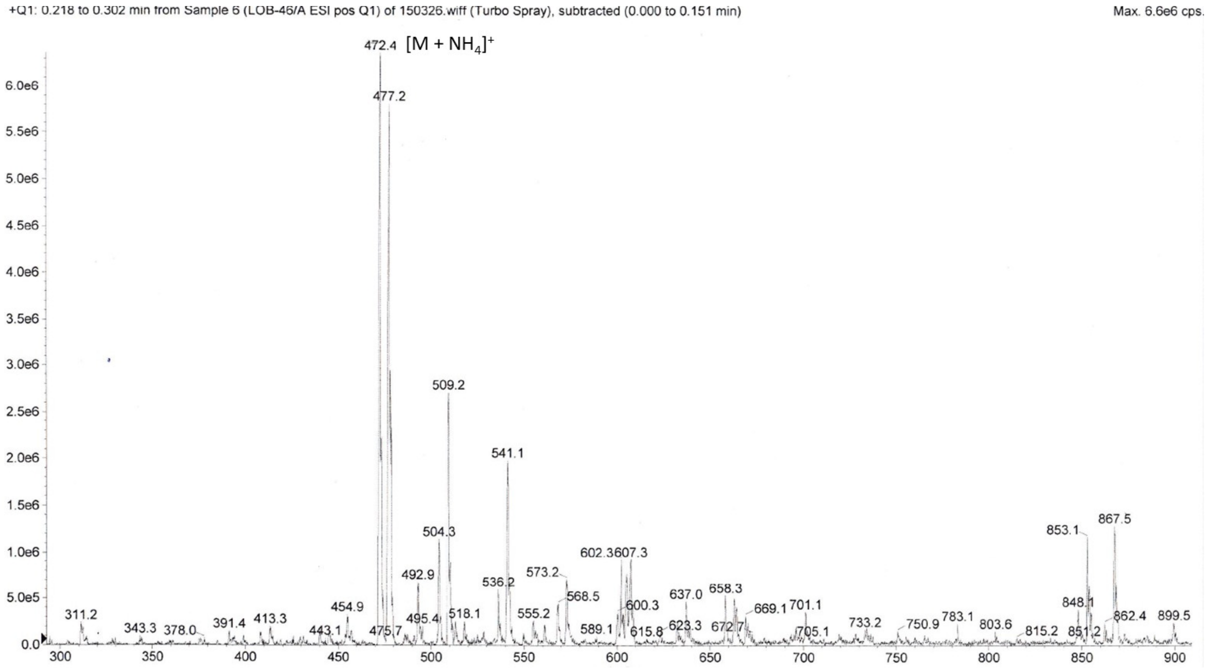

Figure S29. HRMS spectrum of **5**

Table S5. Comparison of the experimental ^{13}C NMR data of all carbons of **5** with the mPW1PW91/6-311+G(2d,p) // B3LYP/6-31+G(d,p) ones of the 1: (4*R*,6*R*,7*S*,8*S*,9*R*,10*R*,11*S*)-**5**, 2: (4*R*,6*R*,7*S*,8*S*,9*R*,10*R*,11*R*)-**5**, 3: (4*R*,6*R*,7*S*,8*R*,9*R*,10*R*,11*S*)-**5**, 4: (4*R*,6*R*,7*S*,8*R*,9*R*,10*R*,11*R*)-**5**, 5: (4*R*,6*R*,7*S*,8*R*,9*S*,10*R*,11*S*)-**5**, 6: (4*R*,6*R*,7*S*,8*R*,9*S*,10*R*,11*R*)-**5**, 7: (4*R*,6*R*,7*S*,8*S*,9*S*,10*R*,11*S*)-**5** and the 8: (4*R*,6*R*,7*S*,8*S*,9*S*,10*R*,11*R*)-**5** stereoisomers. For better comparison, Δδ values over 2.5 were highlighted with yellow and those over 5.0 with red.

Numbering	Exp	$Calc_1$	$Calc_2$	$Calc_3$	$Calc_4$	$Calc_5$	$Calc_6$	$Calc_7$	$Calc_8$	$\Delta\delta_1$	$\Delta\delta_2$	$\Delta\delta_3$	$\Delta\delta_4$	$\Delta\delta_5$	$\Delta\delta_6$	$\Delta\delta_7$	$\Delta\delta_8$
C-1	204.7	208.73	208.50	208.24	208.04	209.38	209.19	208.86	208.44	4.03	3.80	3.54	3.34	4.68	4.49	4.16	3.74
C-2	125.6	127.84	126.69	126.75	125.97	127.01	126.96	129.03	127.89	2.24	1.09	1.15	0.37	1.41	1.36	3.43	2.29
C-3	147.6	152.98	154.52	156.00	157.96	156.56	158.23	151.47	153.28	5.38	6.92	8.40	10.36	8.96	10.63	3.87	5.68
C-4	28.7	30.88	30.33	30.27	30.82	31.81	31.56	30.81	30.25	2.18	1.63	1.57	2.12	3.11	2.86	2.11	1.55
C-5	38.1	38.46	39.94	38.83	41.15	39.26	42.15	39.58	41.42	0.36	1.84	0.73	3.05	1.16	4.05	1.48	3.32
C-6	77.4	76.85	77.45	76.35	81.05	82.23	81.52	76.72	77.55	0.55	0.05	1.05	3.65	4.83	4.12	0.68	0.15
C-7	37.5	40.10	40.65	42.77	39.95	40.36	42.61	40.08	40.84	2.60	3.15	5.27	2.45	2.86	5.11	2.58	3.34
C-8	68.9	68.92	73.87	72.74	75.06	71.48	71.03	67.86	71.46	0.02	4.97	3.84	6.16	2.58	2.13	1.04	2.56
C-9	74.8	75.41	74.89	73.81	78.07	78.70	80.09	77.54	77.57	0.61	0.09	0.99	3.27	3.90	5.29	2.74	2.77
C-10	79.3	81.22	80.97	82.46	80.14	82.35	81.49	79.56	79.31	1.92	1.67	3.16	0.84	3.05	2.19	0.26	0.01
C-11	40.2	42.54	47.77	44.63	46.15	43.97	43.46	42.41	47.70	2.34	7.57	4.43	5.95	3.77	3.26	2.21	7.50
C-12	174.2	173.91	176.00	173.24	175.95	174.14	176.72	174.06	175.76	0.29	1.80	0.96	1.75	0.06	2.52	0.14	1.56
C-13	66.3	65.71	70.36	67.02	70.77	66.42	71.90	65.96	70.41	0.59	4.06	0.72	4.47	0.12	5.60	0.34	4.11
C-14	23.6	22.46	22.53	22.22	22.03	18.20	16.24	23.41	23.32	1.14	1.07	1.38	1.57	5.40	7.36	0.19	0.28
C-15	19.8	18.71	18.78	18.59	18.45	18.35	18.40	18.82	18.88	1.09	1.02	1.21	1.35	1.45	1.40	0.98	0.92
OMe	59.2	56.83	56.56	56.48	56.61	56.82	56.59	56.81	56.57	2.37	2.64	2.72	2.59	2.38	2.61	2.39	2.63
ival CO 1'	171.2	173.21	174.25	174.55	174.51	174.11	174.08	173.39	173.60	2.01	3.05	3.35	3.31	2.91	2.88	2.19	2.40
C-2'	42.9	42.02	41.92	42.06	42.48	41.86	42.40	41.63	41.50	0.88	0.98	0.84	0.42	1.04	0.50	1.27	1.40
C-3'	24.7	26.82	26.94	26.71	27.53	27.12	27.72	26.82	26.86	2.12	2.24	2.01	2.83	2.42	3.02	2.12	2.16
C-4'	22.4	20.58	20.52	20.74	20.44	20.43	20.34	20.35	20.22	1.82	1.88	1.66	1.96	1.97	2.06	2.05	2.18
C-5'	22.5	20.64	20.64	20.97	20.54	20.52	20.56	20.61	20.64	1.86	1.86	1.53	1.96	1.98	1.94	1.89	1.86
Ac CO	170.4	172.34	171.98	171.90	170.93	170.53	171.85	170.80	170.93	1.94	1.58	1.50	0.53	0.13	1.45	0.40	0.53
Ac CH_3	20.6	19.76	19.79	19.77	19.88	19.92	19.72	20.25	20.27	0.84	0.81	0.83	0.72	0.68	0.88	0.35	0.33
CMAE	N/A	N/A	N/A	N/A	N/A	N/A	N/A	N/A	N/A	1.70	2.43	2.30	2.83	2.65	3.38	1.69	2.32

Table S6. Comparison of the experimental ^{13}C NMR data of all carbons of **5** with the mPW1PW91/6-311+G(2d,p) SMD/CDCl3 // B3LYP/6-31+G(d,p) ones of the 1: (4*R*,6*R*,7*S*,8*S*,9*R*,10*R*,11*S*)-**5**, 2: (4*R*,6*R*,7*S*,8*S*,9*R*,10*R*,11*R*)-**5**, 3: (4*R*,6*R*,7*S*,8*R*,9*R*,10*R*,11*S*)-**5**, 4: (4*R*,6*R*,7*S*,8*R*,9*R*,10*R*,11*R*)-**5**, 5: (4*R*,6*R*,7*S*,8*R*,9*S*,10*R*,11*S*)-**5**, 6: (4*R*,6*R*,7*S*,8*R*,9*S*,10*R*,11*R*)-**5**, 7: (4*R*,6*R*,7*S*,8*S*,9*S*,10*R*,11*S*)-**5** and the 8: (4*R*,6*R*,7*S*,8*S*,9*S*,10*R*,11*R*)-**5** stereoisomers. For better comparison, Δδ values over 2.5 were highlighted with yellow and those over 5.0 with red.

Numbering	Exp	$Calc_1$	$Calc_2$	$Calc_3$	$Calc_4$	$Calc_5$	$Calc_6$	$Calc_7$	$Calc_8$	$\Delta\delta_1$	$\Delta\delta_2$	$\Delta\delta_3$	$\Delta\delta_4$	$\Delta\delta_5$	$\Delta\delta_6$	$\Delta\delta_7$	$\Delta\delta_8$
C-1	204.7	206.10	206.00	206.18	205.84	207.04	206.37	206.57	206.04	1.40	1.30	1.48	1.14	2.34	1.67	1.87	1.34
C-2	125.6	125.83	124.92	124.97	124.66	125.33	125.25	126.85	125.79	0.23	0.68	0.63	0.94	0.27	0.35	1.25	0.19
C-3	147.6	153.02	153.78	155.70	157.35	157.22	158.04	151.47	153.00	5.42	6.18	8.10	9.75	9.62	10.44	3.87	5.40
C-4	28.7	31.60	31.21	31.14	31.67	32.96	32.66	31.65	31.34	2.90	2.51	2.44	2.97	4.26	3.96	2.95	2.64
C-5	38.1	37.96	39.69	38.48	40.89	38.99	41.77	38.89	40.87	0.14	1.59	0.38	2.79	0.89	3.67	0.79	2.77
C-6	77.4	77.12	77.51	76.62	80.71	82.08	81.13	77.09	77.81	0.28	0.11	0.78	3.31	4.68	3.73	0.31	0.41
C-7	37.5	40.46	40.92	42.90	40.24	40.36	42.41	40.41	41.04	2.96	3.42	5.40	2.74	2.86	4.91	2.91	3.54
C-8	68.9	68.62	73.62	72.55	74.46	71.02	70.29	67.77	71.36	0.28	4.72	3.65	5.56	2.12	1.39	1.13	2.46
C-9	74.8	75.30	74.63	73.68	77.71	77.98	78.68	76.75	76.53	0.50	0.17	1.12	2.91	3.18	3.88	1.95	1.73
C-10	79.3	80.73	80.07	81.82	79.51	81.60	80.77	78.82	78.63	1.43	0.77	2.52	0.21	2.30	1.47	0.48	0.67
C-11	40.2	42.62	48.37	45.22	46.70	44.15	44.20	42.49	48.22	2.42	8.17	5.02	6.50	3.95	4.00	2.29	8.02
C-12	174.2	174.29	176.98	174.47	177.19	174.53	177.59	174.78	177.11	0.09	2.78	0.27	2.99	0.33	3.39	0.58	2.91
C-13	66.3	65.17	69.64	66.53	70.01	65.95	71.13	65.38	69.62	1.13	3.34	0.23	3.71	0.35	4.83	0.92	3.32
C-14	23.6	22.86	22.86	22.70	22.62	18.45	16.82	23.64	23.60	0.74	0.74	0.90	0.98	5.15	6.78	0.04	0.00
C-15	19.8	18.58	18.93	18.42	18.51	18.50	18.58	18.66	18.96	1.22	0.87	1.38	1.29	1.30	1.22	1.14	0.84
OMe	59.2	56.47	55.96	56.07	56.10	56.34	56.14	56.40	56.03	2.73	3.24	3.13	3.10	2.86	3.06	2.80	3.17
ival CO 1'	171.2	171.98	173.13	172.96	173.29	172.93	172.88	172.23	172.55	0.78	1.93	1.76	2.09	1.73	1.68	1.03	1.35
C-2'	42.9	42.04	42.14	42.11	42.58	42.04	42.44	41.88	41.83	0.86	0.76	0.79	0.32	0.86	0.46	1.02	1.07
C-3'	24.7	27.50	27.71	27.23	28.28	27.80	28.62	27.26	27.23	2.80	3.01	2.53	3.58	3.10	3.92	2.56	2.53
C-4'	22.4	20.41	20.35	20.53	20.21	20.23	20.22	20.15	20.06	1.99	2.05	1.87	2.19	2.17	2.18	2.25	2.34
C-5'	22.5	20.49	20.44	20.75	20.37	20.38	20.35	20.34	20.44	2.01	2.06	1.75	2.13	2.12	2.15	2.16	2.06
Ac CO	170.4	171.76	171.54	171.59	171.10	170.30	171.55	170.63	170.79	1.36	1.14	1.19	0.70	0.10	1.15	0.23	0.39
Ac CH3	20.6	20.52	20.59	20.54	20.62	20.56	20.44	20.96	21.01	0.08	0.01	0.06	0.02	0.04	0.16	0.36	0.41
CMAE	N/A	N/A	N/A	N/A	N/A	N/A	N/A	N/A	N/A	1.47	2.24	2.06	2.69	2.46	3.06	1.52	2.15

Table S7. Comparison of the experimental ^{1}H NMR data of all hydrogens except for the OH hydrogen of **5** with the mPW1PW91/6-311+G(2d,p) // B3LYP/6-31+G(d,p) ones of the 1: (4*R*,6*R*,7*S*,8*S*,9*R*,10*R*,11*S*)-**5**, 2: (4*R*,6*R*,7*S*,8*S*,9*R*,10*R*,11*R*)-**5**, 3: (4*R*,6*R*,7*S*,8*R*,9*R*,10*R*,11*S*)-**5**, 4: (4*R*,6*R*,7*S*,8*R*,9*R*,10*R*,11*R*)-**5**, 5: (4*R*,6*R*,7*S*,8*R*,9*S*,10*R*,11*S*)-**5**, 6: (4*R*,6*R*,7*S*,8*R*,9*S*,10*R*,11*R*)-**5**, 7: (4*R*,6*R*,7*S*,8*S*,9*S*,10*R*,11*S*)-**5** and the 8: (4*R*,6*R*,7*S*,8*S*,9*S*,10*R*,11*R*)-**5** stereoisomers. Shielding constants related to the hydrogen atoms in the methyl groups were averaged. For better comparison, Δδ values over 0.3 were highlighted with yellow and those over 0.6 with red.

Numbering	Exp	$Calc_1$	$Calc_2$	$Calc_3$	$Calc_4$	$Calc_5$	$Calc_6$	$Calc_7$	$Calc_8$	$Δδ_1$	$Δδ_2$	$Δδ_3$	$Δδ_4$	$Δδ_5$	$Δδ_6$	$Δδ_7$	$Δδ_8$
H-2	6.58	6.64	6.60	6.58	6.56	6.63	6.69	6.50	6.46	0.06	0.02	0.00	0.02	0.05	0.11	0.08	0.12
H-3	5.97	6.01	6.06	6.03	6.11	6.04	6.14	5.91	5.97	0.04	0.09	0.06	0.14	0.07	0.17	0.06	0.00
H-4	3.09	3.35	3.35	3.67	3.62	3.45	3.53	3.45	3.49	0.26	0.26	0.58	0.53	0.36	0.44	0.36	0.40
H-5a	1.79	1.68	1.57	1.56	1.66	1.78	1.76	1.65	1.59	0.11	0.22	0.23	0.13	0.01	0.03	0.14	0.20
H-5b	1.63	1.57	1.99	1.62	2.11	1.66	1.97	1.58	1.97	0.06	0.36	0.01	0.48	0.03	0.34	0.05	0.34
H-6	4.56	4.26	4.22	4.22	3.98	3.97	3.88	4.78	4.71	0.30	0.34	0.34	0.58	0.59	0.68	0.22	0.15
H-7	2.23	2.21	2.14	2.56	2.45	2.31	2.21	2.44	2.39	0.02	0.09	0.33	0.22	0.08	0.02	0.21	0.16
H-8	5.57	5.65	5.63	5.12	5.22	5.29	5.23	5.61	5.62	0.08	0.06	0.45	0.35	0.28	0.34	0.04	0.05
H-9	5.57	5.58	5.60	5.26	5.24	5.26	4.39	5.60	5.46	0.01	0.03	0.31	0.33	0.31	1.18	0.03	0.11
H-11	3.04	2.84	2.55	2.85	2.48	2.77	3.01	2.83	2.12	0.20	0.49	0.19	0.56	0.27	0.03	0.21	0.92
H-13a	3.68	3.54	3.60	3.43	3.70	3.87	3.56	3.54	3.55	0.14	0.08	0.25	0.02	0.19	0.12	0.14	0.13
H-13b	3.34	3.46	3.35	3.39	3.43	3.64	3.79	3.03	3.35	0.12	0.01	0.05	0.09	0.30	0.45	0.31	0.01
H-14	1.31	1.26	1.25	1.34	1.53	1.50	1.63	1.34	1.34	0.05	0.06	0.03	0.22	0.19	0.32	0.03	0.03
H-15	1.15	1.15	1.12	1.16	1.13	1.16	1.14	1.16	1.13	0.00	0.03	0.01	0.02	0.01	0.01	0.01	0.02
OMe	3.39	3.15	3.39	3.34	3.40	3.62	3.42	3.24	3.24	0.24	0.00	0.05	0.01	0.23	0.03	0.15	0.15
H-2a'	2.00	2.17	2.21	2.39	2.24	1.99	2.06	2.00	2.04	0.17	0.21	0.39	0.24	0.01	0.06	0.00	0.04
H-2b'	2.00	1.95	1.97	2.24	2.16	2.19	2.20	2.04	2.09	0.05	0.03	0.24	0.16	0.19	0.20	0.04	0.09
H-3'	2.07	2.09	2.13	2.26	2.24	2.16	2.20	2.14	2.14	0.02	0.06	0.19	0.17	0.09	0.13	0.07	0.07
H-4'	0.91	0.97	0.95	1.05	1.05	0.95	0.97	0.97	0.95	0.06	0.04	0.14	0.14	0.04	0.06	0.06	0.04
H-5'	0.89	0.93	0.93	1.03	1.04	0.95	0.96	0.92	0.92	0.04	0.04	0.14	0.15	0.06	0.07	0.03	0.03
OAc	2.11	2.04	2.03	2.07	2.06	2.03	2.04	2.10	2.11	0.07	0.08	0.04	0.05	0.08	0.07	0.01	0.00
H-2	6.58	6.64	6.60	6.58	6.56	6.63	6.69	6.50	6.46	0.06	0.02	0.00	0.02	0.05	0.11	0.08	0.12
CMAE	N/A	N/A	N/A	N/A	N/A	N/A	N/A	N/A	N/A	0.08	0.11	0.16	0.19	0.14	0.19	0.08	0.11

Table S8. Comparison of the experimental ^{13}C NMR data of all carbons of **5** with the mPW1PW91/6-311+G(2d,p) SMD/$CDCl_3$ // B3LYP/6-31+G(d,p) ones of the 1: (4*R*,6*R*,7*S*,8*S*,9*R*,10*R*,11*S*)-**5**, 2: (4*R*,6*R*,7*S*,8*S*,9*R*,10*R*,11*R*)-**5**, 3: (4*R*,6*R*,7*S*,8*R*,9*R*,10*R*,11*S*)-**5**, 4: (4*R*,6*R*,7*S*,8*R*,9*R*,10*R*,11*R*)-**5**, 5: (4*R*,6*R*,7*S*,8*R*,9*S*,10*R*,11*S*)-**5**, 6: (4*R*,6*R*,7*S*,8*R*,9*S*,10*R*,11*R*)-**5**, 7: (4*R*,6*R*,7*S*,8*S*,9*S*,10*R*,11*S*)-**5** and the 8: (4*R*,6*R*,7*S*,8*S*,9*S*,10*R*,11*R*)-**5** stereoisomers. For better comparison, Δδ values over 2.5 were highlighted with yellow and those over 5.0 with red.

Numbering	Exp	Calc$_1$	Calc$_2$	Calc$_3$	Calc$_4$	Calc$_5$	Calc$_6$	Calc$_7$	Calc$_8$	$\Delta\delta_1$	$\Delta\delta_2$	$\Delta\delta_3$	$\Delta\delta_4$	$\Delta\delta_5$	$\Delta\delta_6$	$\Delta\delta_7$	$\Delta\delta_8$
H-2	6.58	6.62	6.61	6.52	6.50	6.55	6.62	6.49	6.55	0.04	0.03	0.06	0.08	0.03	0.04	0.09	0.03
H-3	5.97	6.12	6.15	6.15	6.23	6.17	6.27	6.02	6.02	0.15	0.18	0.18	0.26	0.20	0.30	0.05	0.05
H-4	3.09	3.16	3.15	3.51	3.45	3.36	3.36	3.25	3.28	0.07	0.06	0.42	0.36	0.27	0.27	0.16	0.19
H-5a	1.79	1.67	1.55	1.55	1.69	1.78	1.81	1.65	1.58	0.12	0.24	0.24	0.10	0.01	0.02	0.14	0.21
H-5b	1.63	1.63	1.88	1.67	1.96	1.70	1.80	1.63	1.86	0.00	0.25	0.04	0.33	0.07	0.17	0.00	0.23
H-6	4.56	4.25	4.21	4.16	4.00	3.96	3.92	4.69	4.66	0.31	0.35	0.40	0.56	0.60	0.64	0.13	0.10
H-7	2.23	2.27	2.16	2.54	2.38	2.22	2.17	2.50	2.44	0.04	0.07	0.31	0.15	0.01	0.06	0.27	0.21
H-8	5.57	5.38	5.38	4.96	5.06	5.00	5.00	5.41	5.43	0.19	0.19	0.61	0.51	0.57	0.57	0.16	0.14
H-9	5.57	5.49	5.49	5.15	5.08	5.01	4.24	5.42	5.30	0.08	0.08	0.42	0.49	0.56	1.33	0.15	0.27
H-11	3.04	3.02	2.54	3.01	2.52	2.92	2.95	3.01	2.17	0.02	0.50	0.03	0.52	0.12	0.09	0.03	0.87
H-13a	3.68	3.44	3.51	3.28	3.57	3.73	3.67	3.46	3.47	0.24	0.17	0.40	0.11	0.05	0.01	0.22	0.21
H-13b	3.34	3.33	3.38	3.27	3.45	3.53	3.48	2.95	3.36	0.01	0.04	0.07	0.11	0.19	0.14	0.39	0.02
H-14 (3x)	1.31	1.26	1.24	1.32	1.49	1.45	1.55	1.34	1.32	0.05	0.07	0.01	0.18	0.14	0.24	0.03	0.01
H-15 (3x)	1.15	1.08	1.05	1.09	1.07	1.13	1.08	1.08	1.07	0.07	0.10	0.06	0.08	0.02	0.07	0.07	0.08
OMe (3x)	3.39	3.13	3.34	3.27	3.34	3.48	3.37	3.20	3.16	0.26	0.05	0.12	0.05	0.09	0.02	0.19	0.23
H-2a'	2.00	2.12	2.18	2.37	2.24	2.04	2.12	2.00	2.08	0.12	0.18	0.37	0.24	0.04	0.12	0.00	0.08
H-2b'	2.00	2.00	2.04	2.24	2.19	2.14	2.22	2.08	2.11	0.00	0.04	0.24	0.19	0.14	0.22	0.08	0.11
H-3'	2.07	1.97	1.98	2.11	2.10	1.99	2.04	1.99	1.98	0.10	0.09	0.04	0.03	0.08	0.03	0.08	0.09
H-4' (3x)	0.91	0.91	0.87	0.97	0.91	0.87	0.88	0.89	0.89	0.00	0.04	0.06	0.00	0.04	0.03	0.02	0.02
H-5' (3x)	0.89	0.85	0.84	0.96	0.90	0.86	0.87	0.85	0.84	0.04	0.05	0.07	0.01	0.03	0.02	0.04	0.05
OAc (3x)	2.11	2.06	2.05	2.04	2.08	2.04	2.06	2.13	2.13	0.05	0.06	0.07	0.03	0.07	0.05	0.02	0.02
CMAE	N/A	N/A	N/A	N/A	N/A	N/A	N/A	N/A	N/A	0.08	0.12	0.16	0.17	0.13	0.18	0.09	0.12

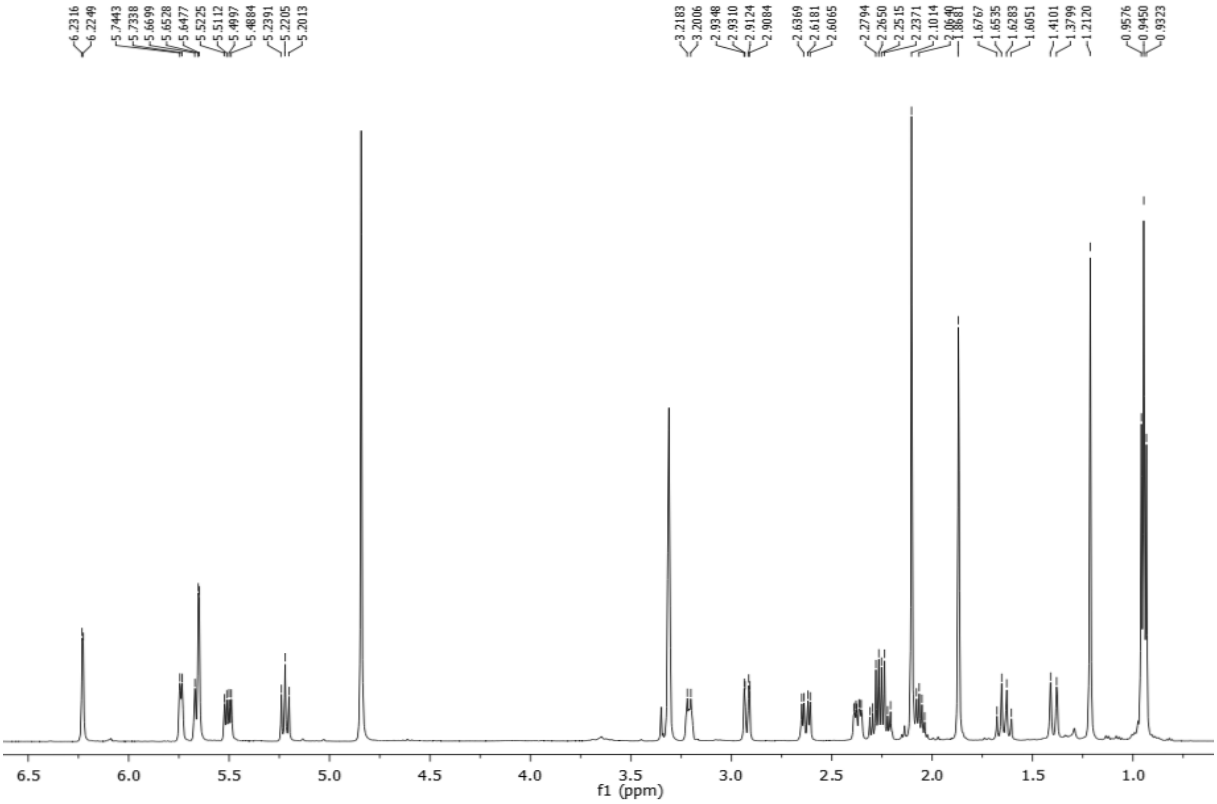

Figure S30. ^{1}H NMR spectrum of **6**

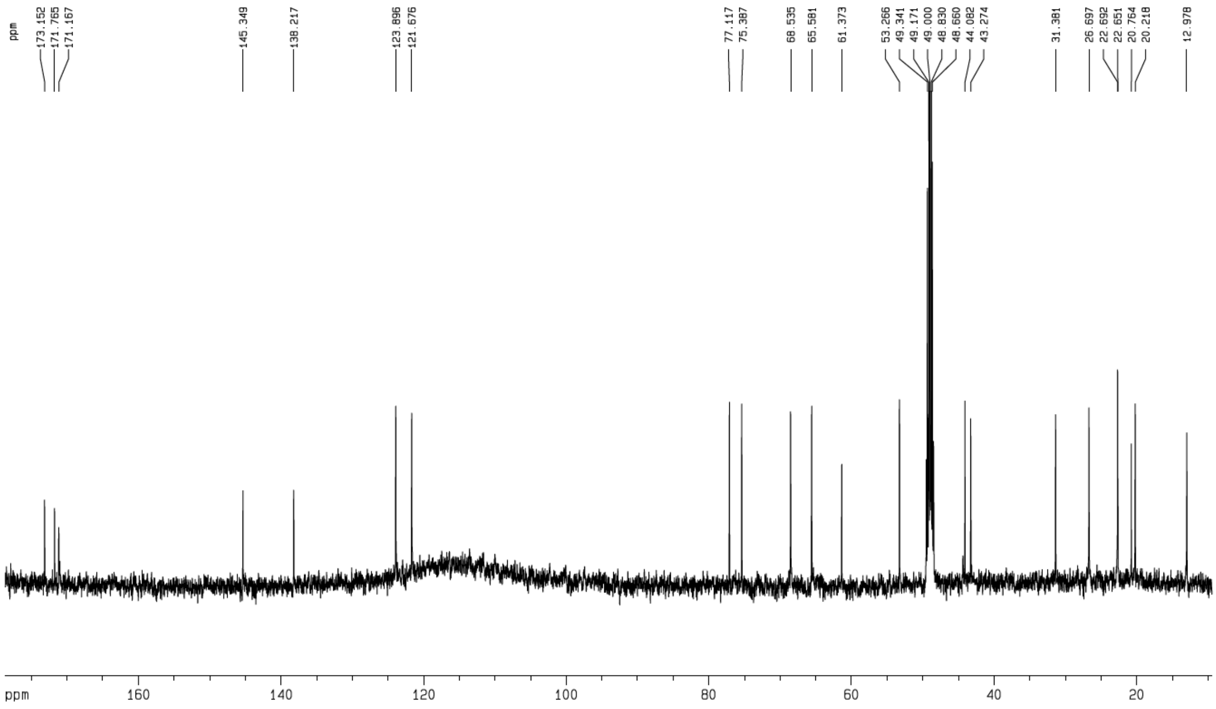

Figure S31. ^{13}C NMR spectrum of **6**

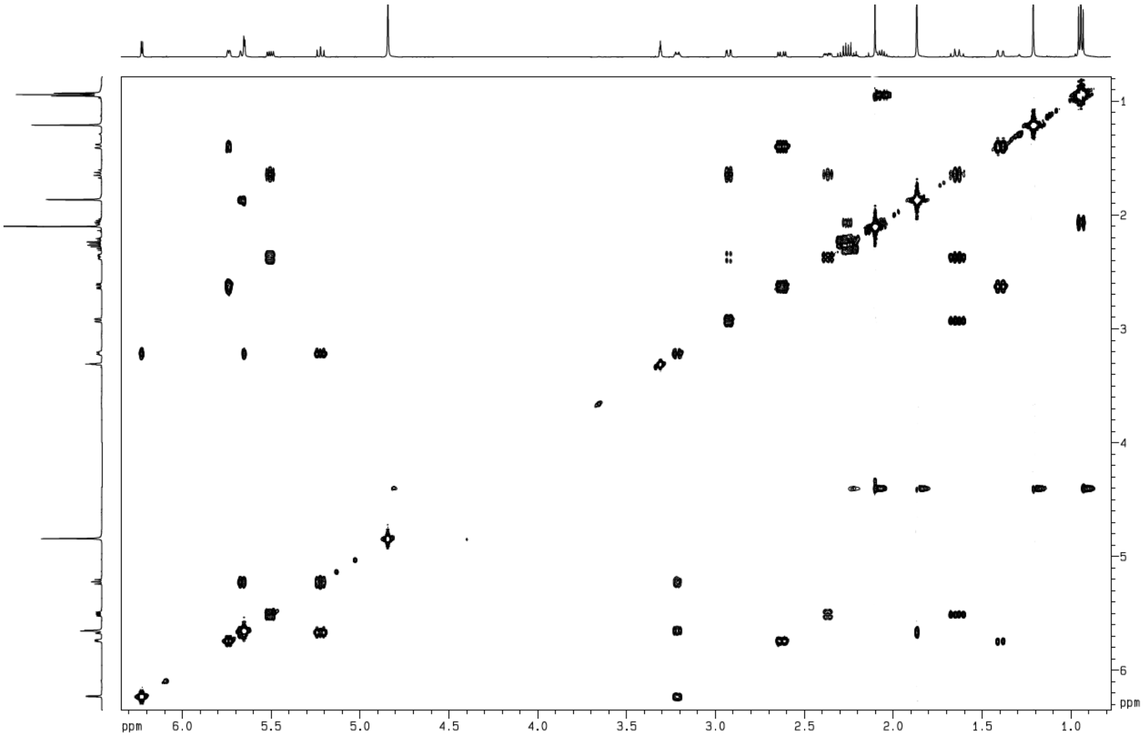

Figure S32. ^{1}H-^{1}H COSY spectrum of **6**

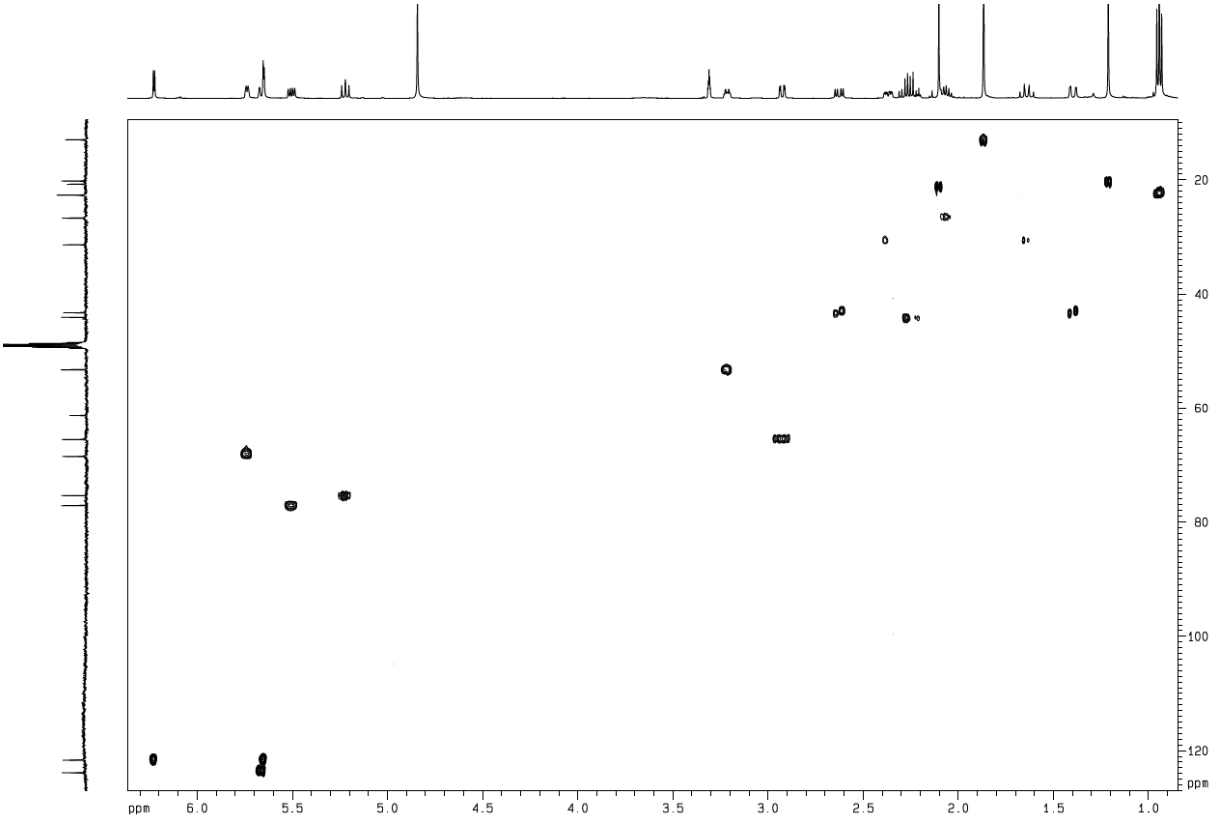

Figure S33. HSQC spectrum of **6**

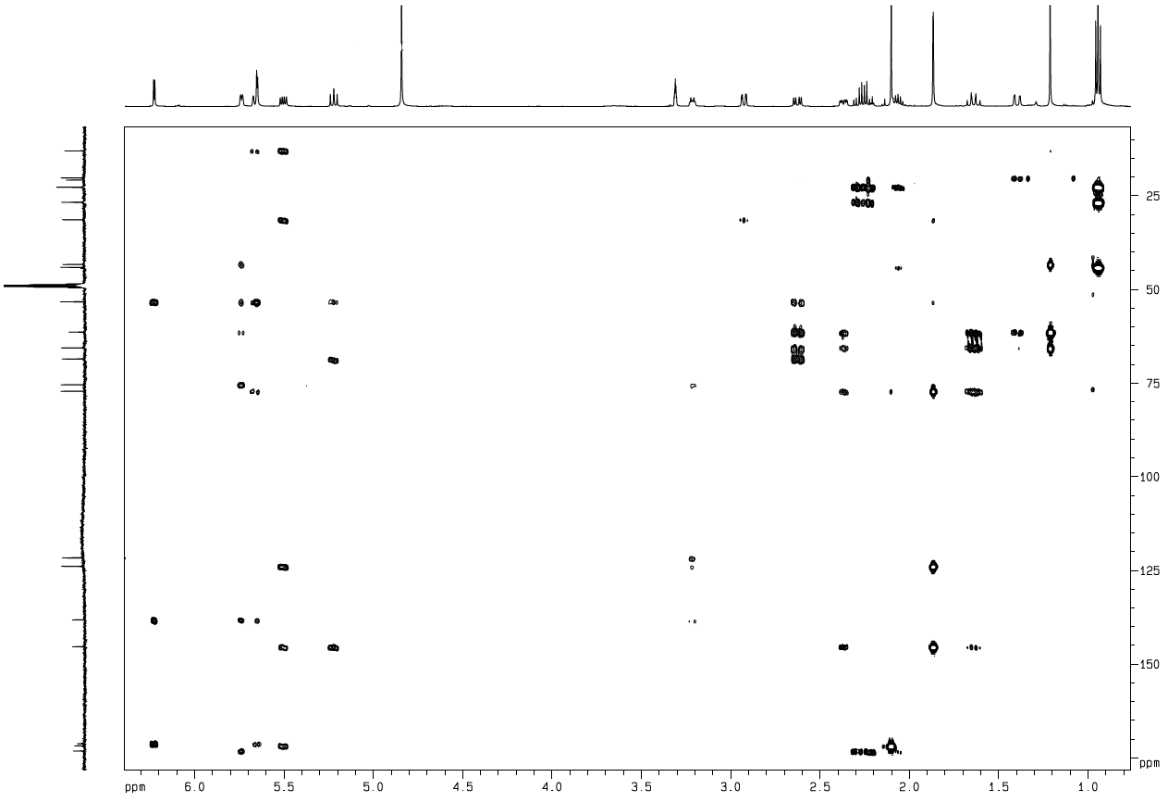

Figure S34. HMBC spectrum of **6**

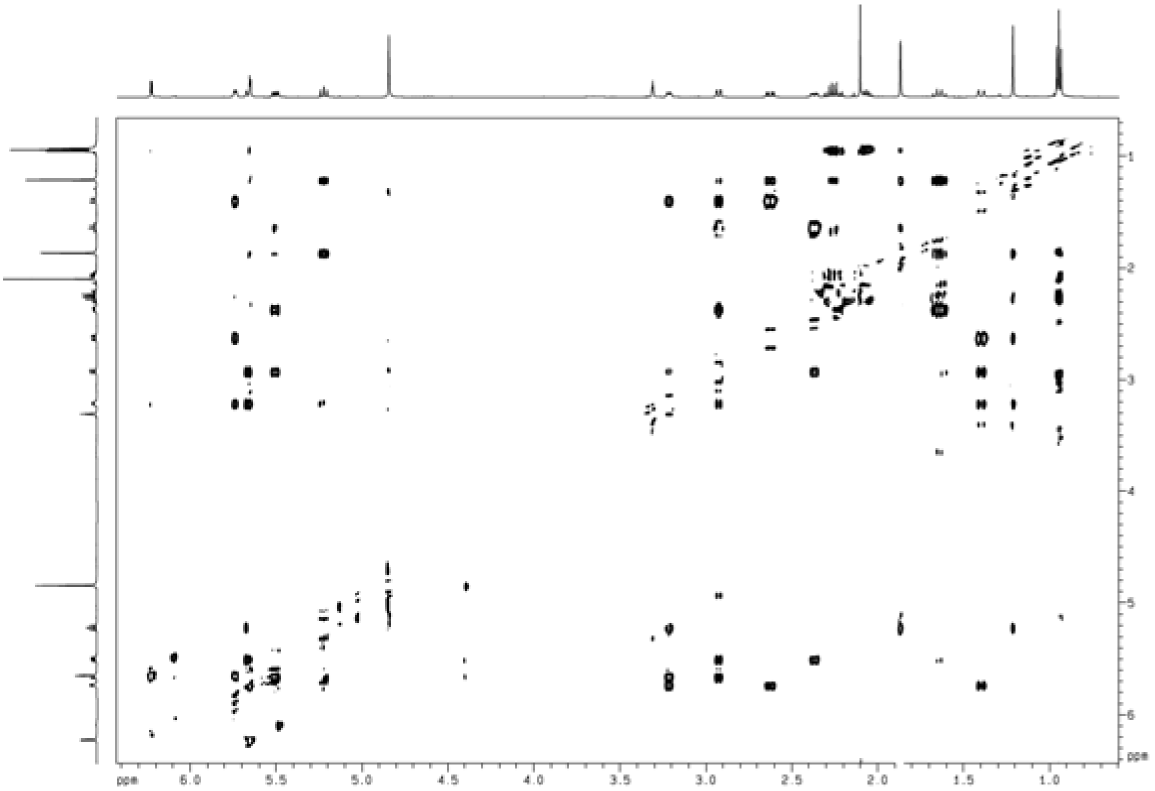

Figure S35. NOESY spectrum of **6**

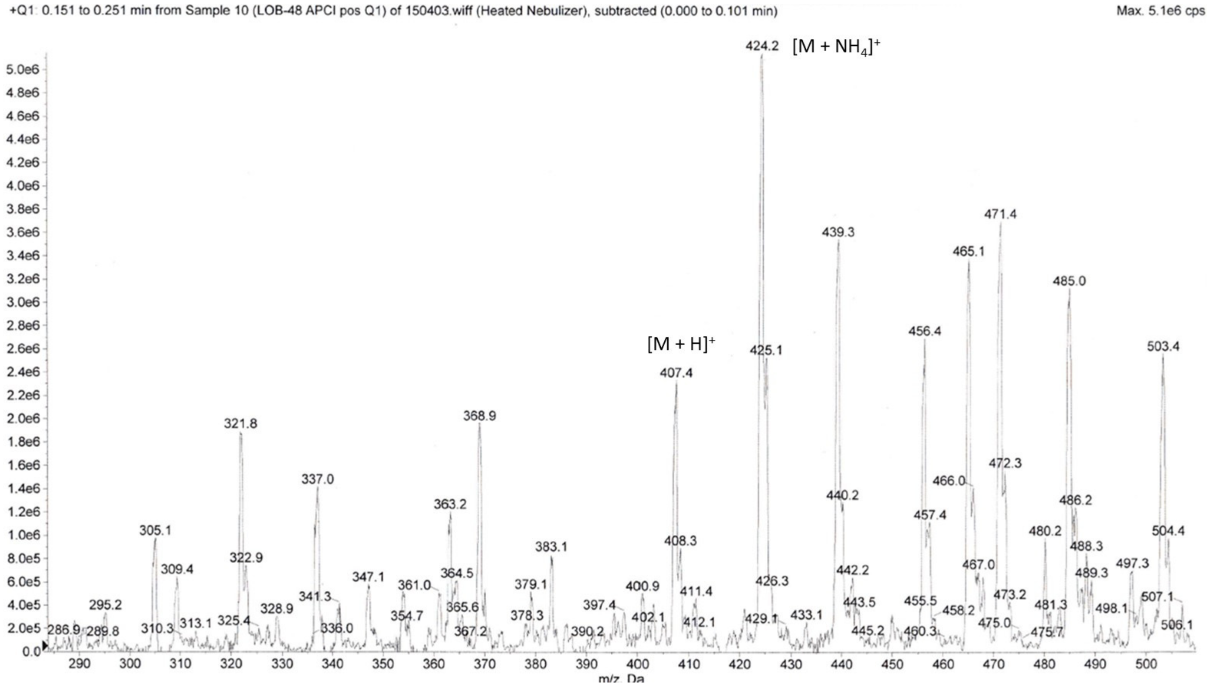

Figure S36. HRMS spectrum of **6**

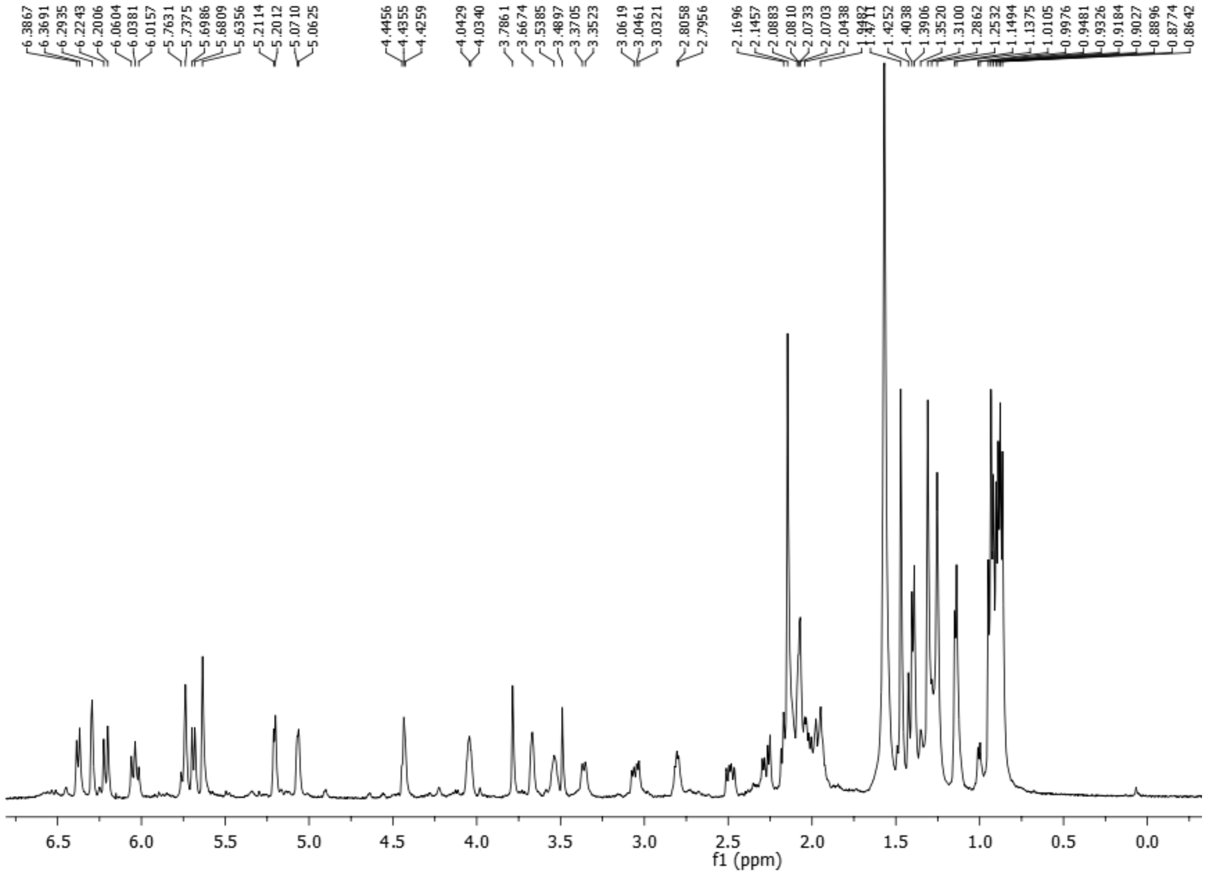

Figure S37. ^{1}H NMR spectrum of **7**

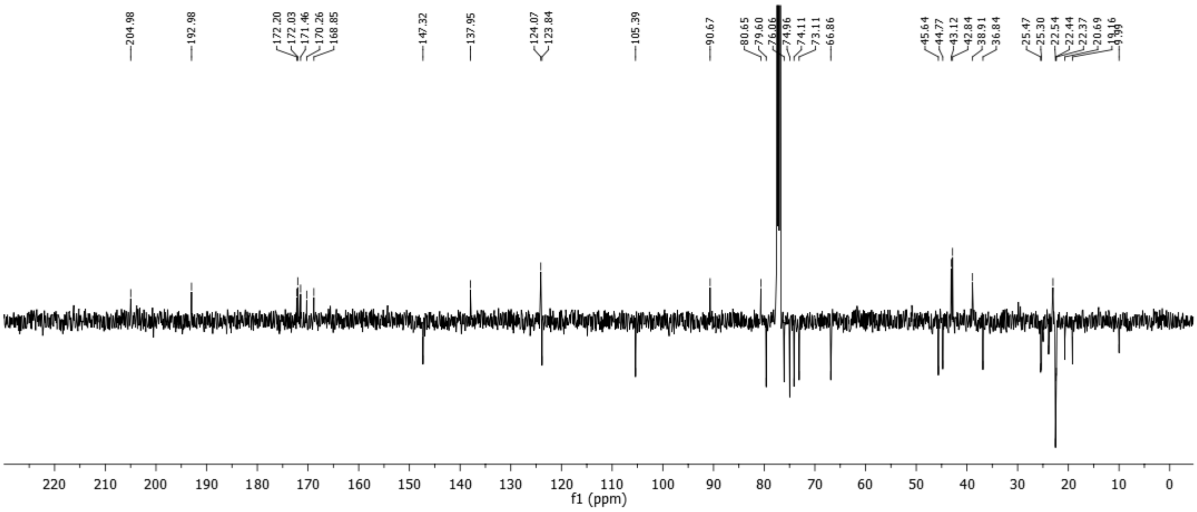

Figure S38. JMOD spectrum of **7**

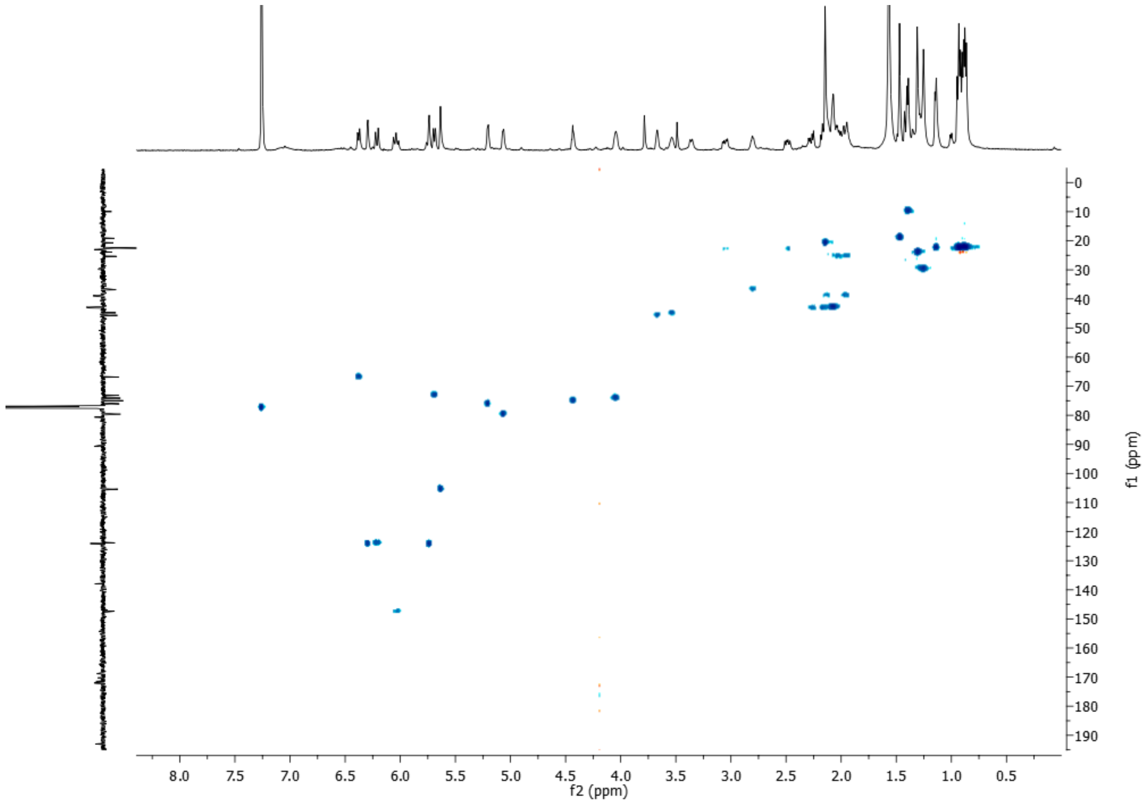

Figure S39. HSQC spectrum of **7**

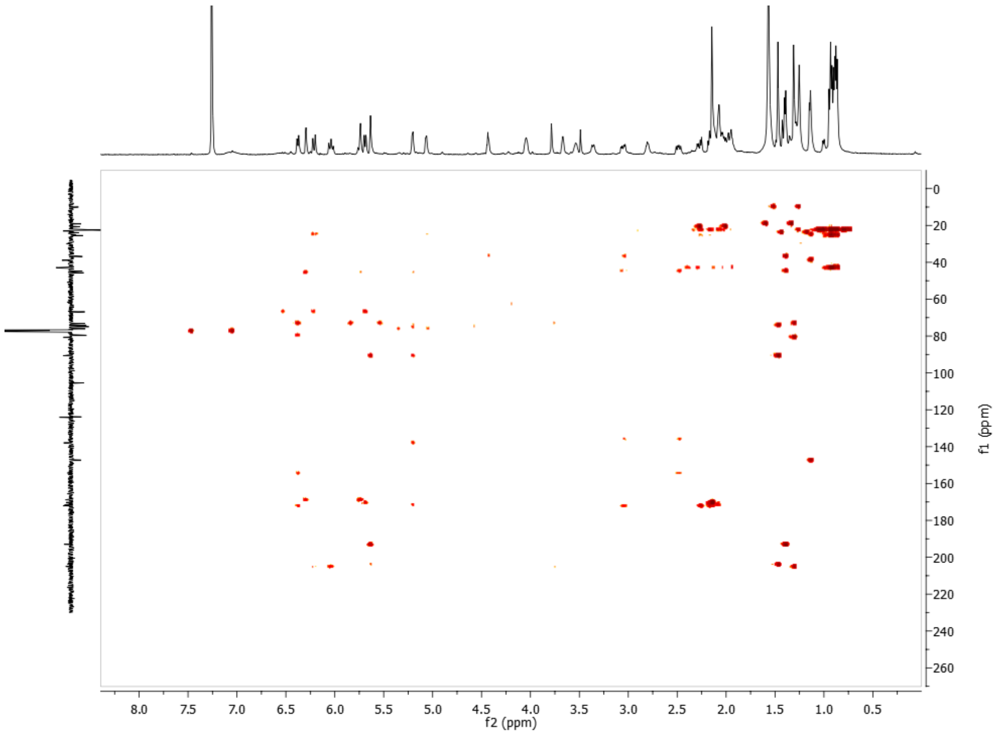

Figure S40. HMBC spectrum of **7**

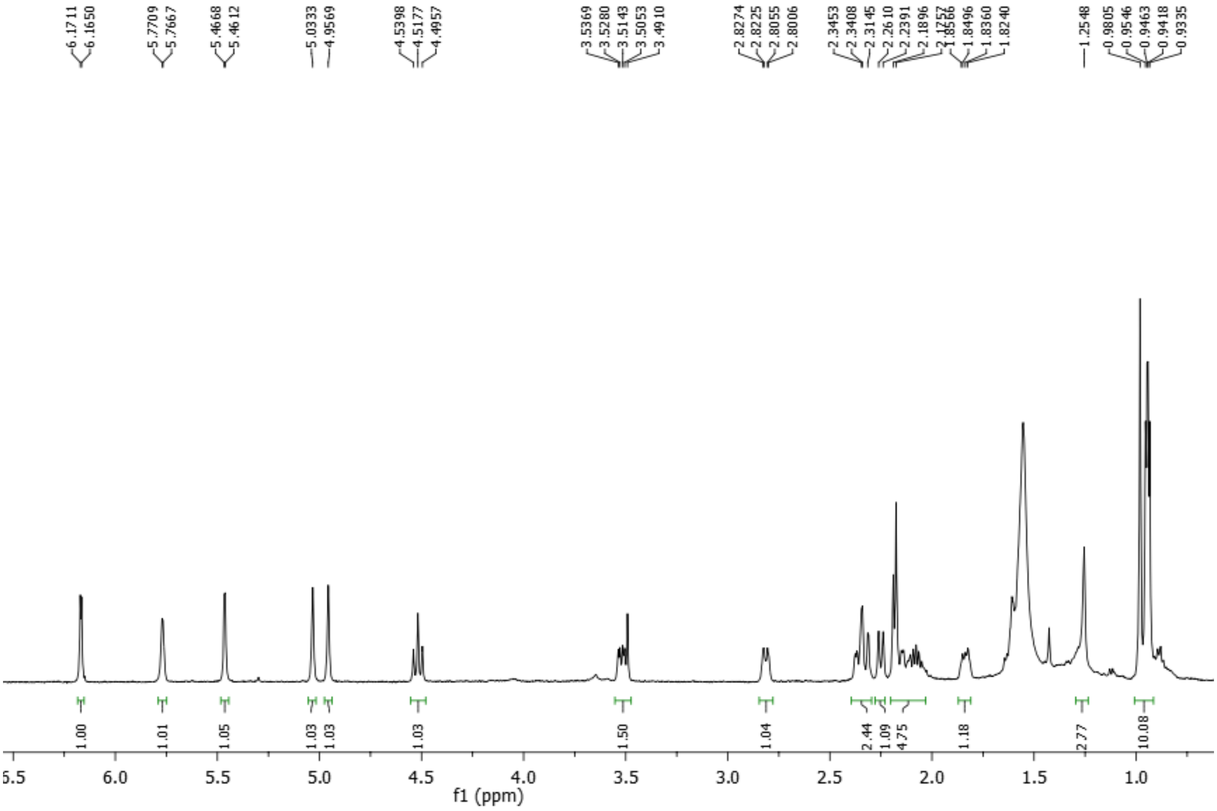

Figure S41. ^{1}H NMR spectrum of **8**

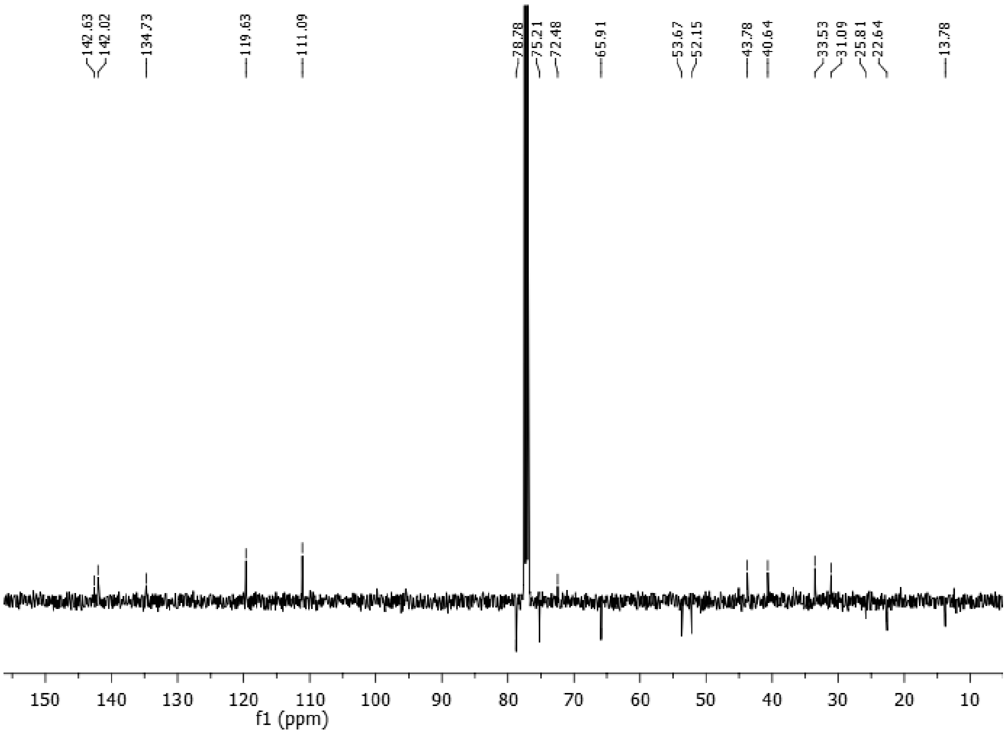

Figure S42. JMOD spectrum of **8**

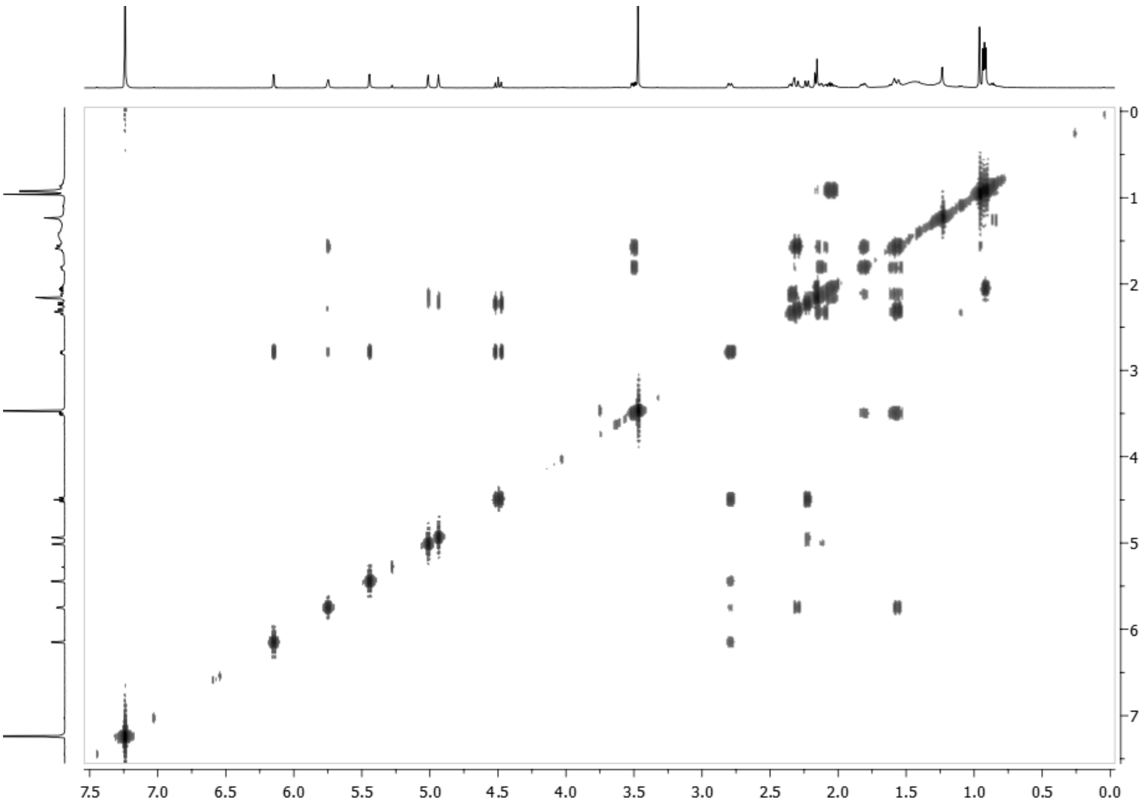

Figure S43. ^{1}H-^{1}H COSY spectrum of **8**

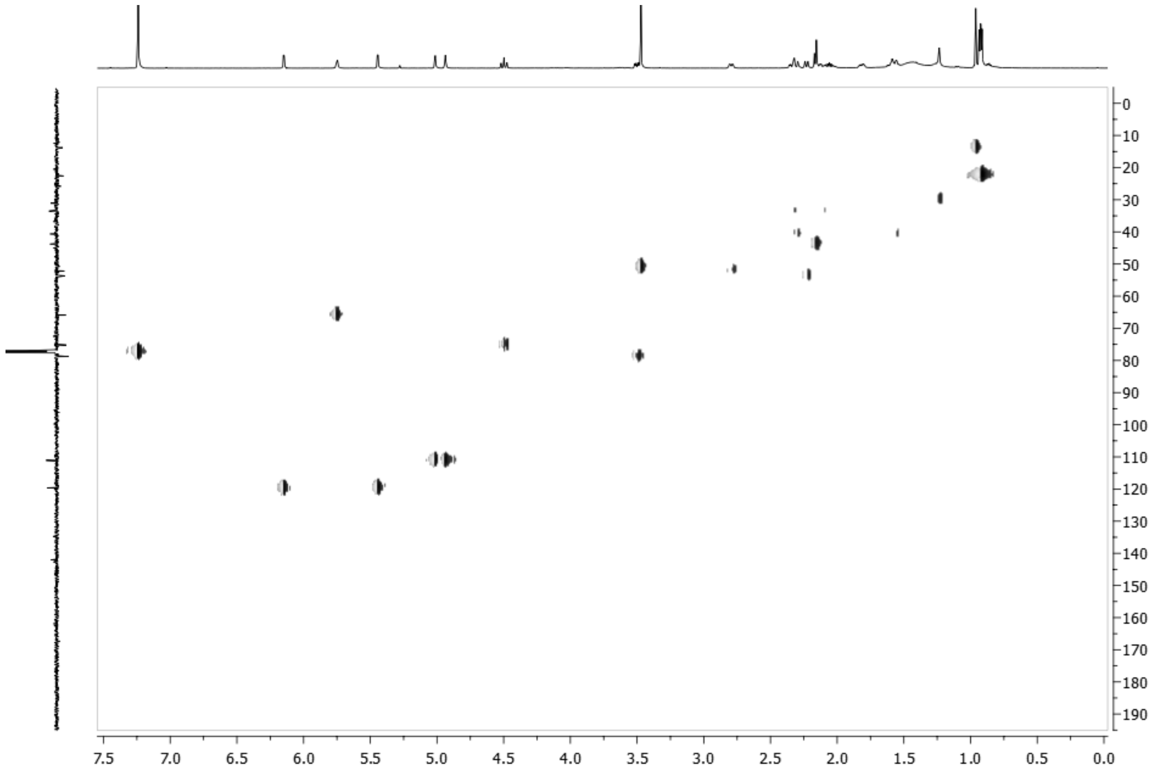

Figure S44. HSQC spectrum of **8**

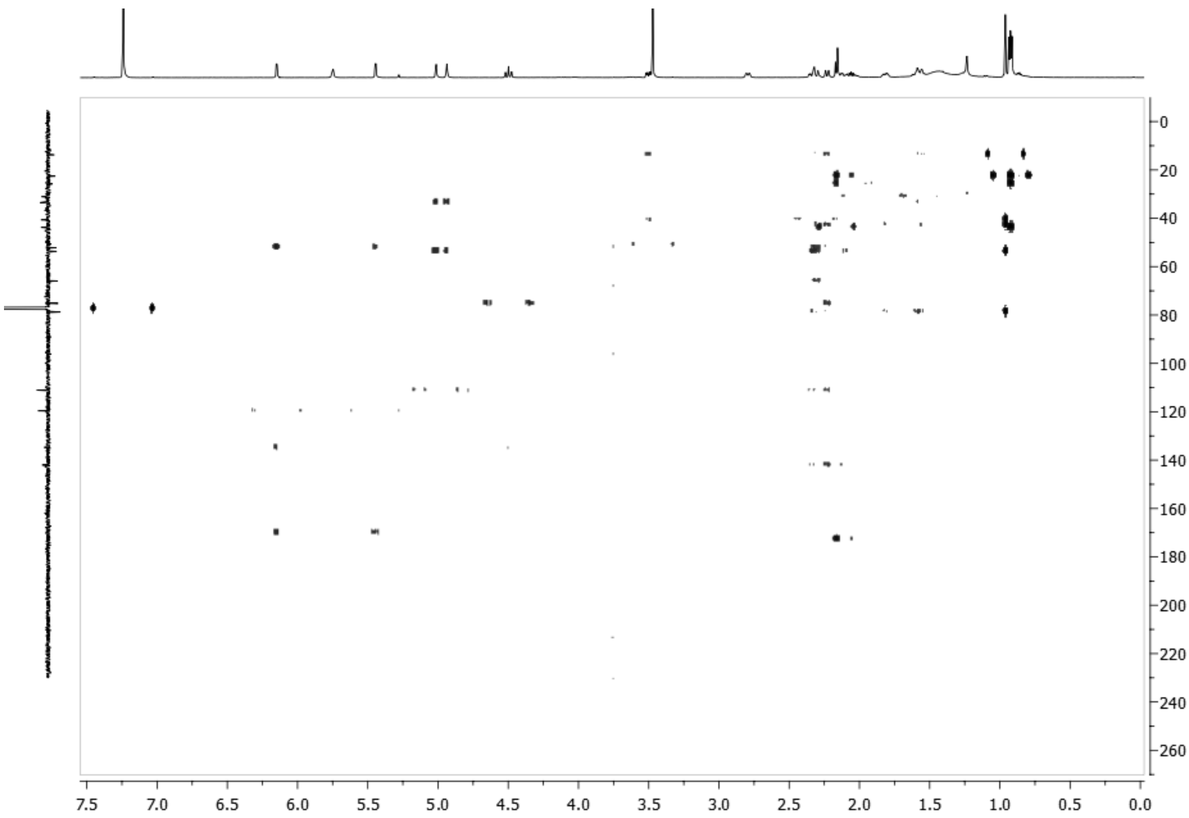

Figure S45. HMBC spectrum of **8**

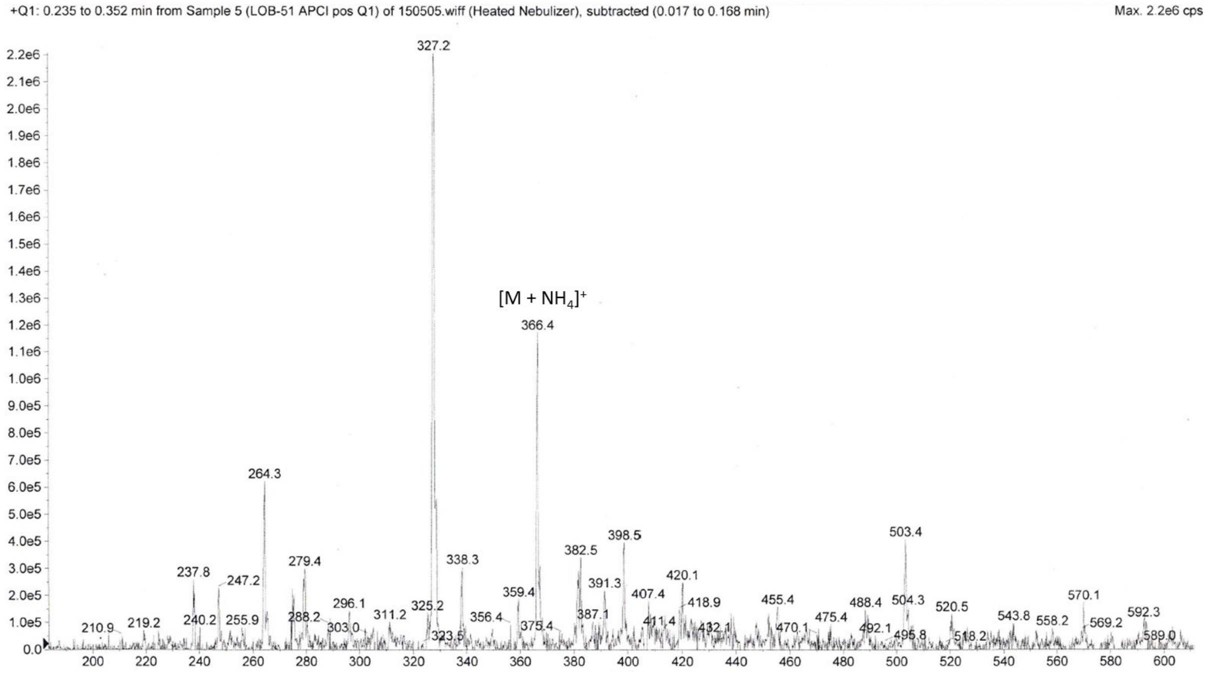

Figure S46. HRMS spectrum of **8**

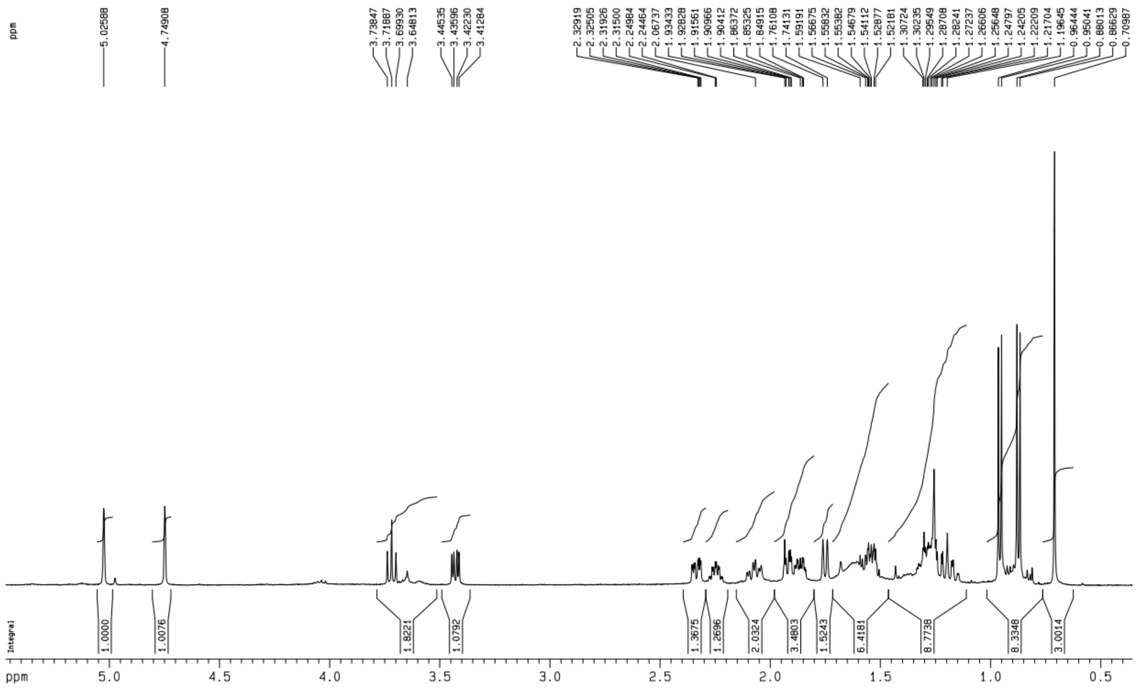

Figure S47. ^{1}H NMR spectrum of **9**

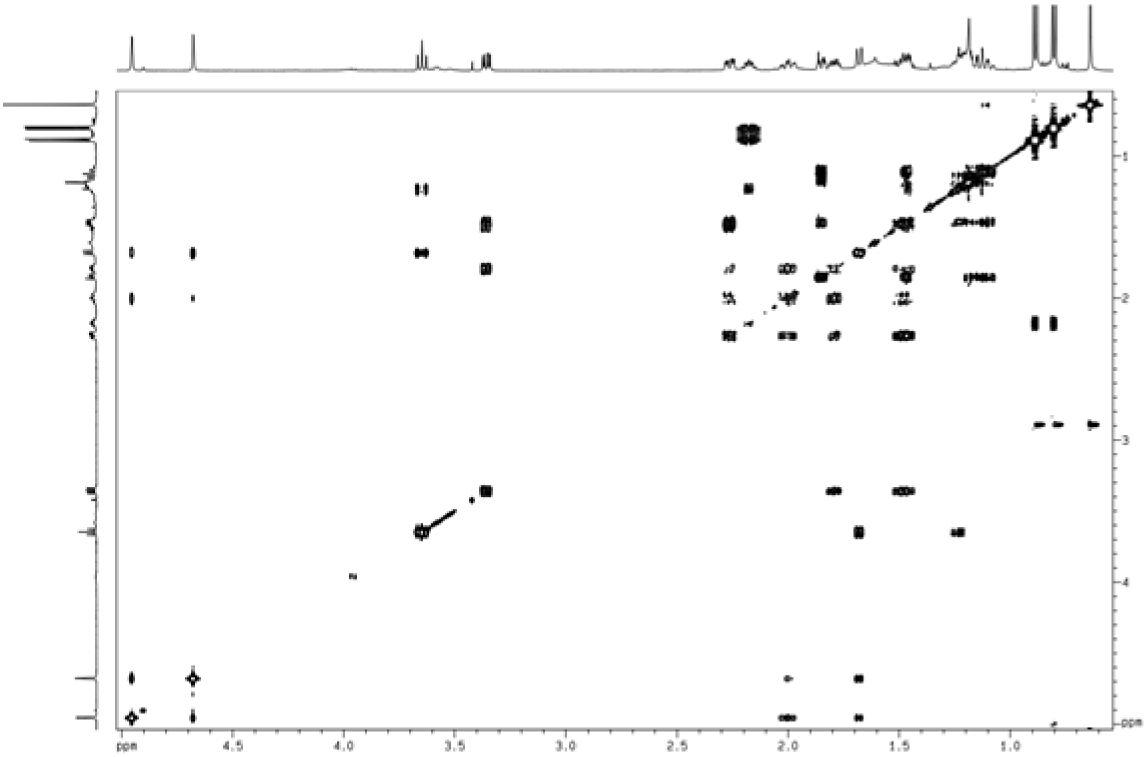

Figure S48. ^{1}H-^{1}H COSY spectrum of **9**

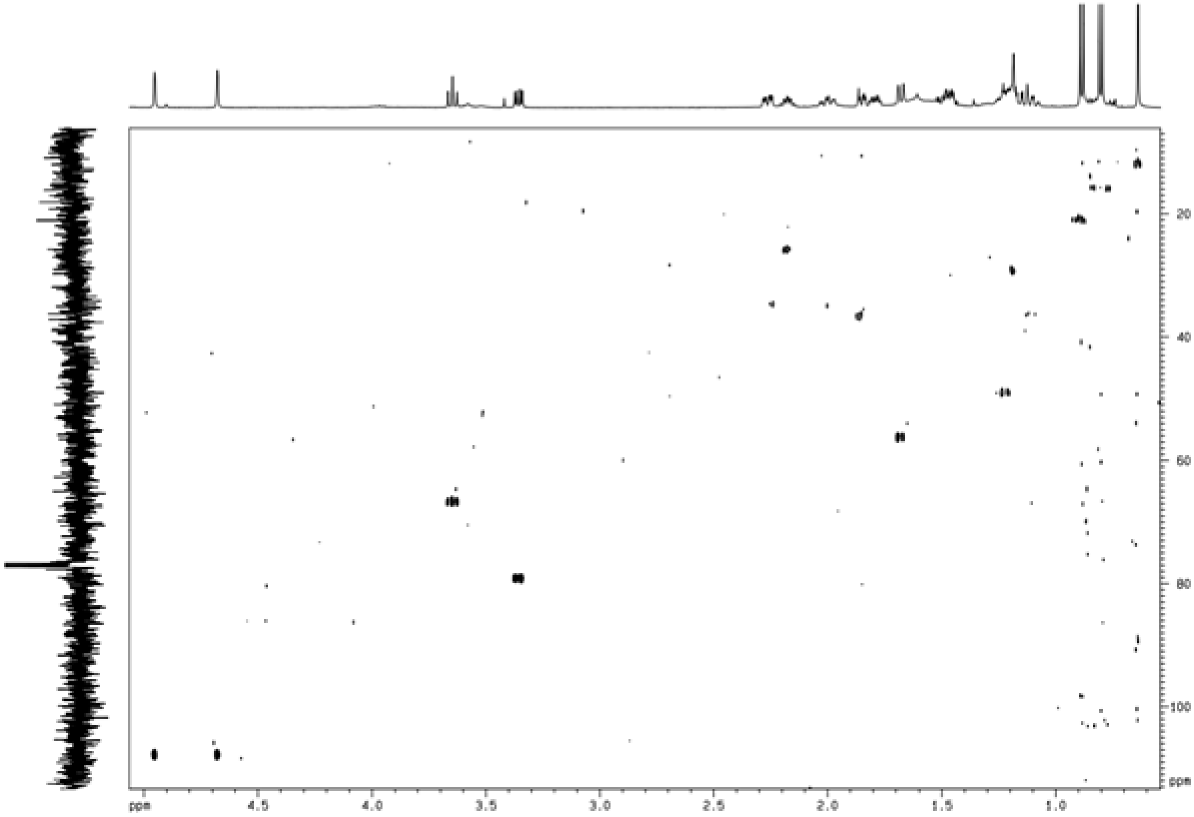

Figure S49. HSQC spectrum of **9**

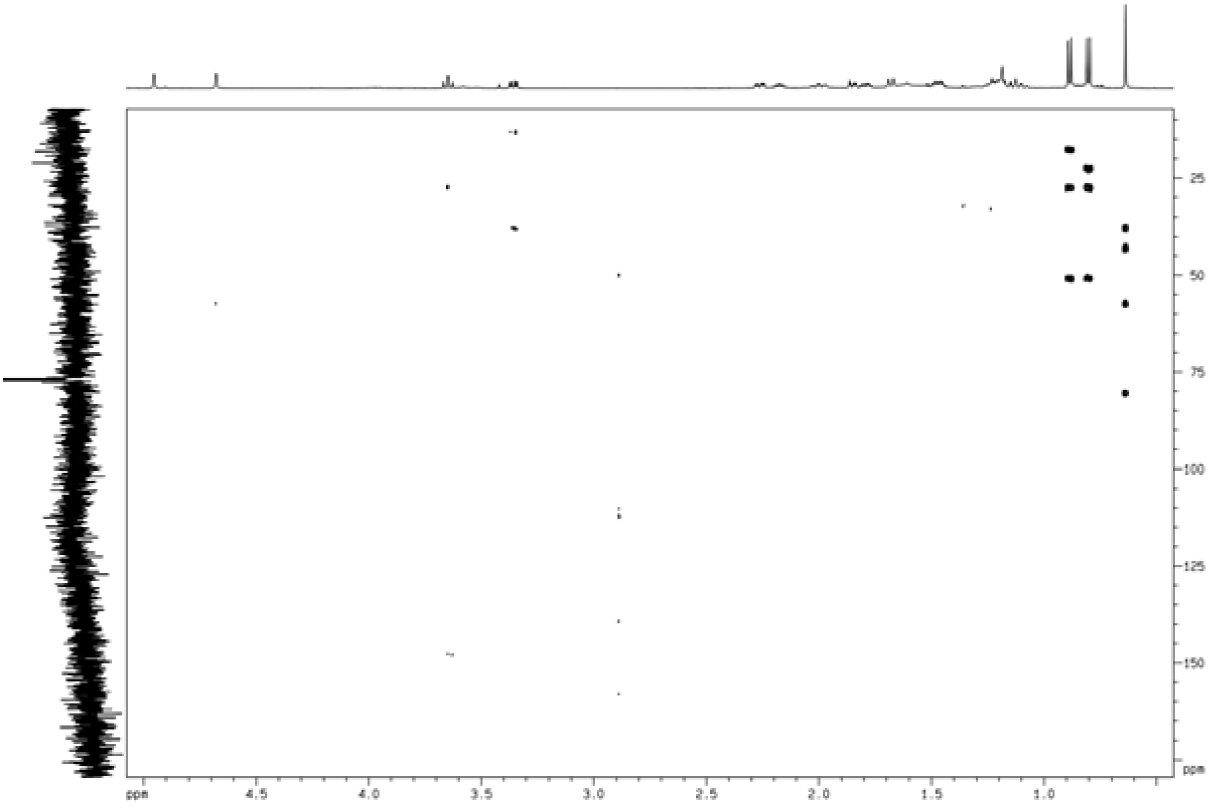

Figure S50. HMBC spectrum of **9**

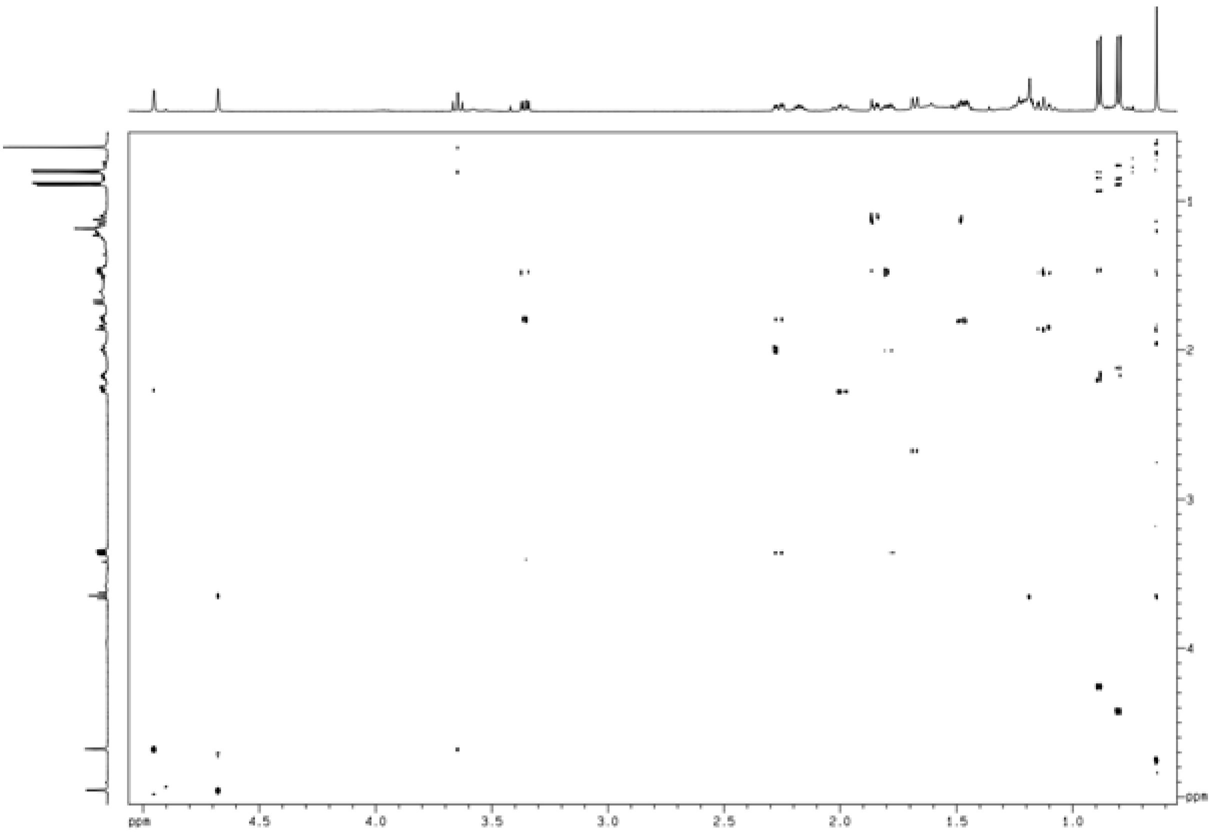

Figure S51. NOESY spectrum of **9**

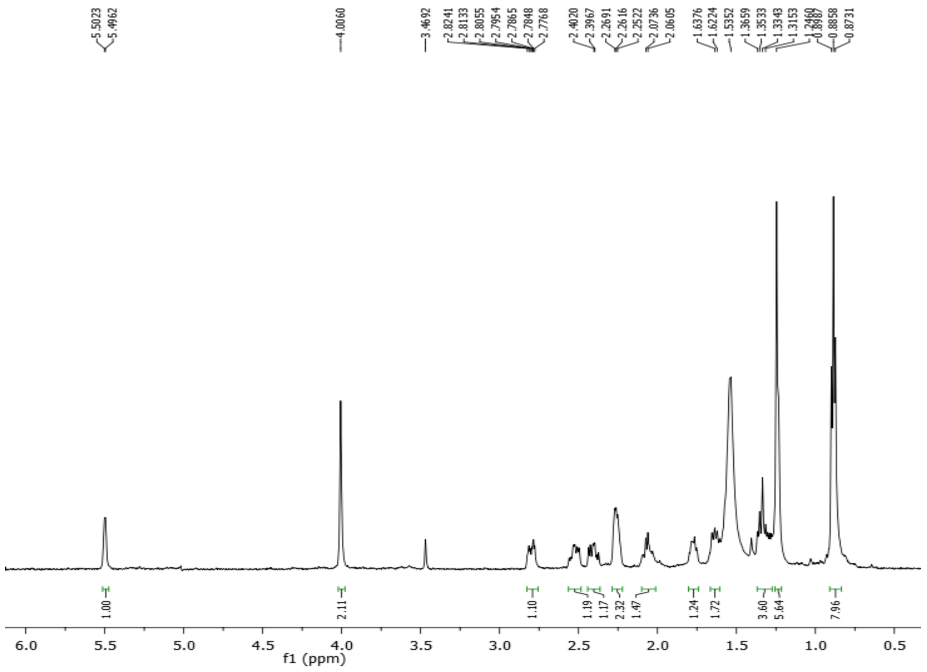

Figure S52. ^{1}H NMR spectrum of **10**

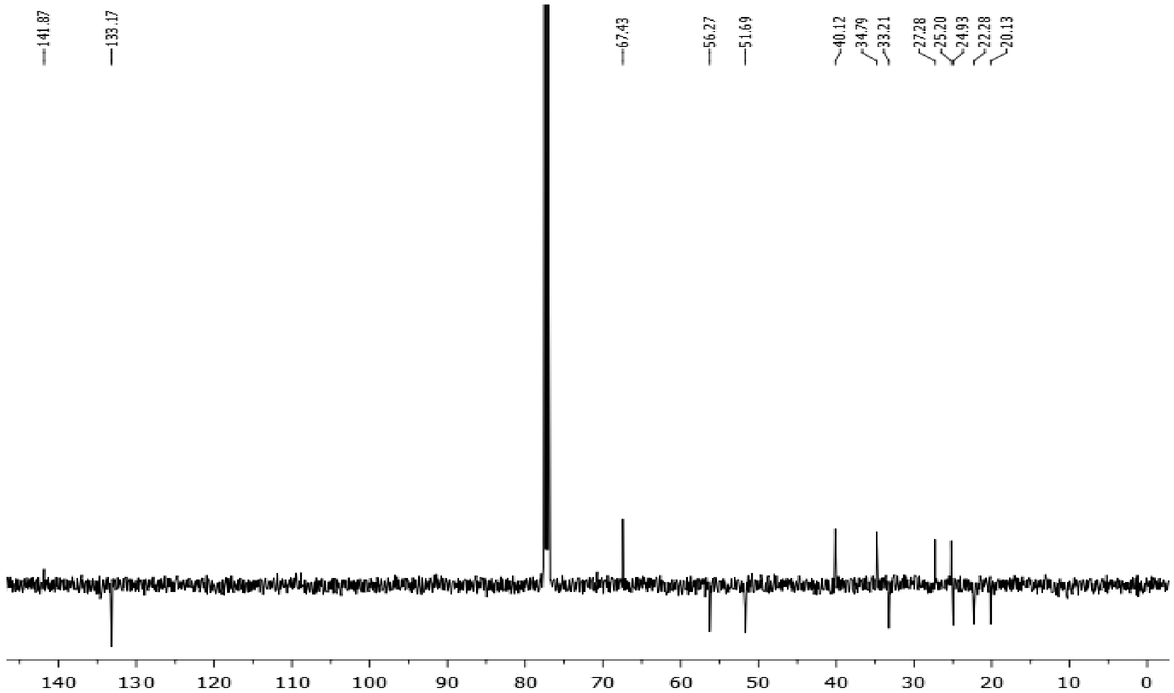

Figure S53. JMOD spectrum of **10**

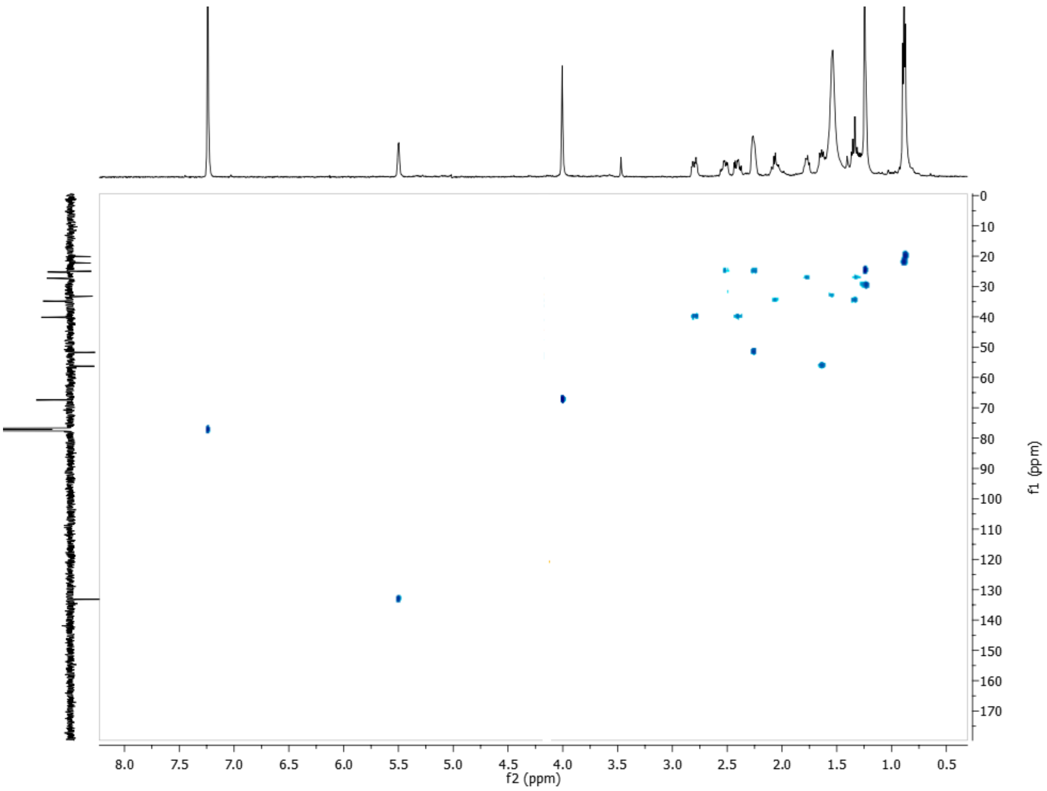

Figure S54. HSQC spectrum of **10**

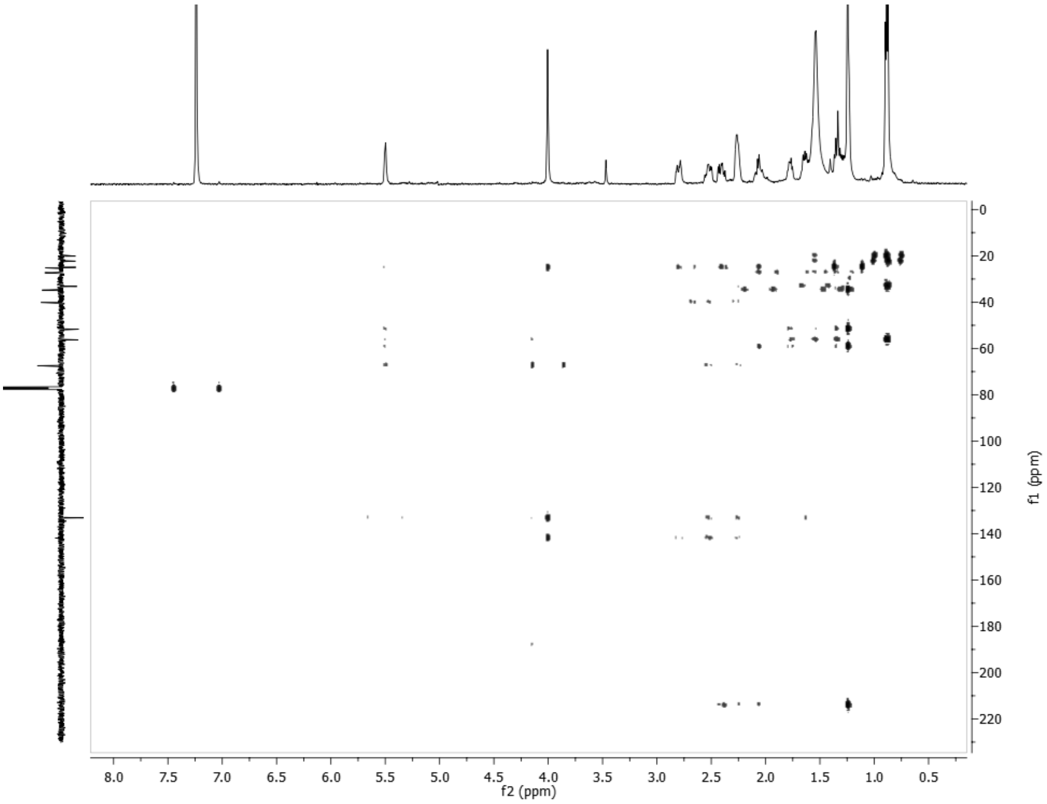

Figure S55. HMBC spectrum of **10**
